# IRE1 signaling increases PERK expression during chronic ER stress

**DOI:** 10.1038/s41419-024-06663-0

**Published:** 2024-04-18

**Authors:** Gideon Ong, Rosemund Ragetli, Katarzyna Mnich, Bradley W. Doble, Wafa Kammouni, Susan E. Logue

**Affiliations:** 1 Department of Human Anatomy and Cell Science, Max Rady College of Medicine, Rady Faculty of Health Sciences, University of Manitoba, Winnipeg, MB Canada; 2 Apoptosis Research Centre, University of Galway, Galway, Ireland; 3 School of Biological and Chemical Sciences, University of Galway, Galway, Ireland; 4 Department of Paediatrics, Department of Biochemistry and Medical Genetics, Max Rady College of Medicine, Rady Faculty of Health Sciences University of Manitoba, Winnipeg, MB Canada; 5 CancerCare Manitoba Research Institute, Winnipeg, MB Canada; 6 Children’s Hospital Research Institute of Manitoba, Winnipeg, MB Canada

**Keywords:** Stress signalling, Endoplasmic reticulum

## Abstract

The Unfolded Protein Response (UPR) is an essential cellular process activated by the accumulation of unfolded proteins within the Endoplasmic Reticulum (ER), a condition referred to as ER stress. Three ER anchored receptors, IRE1, PERK and ATF6 act as ER stress sensors monitoring the health of the ER. Upon detection of ER stress, IRE1, PERK and ATF6 initiate downstream signaling pathways collectively referred to as the UPR. The overarching aim of the UPR is to restore ER homeostasis by reducing ER stress, however if that is not possible, the UPR transitions from a pro-survival to a pro-death response. While our understanding of the key signaling pathways central to the UPR is well defined, the same is not true of the subtle signaling events that help fine tune the UPR, supporting its ability to adapt to varying amplitudes or durations of ER stress. In this study, we demonstrate cross talk between the IRE1 and PERK branches of the UPR, wherein IRE1 via XBP1s signaling helps to sustain PERK expression during prolonged ER stress. Our findings suggest cross talk between UPR branches aids adaptiveness thereby helping to support the plasticity of UPR signaling responses.

## Introduction

The Endoplasmic Reticulum (ER) functions as a cellular hub for protein folding and modification. Over 30% of proteins synthesized within the cell pass through the ER. Stresses which impact the ER environment (e.g. serum deprivation), or conditions which place excessive protein folding demands on the ER (e.g. viral infection) can negatively impact ER function leading to the accumulation of unfolded or misfolded proteins within the ER lumen, a condition commonly referred to as ER stress. Three ER anchored transmembrane receptors Inositol-Requiring Enzyme 1 Alpha (IRE1α, here after referred to as IRE1), Protein Kinase R (PKR)-like ER Kinase (PERK) and Activating Transcription Factor 6 (ATF6) monitor ER health. In healthy, unstressed cells, IRE1, PERK and ATF6 are held in an “off” state by binding of their luminal domain to the ER chaperone protein Glucose-Regulated Protein 78 (Grp78) [1, 2]. Accumulation of unfolded/misfolded proteins breaks this interaction thus permitting IRE1, PERK and ATF6 activation and initiation of their downstream signaling pathways, collectively referred to as the Unfolded Protein Response (UPR) [3–5]. The primary goal of the UPR is to reduce unfolded/misfolded proteins thereby restoring ER homeostasis.

Upon dissociation from Grp78, IRE1 dimerizes and trans-autophosphorylates facilitating activation of its RNase domain [6]. Through its RNase activity, IRE1 splices a 26-nucleotide intron from X-Box Binding Protein 1 (XBP1) mRNA. When religated, this encodes the transcription factor spliced XBP1 (XBP1s) [7, 8]. XBP1s aids restoration of ER homeostasis by increasing the expression of genes encoding ER chaperones and components of the ER associated degradation (ERAD) machinery [9]. In addition to splicing XBP1 mRNA, IRE1 RNase activity has also been linked to the selective degradation of mRNAs and miRNAs through a process referred to as Regulated IRE1 Dependent Decay (RIDD) [10, 11]. Within the context of ER stress resolution, RIDD-facilitated destruction of mRNAs encoding ER destined proteins helps prevent further demands being placed on an already compromised ER [10]. Similar to IRE1, PERK dimerizes and trans-autophosphorylates following Grp78 dissociation [12]. Active PERK, via its kinase activity, phosphorylates Serine 51 on Eukaryotic Initiation Factor 2α (eIF2α) resulting in a shut down in global cap-dependent translation, a key event in UPR signaling [5]. Upon Grp78 dissociation, ATF6 undergoes a conformational change facilitating its relocation from the ER to Golgi apparatus where it is cleaved by Site-1-protease and Site-2-protease generating a transcription factor linked to upregulation of XBP1 mRNA and genes encoding ER chaperone proteins [13, 14]. Collectively, UPR signaling mediators attempt to reduce ER stress by aiding refolding of those proteins that can be refolded and triggering the degradation of those proteins beyond repair. If successful, UPR signaling restores ER homeostasis. However, if ER stress is excessive or prolonged, UPR signaling switches from pro-survival to pro-death leading to ER stress-induced apoptosis [15].

Over the past 30 years, significant advances have been made in understanding IRE1, PERK and ATF6 dependent pathways, how they co-ordinate the UPR to aid ER stress resolution, and when dysregulated contribute to the progression of diseases such as cancer [16]. However, our understanding of the mechanisms that govern IRE1, PERK and ATF6 expression and modify UPR signaling, adjusting it to reflect different amplitudes or durations of ER stress is less refined. In this study, we investigate cross talk between IRE1, PERK and ATF6. Our results demonstrate that the ER stress sensor IRE1 helps to sustain PERK expression during chronic ER stress. This occurs through a mechanism facilitated by IRE1-XBP1s signaling, with IRE1-RIDD appearing to play no role. These findings increase our fundamental knowledge of the UPR and in particular, how expression of central ER stress sensors is fine-tuned.

## Materials and methods

### Antibodies and reagents

The following antibodies were used: IRE1 (Cell Signaling Technology, #3294, 1:2000), PERK (Cell Signaling Technology, #3192, 1:5000), ATF6 (Abcam, ab122897, 1:1000), ATF4 (Cell Signaling Technology, #11815, 1:2000), phospho-eIF2α (Cell Signaling Technology, #3398, 1:1000), eIF2α (Cell Signaling Technology, #5324, 1:5000), Caspase 3 (Cell Signaling Technology, #9662, 1:1000), Pan-Actin (Cell Signaling Technology, #4968, 1:5000), β-Actin (Cell Signaling Technology, #3700, 1:5000), XBP1s (BioLegend 143F, 1:1000), XBP1s (BioLegend, 9D11A43, 1:1000). Tunicamycin (11445), Thapsigargin (10522), Brefeldin A (11861) were acquired from Cayman Chemicals. MKC8866 was purchased from CSNPharm (CSN23751) or AmBeed (A1003533). KIRA6 (CSN21972) was obtained from CSNPharm. 4µ8C (A13803) was purchased from AdooQ. AMGEN 44 (Cat. No. 5517) was purchased from Tocris Bioscience. Calf Intestinal Alkaline Phosphatase (18009-019) was obtained from ThermoFisher. All chemicals and inhibitors were resuspended according to manufacturer’s instructions.

### Cell lines and culturing conditions

MDA-MB-231 cells (ATCC HTB-26) were cultured in high glucose DMEM (Gibco, 11965-092) supplemented with 10% fetal bovine serum (Gibco, 12483-020) and 2 mM GlutaMAX™ (Gibco, 35050-079). IRE1 knockout (KO) MDA-MB-231 cells and their respective wild-type counterparts were a gift from Dr. Christina Chan (Michigan State University) [17]. XBP1 KO MDA-MB-231 cells and their scrambled control counterparts were a gift from Dr. Afshin Samali (University of Galway) [18]. 4T1 cells (ATCC, CRL-2539, a gift from the Kung Lab, University of Manitoba) were grown in RPMI (21870-070) supplemented with 10% fetal bovine serum and 2 mM GlutaMAX™. MCF10a (ATCC, CRL-10317, a gift from Mowat lab, University of Manitoba) were cultured in HuMEC Basal serum free medium (Gibco, 12753018) to which HuMEC supplement mix (Gibco, 12755013) containing epidermal growth factor, hydrocortisone, isoproterenol, transferrin, and insulin, and 25 mg of bovine pituitary extract was added. All cell lines were cultured at 37 °C at 5% CO_2_ in a humidified incubator. Cells were routinely split through trypsinization and seeded at an appropriate density 24 h prior to treatment.

### Establishment of tetracycline inducible XBP1s MDA-MB-231 cells

MDA-MB-231 cells were transduced with lentiviral packaged tetracycline regulatory plasmid, pLV[Exp]-Neo-CMV>Tet3G (Vectorbuilder, USA). Transduced cells were selected by treatment with 900 µg/mL G418 (Sigma Aldrich, G8168). Surviving cells were then transduced with lentiviral packaged pLV-Puro-TRE3G > hXBP1s (Vectorbuilder, USA) and selected via culturing in medium supplemented with 1 µg/mL Puromycin (Sigma Aldrich, P8833). To induce XBP1s expression, stably selected cells were treated with doxycycline (Sigma Aldrich, D9891) at indicated concentrations.

### RNA extraction and qPCR

Total RNA was isolated using Monarch^®^ Total RNA Miniprep Kit extraction kit (NEB, T2010S) according to the manufacturer’s protocol. 0.25–1 μg of total RNA was reversed transcribed using the SensiFast cDNA synthesis kit (Meridian Bioscience, BIO-65054). qPCR reactions were conducted using PowerUp SYBR Green Master Mix (Thermo Fisher Scientific, A25742) and the QuantStudio3 thermocycler system. Annealing/extension reactions were carried out at 60 °C for 1 min followed by denaturation at 95 °C for 15 sec. Primer sequences are listed in Supplementary Table 1 (Table S1). Relative transcript levels were determined using the ΔΔCt method by normalizing target genes against *GAPDH* (human), *RPL10* (human) or *RPL13a* (mouse). The acquired ΔΔCt values were used to assess statistical significance between treatments.

### Chromatin immunoprecipitation (ChIP)

ChIP assays were performed using the SimpleChIP Enzymatic Chromatin IP Kit (Cell Signaling Technology, #9003) according to manufacturer’s recommendations. Following treatment, MDA-MB-231 cells were cross-linked with 37% formaldehyde (Sigma Aldrich, F8775) at a final concentration of 1% for 10 min at room temperature. Chromatin was digested by adding 1 µL of micrococcal nuclease (Cell Signaling Technology, #10011) per IP prep and incubation for 20 min at 37 °C. Samples were then subjected to sonication. ChIP was performed using anti-XBP1s (Cell Signaling Technology, #40435, 1:50) or normal rabbit IgG (Cell Signaling Technology, #2729) antibody. Immunoprecipitated DNA fragments were purified and analyzed by qPCR using primers designed against the promoter of *PERK* or exon 1 of *DNAJB9* (Cell Signaling Technology, #79879). Results were calculated using the percent input method. The acquired ΔCt values were used to assess statistical significance between treatments.

### Cell death analysis

MDA-MB-231 cells were trypsinized and plated on to a 96-well plate at a density of 5000 cells per well. Cells were allowed to adhere to the plate surface at room temperature for 20 min and transferred to the incubator for an additional 24 h before treatment. All treatments were prepared with complete growth medium containing either 250 nM (1:4000) IncuCyte^®^ Cytotox Red Reagent (Sartorius, 4632) or 5 µM (1:1000) IncuCyte^®^ Caspase 3/7 Green Dye (Sartorius, 4440) according to manufacturer’s recommendations. The first scan was taken using the IncuCyte^®^ S3 live-cell imaging system 30 min after treatment, followed by additional scans at 2 h intervals for 72 h. Three images of each well comprising of phase contrast (10x), red channel (400 ms) or green channel (300 ms) were obtained at each scan. Quantification of cell confluency and red or green signals were performed using the accompanying manufacturer software.

### Immunoblotting

Following treatment, cultured cells were scraped into media on ice. Cells were transferred into a 1.5 mL microcentrifuge tube and washed with ice-cold phosphate-buffered saline (PBS) twice. Whole-cell lysates were prepared using SDS lysis buffer (2% sodium dodecyl sulfate, 50 mM Tris-HCl (pH = 6.8), 0.05% Bromophenol Blue, 10% Glycerol, 5% 2-mercaptoethanol) or radioimmunoprecipitation assay (RIPA) buffer (25 mM Tris-HCl (pH = 7.4), 150 mM NaCl, 0.5% sodium deoxycholate, 0.1% sodium dodecyl sulfate, 1% Igepal, 0.5 mM DTT, 0.1 mM PMSF) supplemented with ROCHE cOmplete™, EDTA-free protease inhibitor (Sigma Aldrich, 4693132001). Protein concentration for samples lysed by RIPA buffer were quantified using BCA assay (ThermoFisher, 23225). Samples lysed by RIPA buffer were further supplemented with Laemelli buffer (1% SDS, 10% glycerol, 0.02% Bromophenol Blue, 50 mM Tris-HCl (pH = 6.8), 1% 2-mercaptoethanol). Afterwards, lysates were boiled at 95 °C for 5 min. For PERK and IRE1 dephosphorylation, calf-intestinal alkaline phosphatase (CIAP) treatment was performed following lysis with RIPA buffer. Samples were treated with the supplied 10× dephosphorylation buffer and approximately 1 unit of CIAP was added per 1 μg of protein. Lysates were incubated with CIAP at 37 °C for 1 h. Samples were supplemented with Laemelli buffer and boiled at 95 °C for 5 min. Protein lysates were loaded onto BioRad Stain-Free™ FastCast™ acrylamide gels (BioRad, #1610183), semi-dry transferred onto 0.2 µm nitrocellulose membranes (BioRad, #1620112) and blocked in PBS-0.1% Tween containing 5% skim milk. Acquisition of chemiluminescent signal was performed using the ChemiDoc system (BioRad). Densitometric analysis was carried out using ImageLab 6.0.1 (BioRad).

### Statistical analysis

All data are displayed as mean ± standard deviation (SD) or mean ± standard error of mean (SEM). Statistical analyses were conducted using GraphPad Prism 9. Where appropriate, one-way ANOVA or two-way ANOVA followed by Tukey HSD post-Hoc analysis was used to assess statistical significance amongst treatments. Values with *P* ≤ 0.05 were considered statistically significant.

## Results

### ER stress triggers dynamic alterations in IRE1 and PERK expression

**Fig. 1:**
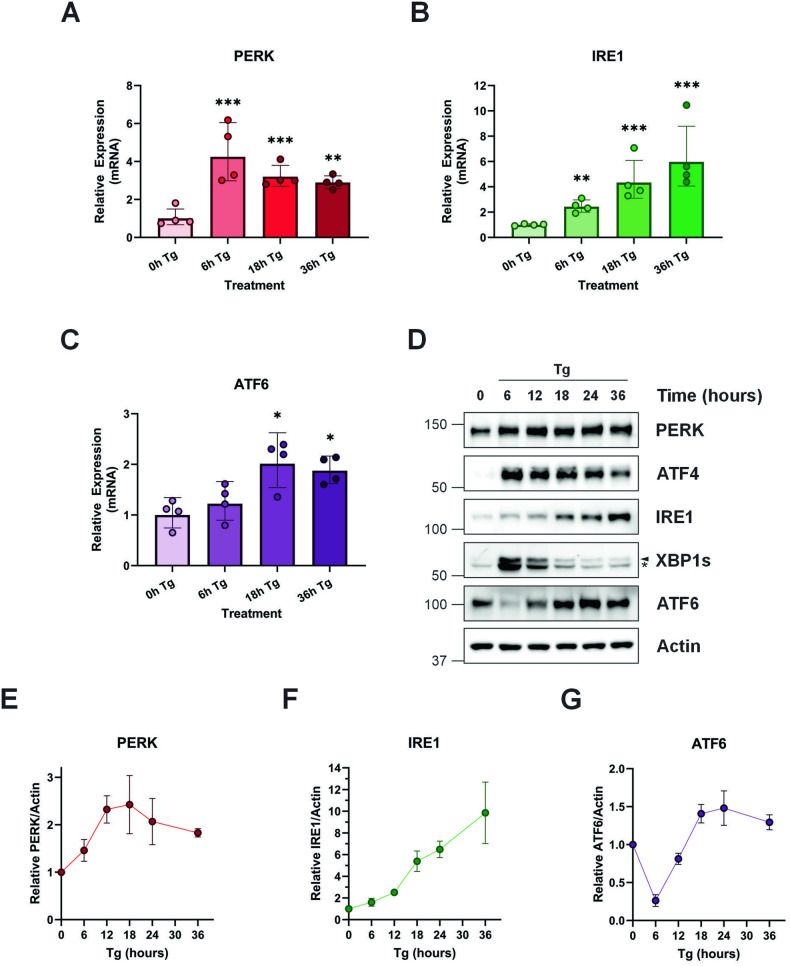
ER stress induces dynamic regulation of IRE1, PERK and to a lesser extent ATF6 expression. MDA-MB-231 cells were treated for the indicated times with 0.5 μM Tg after which cells where harvested. RNA was extracted and qPCR used to assess the relative expression of *PERK* (**A**), *IRE1* (**B**) and *ATF6* (**C**) transcripts. Mean relative expression ± SD, reference gene *GAPDH*, *N* = 4. **D** Cell lysates were treated with calf-intestinal alkaline phosphatase (to dephosphorylate PERK and IRE1) and analyzed via immunoblotting for IRE1, XBP1s, PERK, ATF4 and ATF6 with (**E–G**) relative changes compared to 0 h determined by densitometry (mean relative expression ± SEM). Actin was used as a loading control. Blots are representative of *N* = 3. Statistical significance was determined using one-way ANOVA followed by TUKEY HSD post-hoc analysis (qPCR). *p ≤ 0.05, **p ≤ 0.01, ***p ≤ 0.001 vs 0 h Tg.

MDA-MB-231 cells were subjected to treatment with the chemical inducer of ER stress, thapsigargin (Tg), for various time points up to 36 h after which expression of IRE1, PERK and ATF6 was analyzed via qPCR and immunoblotting. Thapsigargin treatment triggered a rapid fourfold increase in PERK transcript (6 h), which was maintained throughout the remainder of the 36 h timecourse (Fig. 1A). Likewise, IRE1 transcript increased following Tg treatment. However, the pattern of regulation differed somewhat from PERK, starting with a more modest twofold increase (6 h), which continued to intensify reaching a sixfold increase by 36 h Tg (Fig. 1B). Unlike IRE1 and PERK, ATF6 transcript levels were relatively stable throughout the 36 h timecourse displaying a more modest twofold increase by 18 h (Fig. 1C). Expression of IRE1, PERK and ATF6 protein levels were subsequently assessed by immunoblotting (Fig. 1D). To compare and quantify total PERK protein, lysates were treated with alkaline phosphatase. Analysis of protein expression displayed a similar trend, with expression of PERK and IRE1 increasing throughout the 36 h timecourse (Fig. 1D–F). ATF6 was rapidly processed as shown by the loss of full length ATF6, but as the timecourse shifted from acute to chronic ER stress, ATF6 processing diminished (Fig. 1D, G). Collectively, these results demonstrate ER stress instigates dynamic alterations in the expression of IRE1, PERK and to a lesser extent ATF6.

### Inhibition of PERK signaling during ER stress reduces IRE1 expression

Previous work by Tsuru *et al*. reported an ER stress driven cross talk wherein PERK via Activating Transcription Factor 4 (ATF4) increased IRE1 expression [19]. Given that PERK expression rapidly increased in our system, we questioned if addition of a PERK inhibitor would reduce IRE1 expression and limit its downstream signaling. Combination with the PERK inhibitor Amgen 44 blocked Tg-induced PERK signaling as demonstrated both by a downshift in PERK protein (indicative of reduced activation) and a decrease in phosphorylated eIF2α (Supplementary Fig. 1). Alongside these changes, we also observed a decrease in IRE1 protein expression and reduced appearance of XBP1s (Supplementary Fig. 1). This observation confirmed the existence of cross talk between PERK and IRE1 in our system and lead us to question if IRE1 could exert a reciprocal regulation upon PERK.

### Inhibition of IRE1 signaling lowers PERK protein expression during chronic ER stress

**Fig. 2:**
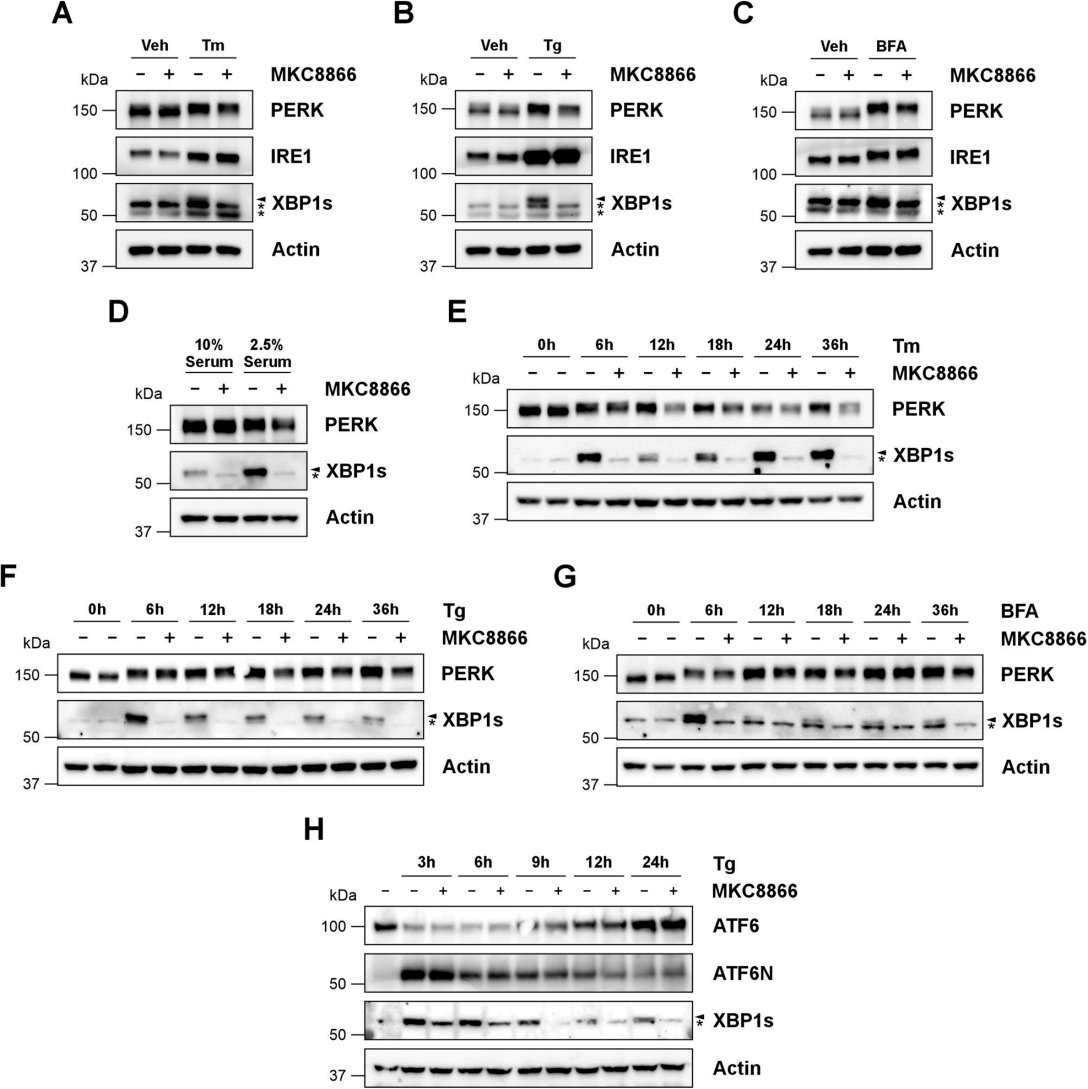
Combination of MKC8866 with diverse chemical and physiological inducers of ER stress lowers PERK expression. MDA-MB-231 cells were treated with **A** Tm (1.92 μM, 18 h), **B** Tg (0.5 μM, 18 h), **C** BFA (0.2 μM, 18 h) or **D** serum deprivation (2.5%, 72 h) alone or in combination with MKC8866 (20 μM) after which cell lysates were collected and immunoblotted for IRE1, XBP1s and PERK. MDA-MB-231 cells were treated for the indicated timepoints with **E** Tm (1.92 μM), **F**, **H** Tg (0.5 μM) or **G** BFA (0.2 μM) alone or in combination with MKC8866 (20 μM) after which cells were harvested, lysed and immunoblotted for XBP1s, PERK (**E–G**) or ATF6 (**H**). Arrow denotes XBP1s band, * indicates non-specific band(s). Actin was used as a loading control. Blots are representative of *N* = 3.

To assess the impact of IRE1 signaling upon PERK expression, MDA-MB-231 cells where subjected to a range of ER stress inducing agents including Tg, tunicamycin (Tm) and Brefeldin A (BFA) for 18 h in the presence or absence of the IRE1 inhibitor MKC8866. MKC8866 is a salicylaldehyde analog that is a selective, potent, and reversible inhibitor of IRE1 RNase activity [20]. Treatment with Tg, Tm or BFA triggered robust UPR signaling in MDA-MB-231 cells, as indicated by the appearance of XBP1s (indicative of IRE1 activation) and by a PERK upshift (indicative of PERK phosphorylation and activation) (Fig. 2A–C). Combination with MKC8866 clearly suppressed ER stress-induced XBP1 splicing and lowered PERK protein expression (Fig. 2A–C). In addition to triggering ER stress through the use chemical inducers, we also assessed the impact of physiological inducers of ER stress. MDA-MB-231 cells cultured in low serum (2.5%) for 72 h displayed robust UPR activation as demonstrated by the appearance of XBP1s (IRE1 activation) and upshift in PERK protein (Fig. 2D). MKC8866 addition, while clearly attenuating IRE1 RNase activity in low serum treated cells, also reduced PERK protein expression (Fig. 2D).

To expand this observation, we repeated experiments using Tg, Tm and BFA alone or in combination with MKC8866 ranging treatment times from 6 h to 36 h. Again, we found that combination of ER stress inducers with MKC8866, while blocking IRE1 signaling (as shown by loss of XBP1s), also lowered PERK protein expression with the degree of reduction in PERK expression becoming more apparent at those later timepoints representative of chronic, long-term ER stress (Fig. 2E–G). In addition to examining the PERK arm of the UPR, we also assessed ATF6 expression. Unlike PERK, neither ATF6 expression nor Tg induced ATF6 processing was impacted by MKC8866 addition (Fig. 2H).

**Fig. 3:**
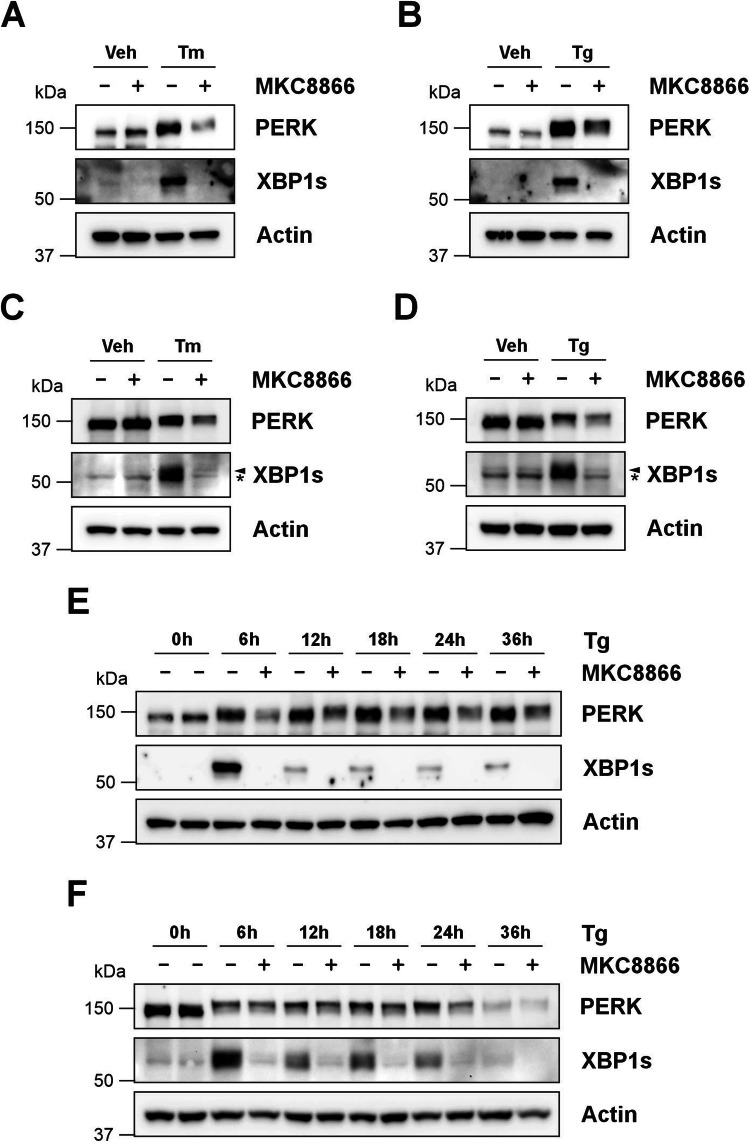
MKC8866 mediated reduction of PERK during ER stress is detectable in multiple cell lines. **A**, **B** MCF10a and **C**, **D** 4T1 cells were treated with Tg (0.5 μM) (**B**, **D**) or Tm (1.92 μM) (**A**, **C**) alone or in combination with MKC8866 (20 μM) for 18 h. Cell lysates were immunoblotted for PERK and XBP1s. **E** MCF10A cells or **F** 4T1 cells were treated for the indicated timepoints with Tg (0.5 μM) alone or in combination with MKC8866 (20 μM) and cell lysates immunoblotted for PERK and XBP1s. Actin was used as a loading control. Blots are representative of *N* = 3. Arrow denotes XBP1s band, * indicates non-specific band(s).

While these results demonstrated combination of MKC8866 with a range of chemical or physiological inducers of ER stress reduced expression of PERK, whether this observation was restricted to MDA-MB-231 cells was not known. To answer this, we repeated experiments combining chemical inducers of ER stress with MKC8866 in two additional cell lines, namely MCF10a cells (human non-tumorigenic breast epithelial cell line) and 4T1 cells (murine triple negative breast cancer (TBNC) cell line). Treatment of 4T1 and MCF10a cells with Tm or Tg triggered robust activation of IRE1 and PERK (Fig. 3A–F). Similar to MDA-MB-231 cells, combination of Tm or Tg with MKC8866 blocked IRE1 signaling and reduced PERK protein expression (Fig. 3A–F). These results indicate MKC8866 mediated suppression of PERK protein expression during ER stress is not restricted to MDA-MB-231 cells.

**Fig. 4:**
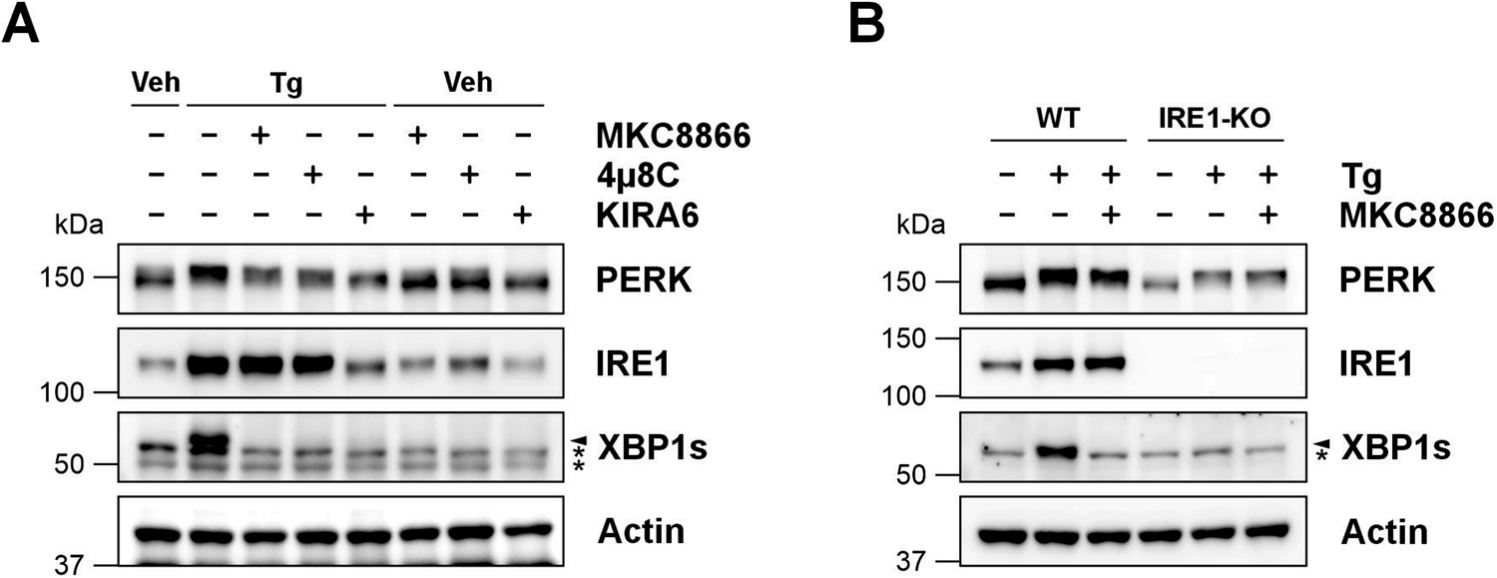
Ablation of IRE1 signaling via pharmacological based or genetic strategies reduces PERK expression during sustained ER stress. **A** MDA-MB-231 cells and **B** IRE1 knockout (IRE1-KO) or corresponding wild-type (WT) MDA-MB-231 cells were treated for 18 h with Tg (0.5 μM) alone or in combination with **A** MKC8866 (20 μM), 4μ8c (32 μM) or KIRA6 (5 μM) or **B** MKC8866 (20 μM). Cell lysates were harvested and immunoblotted for IRE1, XBP1s and PERK. Actin was used as a loading control. Blots are representative of *N* = 3. Arrow denotes XBP1s band, * indicates non-specific band(s).

Although MKC8866 is a widely used selective inhibitor of IRE1, we asked if the reduction in PERK observed upon combination with MKC8866 was exclusive to MKC8866 or true of IRE1 inhibitors in general. To answer this, two additional IRE1 inhibitors, 4μ8c and KIRA6 were employed. While 4μ8c is similar to MKC8866 in its mechanism of action (binds directly to the RNase domain of IRE1), KIRA6 is different in that it prevents IRE1 kinase activity and by doing so suppresses activity of the IRE1 RNase domain [21, 22]. As observed with MKC8866, combination of 4μ8c or KIRA6 with Tg, in addition to blocking IRE1 activity (reduced XBP1s), also lowered expression of PERK protein (Fig. 4A). To complement findings generated with IRE1 inhibitors, IRE1 knockout (KO) and corresponding wild-type MDA-MB-231 cells were treated with Tg for 18 h alone or in combination with MKC8866. Tg induced robust IRE1 and PERK activation in wild-type cells as demonstrated by appearance of XBP1s and PERK upshifts (Fig. 4B). While PERK was still activated (determined by an upshift), the level of PERK protein in IRE1 KO cells was significantly diminished compared to wild-type counterparts (Fig. 4B). MKC8866 addition did not further suppress PERK expression in IRE1 KO MDA-MB-231 cells, indicating MKC8866 mediated reductions in PERK expression are a consequence of inhibited IRE1 signaling (Fig. 4B). Collectively, these results indicate that during long-term chronic ER stress, IRE1 signaling helps to support PERK expression.

### IRE1 RNase activity increases PERK transcription during ER stress via IRE1-XBP1s signaling

**Fig. 5:**
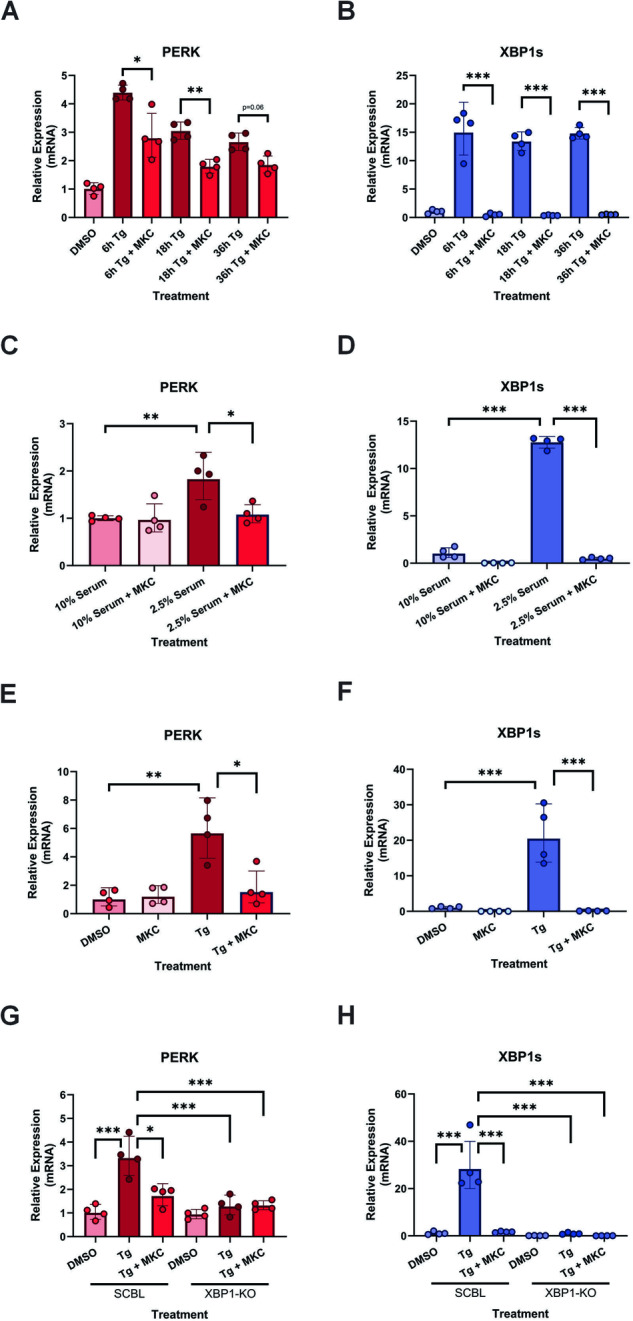
ER stress mediated increases in PERK transcript occur in a manner partially dependent upon IRE1 RNase signaling. MDA-MB-231 cells were treated with Tg (0.5 μM) (**A**, **B**) or cultured under serum deprivation (2.5% serum, 72 h) (**C**, **D**) for the indicated timepoints alone or in combination with MKC8866 (MKC, 20 μM). Extracted RNA was utilized for qPCR assessment of relative changes in (**A**, **C**) *PERK* and (**B**, **D**) *XBP1s* transcript. Mean relative expression ± SD, reference gene (**A**, **B**) *GAPDH* and (**C**, **D**) *RPL10*, *N* = 4. **E**, **F** 4T1 cells were treated with Tg (0.5 μM) alone or in combination with MKC8866 (MKC, 20 μM) for 18 h following which RNA was extracted and relative changes in (**E**) *PERK* and (**F**) *XBP1s* transcript assessed. Mean relative expression ± SD, reference gene *RPL13a*, *N* = 4. **G**, **H** Scrambled control (SCBL) and XBP1 knockout (XBP1-KO) MDA-MB-231 cells were treated with Tg (0.5 μM) alone or in combination with MKC8866 (MKC, 20 μM) for 18 h after which RNA was extracted and relative expression changes in *PERK* (**G**) and *XBP1s* (**H**) assessed by qPCR. Mean relative expression ± SD, reference gene *GAPDH*, *N* = 4. Statistical significance for all qPCR experiments was determined using one-way ANOVA followed by TUKEY HSD post-hoc analysis. *p ≤ 0.05, **p ≤ 0.01, ***p ≤ 0.001.

To understand the mechanism through which IRE1 signaling can help sustain PERK expression, we assessed alterations to PERK transcript in the presence or absence of MKC8866. Similar to results presented in Fig. 1, increases in PERK transcript were evident as early as 6 h post Tg treatment and maintained throughout the 36 h timecourse (Fig. 5A). Addition of MKC8866, while blocking IRE1 signaling as indicated by a reduction in XBP1s transcript in Tg treated MDA-MB-231 cells, also reduced PERK transcript levels (Fig. 5A, B). A similar pattern of IRE1 dependent increases in PERK transcript was observed in MDA-MB-231 cells exposed to serum deprivation and murine 4T1 cells treated with Tg alone or in combination with MKC8866 (Fig. 5C–F).

**Fig. 6:**
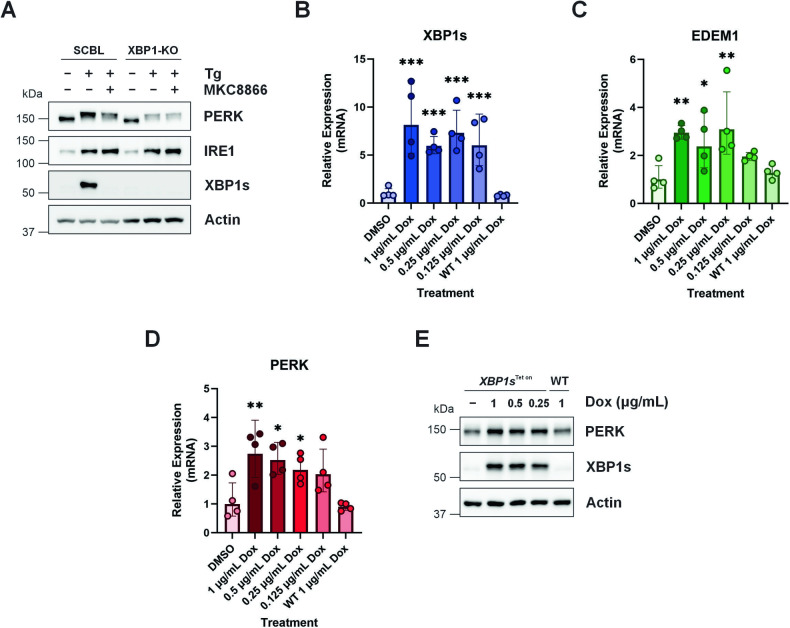
IRE1-XBP1s signaling increases PERK expression. **A** Scrambled control (SCBL) and XBP1 knockout (XBP1-KO) MDA-MB-231 cells were treated with Tg (0.5 μM) alone or in combination with MKC8866 (20 μM) for 18 h. After which, cells were harvested, lysed, and immunoblotted for PERK, IRE1 and XBP1s. Actin was used as a loading control. Blots are representative of *N* = 3. MDA-MB-231 XBP1s^Tet on^ or wild-type (WT) cells were treated with doxycycline (Dox) for 18 h after which (**B–D**) RNA was extracted and relative expression changes in *XBP1s* (**B**), *EDEM1* (**C**) *and PERK* (**D**) assessed by qPCR. Mean relative expression ± SD, reference gene *GAPDH*, *N* = 4. Statistical significance for all qPCR experiments was determined using one-way ANOVA followed by TUKEY HSD post-hoc analysis. *p ≤ 0.05, **p ≤ 0.01, ***p ≤ 0.001. **E** Cells were harvested, lysed, and immunoblotted for PERK and XBP1s. Actin was used as a loading control. Blots are representative of *N* = 3.

Activation of IRE1 RNase signaling is associated with two signaling outputs, splicing of XBP1 mRNA generating XBP1s, and Regulated IRE1 Dependent Decay (RIDD). Addition of IRE1 inhibitors or loss of IRE1 expression blocks both XBP1 splicing and RIDD. To differentiate between these signaling outcomes, and determine if the increases in PERK transcript were an outcome of IRE1-XBP1s, IRE1-RIDD or a combination of both we utilized XBP1 KO MDA-MB-231 cells. As shown in Supplementary Fig. 2, treatment of XBP1 KO MDA-MB-231 cells with Tg reduced expression of the RIDD target *DGAT2*. Combination with the IRE1 RNase inhibitor MKC8866 suppressed Tg-mediated IRE1-RIDD signaling as demonstrated by increased *DGAT2* expression, thus confirming the functionality of XBP1 KO MDA-MB-231 cells as a means to differentiate between IRE1-XBP1s and IRE1-RIDD signaling (Supplementary Fig. 2). XBP1 KO MDA-MB-231 cells when treated with Tg, unlike their scrambled control counterparts, failed to increase PERK transcript (Fig. 5G, H). Likewise, analysis of PERK protein expression in Tg treated control and XBP1 KO MDA-MB-231 cells revealed a similar outcome with scrambled MDA-MB-231 cells displaying elevated PERK expression compared to their XBP1 KO counterparts (Fig. 6A). Addition of MKC8866, while suppressing PERK protein expression in scrambled MDA-MB-231 cells, did not alter PERK protein levels in XBP1 KO cells, indicating a reliance upon IRE1-XBP1s signaling (Fig. 6A). To further interrogate the relationship between XBP1s and PERK, we established tetracycline inducible XBP1s MDA-MB-231 cells (MDA-MB-231 XBP1s^Tet on^). Addition of doxycycline to MDA-MB-231 XBP1s^Tet on^ cells initiated robust induction of XBP1s transcript and protein (Fig. 6B, E). To confirm XBP1s functionality in this system, expression of the XBP1s target gene EDEM1 was monitored. Induction of XBP1s, via doxycycline treatment, resulted in a significant increase in EDEM1 transcript verifying XBP1s functionality (Fig. 6C). Similar to EDEM1, selective expression of XBP1s elevated PERK transcript (Fig. 6D) and protein expression (Fig. 6E), underscoring the relationship between XBP1s and PERK.

**Fig. 7:**
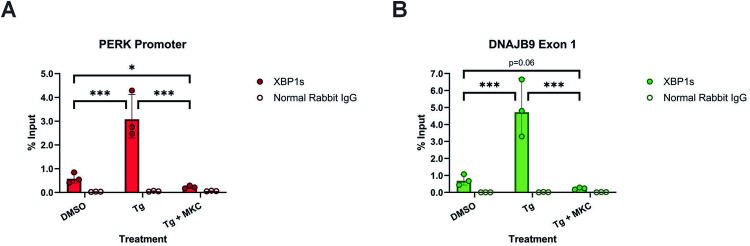
Induction of ER stress increases XBP1s binding to the promoters of downstream target genes. Chromatin immunoprecipitations were performed with cross-linked chromatin from MDA-MB-231 cells treated with Tg (0.5 μM) alone or in combination with MKC8866 (MKC, 20 μM) for 18 h using either XBP1s or control rabbit IgG antibodies. The enriched DNA was quantified by qPCR using primers designed against (**A**) the promoter of *PERK* or (**B**) exon 1 of *DNAJB9*. The amount of immunoprecipitated DNA in each sample is represented as signal relative to the total amount of input chromatin, which is equivalent to one. Mean percent signal relative to input ± SD, *N* = 3. Statistical significance for all qPCR experiments was determined using one-way ANOVA followed by TUKEY HSD post-hoc analysis. *p ≤ 0.05, ***p ≤ 0.001.

As XBP1s is a transcription factor, we next asked if XBP1s can regulate PERK expression through binding to the PERK promoter. To answer this, MDA-MB-231 cells treated with Tg alone or in combination with MKC8866, were subjected to chromatin immunoprecipitation (ChIP) assays using anti-XBP1s antibody and binding assessed via ChIP-qPCR. As shown in Fig. 7, ChIP-qPCR identified XBP1s binding to the *PERK* promoter in Tg treated cells, which was reversed upon the addition of MKC8866 (Fig. 7A). A similar pattern of interaction was observed with the known XBP1s target gene *DNAJB9* (Fig. 7B).

### IRE1 inhibition reduces PERK expression and lowers PERK mediated downstream signaling during ER stress

**Fig. 8:**
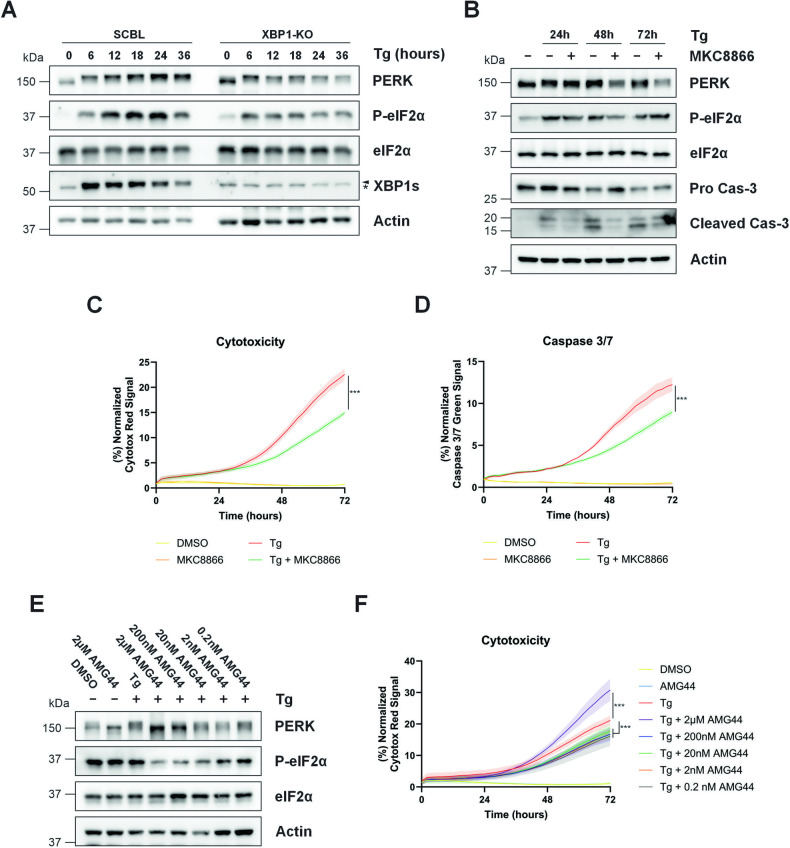
Suppression of the IRE1-XBP1s axis lowers PERK signaling in cells subjected to ER stress. **A** Scrambled control (SCBL) and XBP1 knockout (XBP1-KO) MDA-MB-231 cells were treated with Tg (0.05 μM) alone or in combination with MKC8866 (20 μM) for the indicated timepoints after which PERK, P-eIF2α, eIF2α and XBP1s expression was assessed via immunoblotting. Arrow denotes XBP1s band, * indicates non-specific band(s). Actin was used as a loading control. Blots are representative of *N* = 3. **B–D** MDA-MB-231 cells were subjected to Tg (0.5 μM) treatment alone or in combination with MKC8866 (20 μM) for up to 72 h after which PERK, P-eIF2α, eIF2α, pro-caspase-3 and cleaved caspase-3 expression was assessed by immunoblotting (**B**), while cell death (**C**) and caspase-3/7 like activity (**D**) was quantified via real-time analysis. Data displayed as mean ± SEM, *N* = 3. MDA-MB-231 cells were treated with Tg alone (0.5 μM) or in combination with indicated concentrations of Amgen 44 (AMG44) for 72 h after which (**E**) PERK, P-eIF2α and eIF2α was assessed via immunoblotting while (**F**) cell death was quantified via real-time analysis of Cytotox Red uptake. Data shown as mean ± SEM, *N* = 3. Statistical significance for live-cell analysis was determined using two-way ANOVA followed by TUKEY HSD post-hoc analysis. ***p ≤ 0.001.

Once activated, PERK propagates downstream signaling by phosphorylating Serine 51 on eIF2α. Since ablation of IRE1-XBP1s signaling lowered PERK protein expression in MDA-MB-231 cells, we assessed whether this reduction diminished PERK signaling. Analysis of eIF2α demonstrated rapid eIF2α phosphorylation in scrambled control cells as early as 6 h Tg (Fig. 8A). However, this was reduced in XBP1 KO cells implying a decrease in PERK signaling (Fig. 8A). These results indicate that shut down of IRE1-XBP1s mediated signaling reduces PERK expression thus decreasing PERK mediated signaling. Given that sustained PERK activation has been associated with the onset of cell death, we questioned if IRE1 inhibition would influence ER stress-induced cell death. MDA-MB-231 cells were treated with Tg alone or a combination of Tg plus IRE1 inhibitor MKC8866 for up to 72 h. Tg treatment induced activation of both the PERK and IRE1 branches of the UPR as demonstrated by appearance of XBP1s alongside an upshift in PERK expression and increase in P-eIF2α (Fig. 8B). Addition of MKC8866 reduced Tg-induced XBP1s and lowered PERK expression and downstream signaling (Fig. 8B). Analysis of pro-caspase-3 indicated a reduction in pro-caspase-3 in Tg treated samples which was partially suppressed in Tg plus MKC8866 treated cells (Fig. 8B). Assessment of cleaved caspase-3 also indicated a reduction in cleaved p17 caspase-3 in those cells treated with a combination of Tg plus MKC8866 suggesting a suppression in ER-stress induced apoptosis (Fig. 8B). To complement this, real-time live cell imaging was used to assess cell death. Those cells treated with Tg alone displayed a clear increase in Cytotox Red positivity indicative of cell death starting at 36 h and continuing to increase thereafter. Combination of Tg with MKC8866 reduced Cytotox Red uptake indicating a slowdown in cell death kinetics (Fig. 8C). Analysis of caspase-3/7 like activity revealed a similar outcome with Tg treatment alone triggering an increase in caspase-3/7 like activity which was lowered upon combination with MKC8866 (Fig. 8D). To assess if a similar reduction in cell death could be observed by directly inhibiting PERK, we again employed the PERK inhibitor Amgen 44. Concentrations of Amgen 44 (2 μM) which completely inhibited PERK, accelerated Tg induced cell death (Fig. 8F). However, lower concentrations of Amgen 44, which modeled partial inhibition of PERK signaling (Fig. 8E) similar to MKC8866 treatment, reduced Tg-induced cell death indicating that partial inhibition of PERK signaling may slow the kinetics of ER stress-induced cell death (Fig. 8F).

## Discussion

IRE1, PERK and ATF6, while complementary, are largely considered parallel, but distinct signaling pathways. Whether this assumption accurately reflects reality is a question unexplored within the field. To date, most studies have focused upon the downstream pathways controlled by IRE1, PERK and ATF6, asking how collectively these signaling mechanisms resolve ER stress or initiate cell death. Consequently, our understanding of the key events important in dictating UPR outcomes is comprehensive. However, the same is not true of the subtle alterations that help to fine tune the UPR. In this study, we observed exposure to ER stress-initiated increases in IRE1, PERK and ATF6 suggesting IRE1, PERK and ATF6 expression, rather than being static, is dynamically regulated. By increasing the expression of ER stress sensors, expansion of UPR signaling networks is supported, increasing their capacity to adapt to excessive or prolonged periods of ER stress. Given the complementary nature of UPR signaling networks, we questioned if ER stress sensors had the capacity to regulate each other’s expression, helping to shape the duration and amplitude of UPR signaling.

The concept of ER stress sensor cross talk, as a means to fine-tune expression of ER stress sensors and hence UPR signaling, has largely been overlooked. With the exception of a study by Tsuru et al. in 2016, which reported genetic ablation of PERK or ATF4 lowered IRE1 expression and activity in Tm treated mouse fibroblasts [19] few studies have addressed this question. In our system, we demonstrated that blockade of PERK signaling, achieved via addition of the PERK inhibitor Amgen 44, reduced IRE1 expression in Tg treated MDA-MB-231 cells confirming a relationship between PERK and IRE1 as previously reported by Tsuru and colleagues. In addition, we expanded upon previous findings by demonstrating the existence of reciprocal cross talk between IRE1 and PERK, with IRE1 signaling promoting PERK protein expression during ER stress. Irrespective of the cell line tested, mechanism of inhibition, or stimulus inducing ER stress, interfering with IRE1 signaling decreased PERK protein expression particularly at later time points indicative of chronic ER stress. These results highlight a regulatory loop between IRE1 and PERK in which IRE1 helps to sustain PERK expression during chronic ER stress. Our findings point towards a transcriptional based regulation, with IRE1-XBP1s signaling rather than IRE1-RIDD signaling facilitating increases in PERK transcript. This is further underscored by results obtained using inducible XBP1s MDA-MB-231 cells where selective expression of XBP1s, in the absence of exogenous ER stress, increased PERK transcript and protein. Evidence supporting a relationship between XBP1s and PERK is available within the wider literature, with reduced PERK transcript reported in RNA-seq data sets derived from cells with impeded IRE1-XBP1s signaling [23, 24]. Given that XBP1s is a transcription factor, the most likely mechanism of PERK regulation is via binding of XBP1s to the PERK promoter. Analysis of previously published XBP1s chromatin immunoprecipitation (ChIP) sequencing data sets, supports PERK as a direct XBP1s target [23, 25, 26]. We confirmed this in our system, with ChIP-qPCR demonstrating binding of XBP1s to the PERK promoter region upon induction of ER stress. A relationship between IRE1-XBP1s signaling and PERK expression is also supported by recent work in colorectal cancer cells, which demonstrated inducible expression of XBP1s, amplified Tg induced PERK expression and downstream signaling [27]. These observations, together with the comprehensive analysis we have carried out in this study, indicate an amplification loop in which IRE1-XBP1s signaling helps to sustain PERK expression. Our findings suggest that this amplification may play an important role in maintaining PERK expression during long term chronic ER stress, as it is at these later time points that we observe the greatest depletion in PERK expression upon IRE1 inhibition.

As cells transition from an acute to a chronic ER stress, UPR signaling shifts from a pro-survival to a pro-death response. Given that blockade of IRE1-XBP1s signaling reduced PERK expression and downstream signaling, we speculated that this might also impede the kinetics of ER stress induced cell death. Assessment of cell death via Cytotox Red uptake confirmed this prediction with MDA-MB-231 cells treated with a Tg MKC8866 combination displaying reduced cell death and lower effector caspase activation when compared to those treated with Tg alone. To complement these findings, we demonstrated a similar reduction in Tg induced cell death could be mimicked by modeling partial inhibition of PERK signaling achieved by low concentrations of the PERK inhibitor Amgen 44. These results suggest that by sustaining PERK expression, IRE1-XBP1s signaling may help promote the transition from pro-survival to pro-death signaling. In agreement with these findings, Spaan and colleagues reported XBP1s mediated increases in PERK expression in LS174T colorectal cells, correlated with an increased sensitivity to Tg induced cell death [27].

Collectively, our findings make a compelling case for cross talk between IRE1 and PERK, with IRE1-XBP1s signaling helping to sustain PERK expression during chronic ER stress. While intriguing from a fundamental aspect, this observation may also have implications in the use of UPR targeted therapeutics. Small molecule inhibitors of IRE1, PERK and ATF6 are available with several, particularly those targeting IRE1, showing a significant therapeutic benefit in preclinical models of cancer [16]. Both IRE1 RNase and kinase targeting inhibitors have shown efficacy in models of triple negative breast cancer as standalone agents and when used in combination with chemotherapeutics [28–30]. Indeed, the IRE1 inhibitor ORIN1001 (previously known as MKC8866) is in phase 2 clinical trials for advanced breast cancer [31]. Since our results, using multiple IRE1 inhibitors including MKC8866, demonstrate a reduction in PERK signaling upon sustained ER stress, it indicates that IRE1 inhibitors may also have a capacity to lessen PERK expression and signaling. This observation is especially interesting when we consider sustained PERK signaling is reported as critical for tumor survival under hypoxic conditions [32, 33]. While pharmacological inducers of ER stress were predominantly used in this study, we did observe IRE1 mediated regulation of PERK expression in response to serum deprivation, a stress frequently experienced in vivo by cancer cells. This observation suggests IRE1-PERK cross talk is functional under conditions of physiological ER stress. However, further studies are necessary to explore the impact of this crosstalk within cancer models. Whether IRE1 inhibitors have the added advantage of reducing PERK signaling within an in vivo setting remains an intriguing question.

In conclusion, our study demonstrates the existence of cross talk between the IRE1 and PERK branches of the UPR, with IRE1-XBP1s signaling helping to sustain PERK expression during long term, chronic ER stress. These findings, alongside prior published literature, suggest that IRE1, PERK and ATF6 signaling branches of the UPR should not be considered as distinct parallel entities but rather a closely intertwined signaling network.

### Supplementary information

Supplemental Data

Uncropped blots

## Data Availability

The data supporting the conclusions of this article are included in the article, its supplementary files or available from the corresponding author under reasonable request.
