# Supplementary material for: IRE1 signaling increases PERK expression during chronic ER stress
Source: Cell Death Dis. 2024 Apr 18;15(4):276. doi: 10.1038/s41419-024-06663-0 (PMC11026449; doi:10.1038/s41419-024-06663-0)
Supplement: Supplementary file 1 — Supplemental Data [file 41419_2024_6663_MOESM1_ESM.docx]

**Supplementary Data**

**Supplementary Figure 1: Suppression of PERK signaling reduces IRE1 expression during ER stress.** MDA-MB-231 cells were treated for the indicated times with 0.5 μM Tg alone or in combination with Amgen 44 (AMG44, 2 µM) after which cells where harvested and cell lysates were analysed via immunoblotting for IRE1, XBP1s, PERK, P-eIF2α and total eIF2α. Actin was used as a loading control. Blots are representative of N=3. Arrow denotes XBP1s band, * indicates non-specific band(s),

**Supplementary Figure 2: XBP1 KO MDA-MB-231 cells lack IRE1-XBP1s signaling but retain IRE1-RIDD signaling.** Scrambled control (SCBL) and XBP1 knockout (XBP1-KO) MDA-MB-231 cells were treated with 0.5 μM Tg alone or in combination with MKC8866 (20 μM) for 18 h after which RNA was extracted and relative expression changes in *DGAT2* expression were assessed by qPCR. Mean relative expression ± SD, reference gene *GAPDH*, N=3. Statistical significance for all qPCR experiments was determined using one-way ANOVA followed by TUKEY HSD post-hoc analysis. [***] p ≤ 0.001.

**Table S1.  List of primer sequences.**

| Primer | Gene | Sequence |
| --- | --- | --- |
| Human ERN1 Forward | IRE1 (human) | GGG ATT TTT GGA AGT ACC AG |
| Human ERN1 Reverse | IRE1 (human) | AAA GTC CAT TTG ATT GAG CC |
| Human EIF2AK3 Forward | PERK (human) | AAG TGG AAT TTC AGT GTT GG |
| Human EIF2AK3 Reverse | PERK (human) | AGG TGC TTT CAA TAA ATC CG |
| Human ATF6 Forward | ATF6 (human) | AAT ATA TGC TAG GGT TAG AGG C |
| Human ATF6 Reverse | ATF6 (human) | TTC TCT GAC ACA ACT TCA TC |
| Human XBP1s Forward | XBP1s (human) | GCT GAG TCC GCA GCA GGT |
| Human XBP1s Reverse | XBP1s (human) | CTG GGT CCA AGT TGT CCA GAA T |
| Human GAPDH Forward | GAPDH (human) | TCG GAG TCA ACG GAT TTG |
| Human GAPDH Reverse | GAPDH (human) | CAA CAA TAT CCA CTT TAC CAG AG |
| Human RPL10 Forward | RPL10 (human) | GCC AAG TTA TCA TGT CCA TC |
| Human RPL10 Reverse | RPL10 (human) | TTG AGA TGT GGA TCT TCT GG |
| Human EIF2AK3 Promoter Forward | PERK (human) | TCA GAA TCC GCC ACG TAG TAA G |
| Human EIF2AK3 Promoter Reverse | PERK (human) | TGG AAG TGG ATG TCC CTC AAA G |
| Mouse EIF2AK3 Forward | PERK (mouse) | CTT AAT CCA TTC TCC TTC TAG G |
| Mouse EIF2AK3 Reverse | PERK (mouse) | TAG TAT GGC AGA TAG TAA CCG |
| Mouse XBP1s Forward | XBP1s (mouse) | GCT GAG TCC GCA GCA GGT |
| Mouse XBP1s Reverse | XBP1s (mouse) | CAG GGT CCA ACT TGT CCA GAA T |
| Mouse RPL13a Forward | RPL13a (mouse) | AGC CTA CCA GAA AGT TTG CTT AC |
| Mouse RPL13a Reverse | RPL13a (mouse) | GCT TCT TCT TCC GAT AGT GCA TC |

**Figure S1**

**
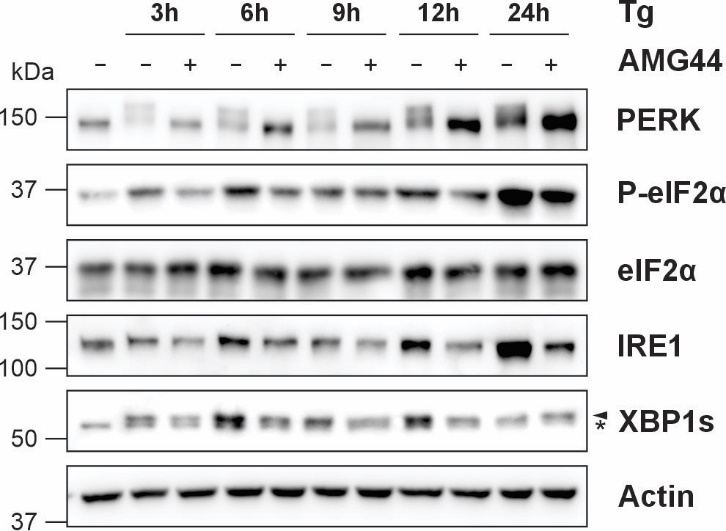
**

**Figure S2**

**
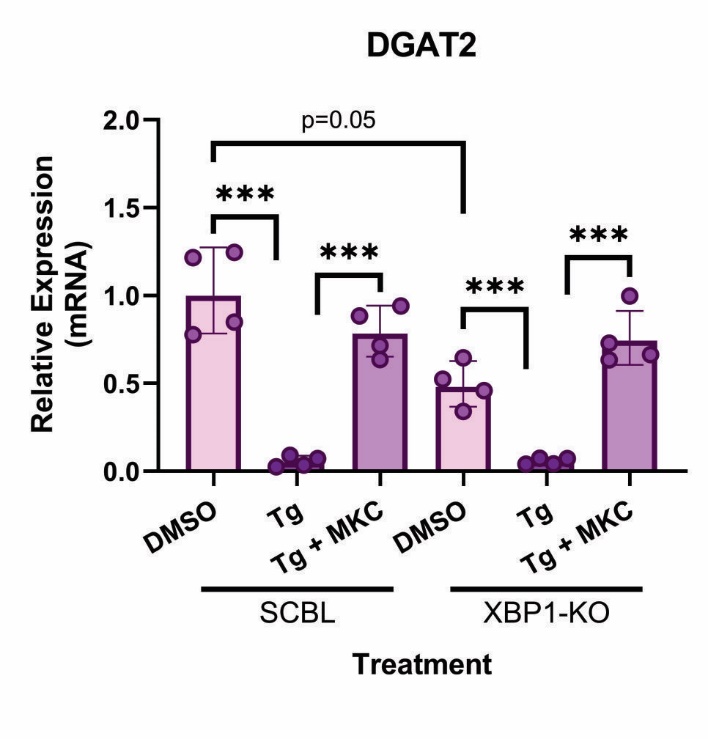
**
