# Supplementary material for: IRE1 signaling increases PERK expression during chronic ER stress
Source: Cell Death Dis. 2024 Apr 18;15(4):276. doi: 10.1038/s41419-024-06663-0 (PMC11026449; doi:10.1038/s41419-024-06663-0)

Full and uncropped Western Blot for Figure 1D (PERK)
Blots 1-6 are in the figure.

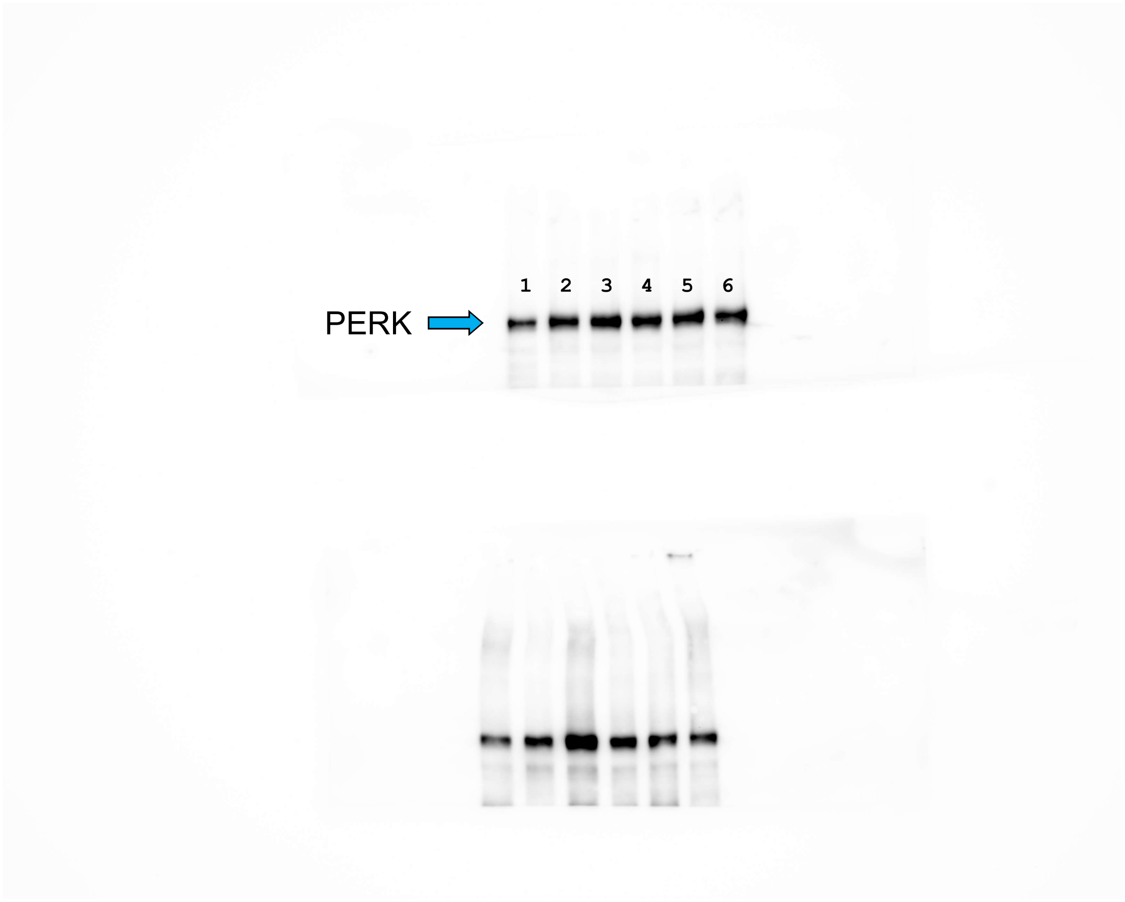

Full and uncropped Western Blot for Figure 1D (ATF4)
Blots 1-6 are in the figure.

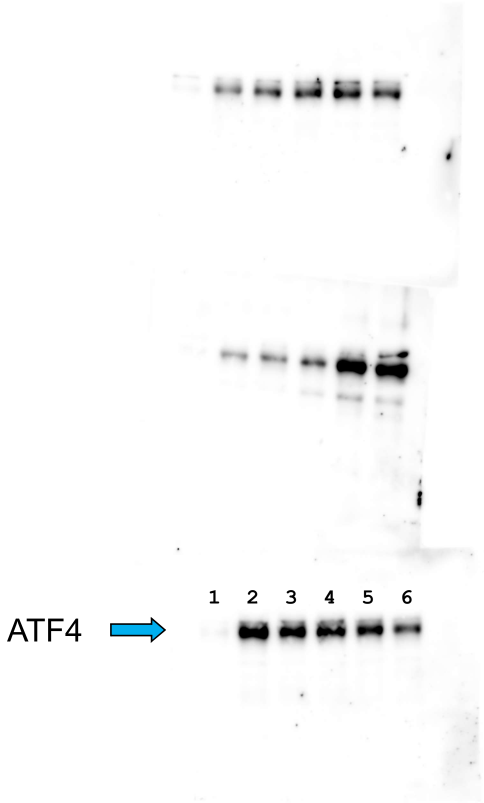

Full and uncropped Western Blot for Figure 1D (IRE1)

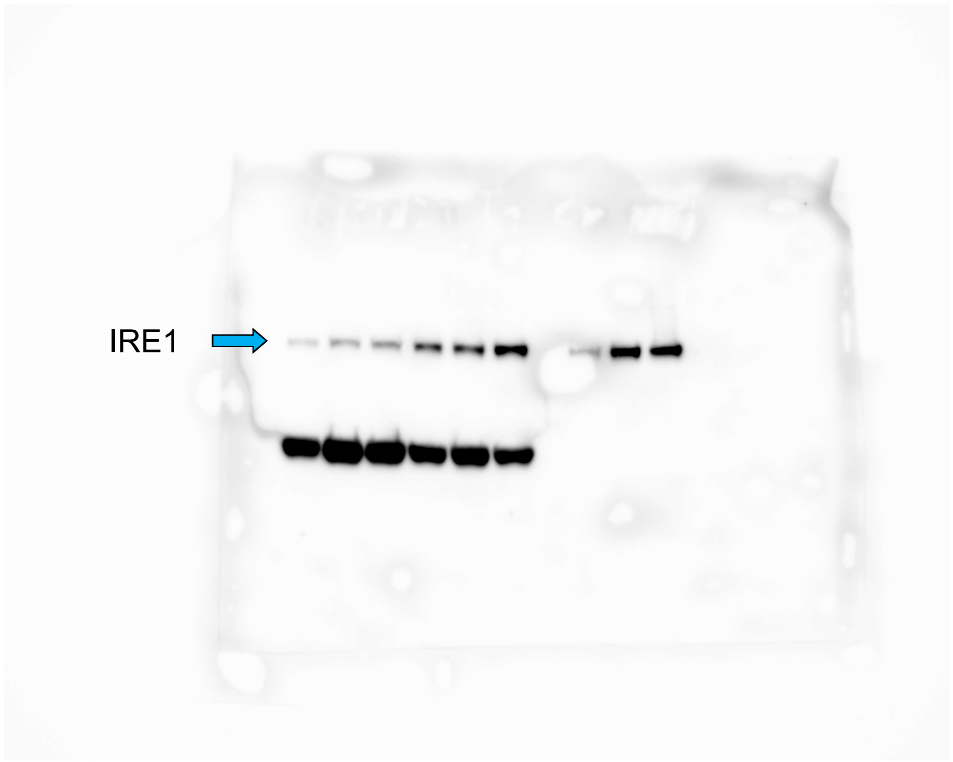

Full and uncropped Western Blot for Figure 1D (XBP1s)
Blots 1-6 are in the figure.

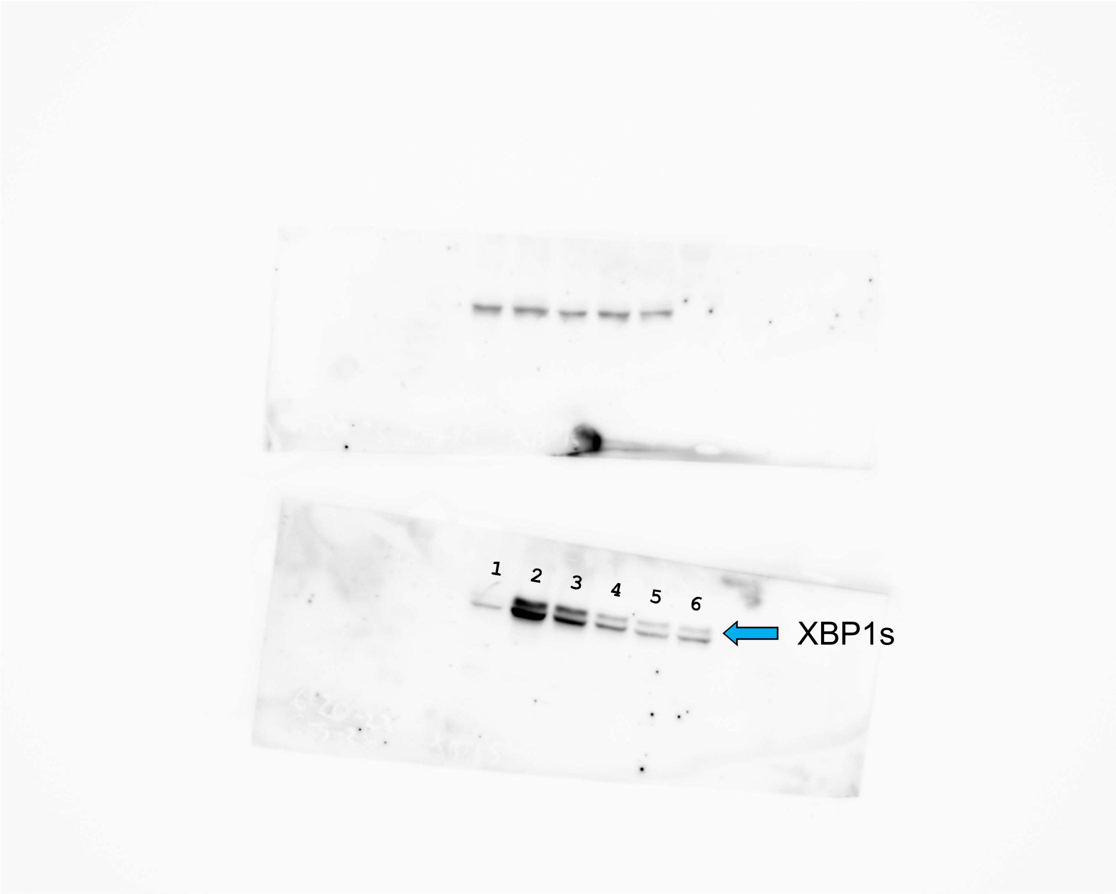

Full and uncropped Western Blot for Figure 1D (ATF6)

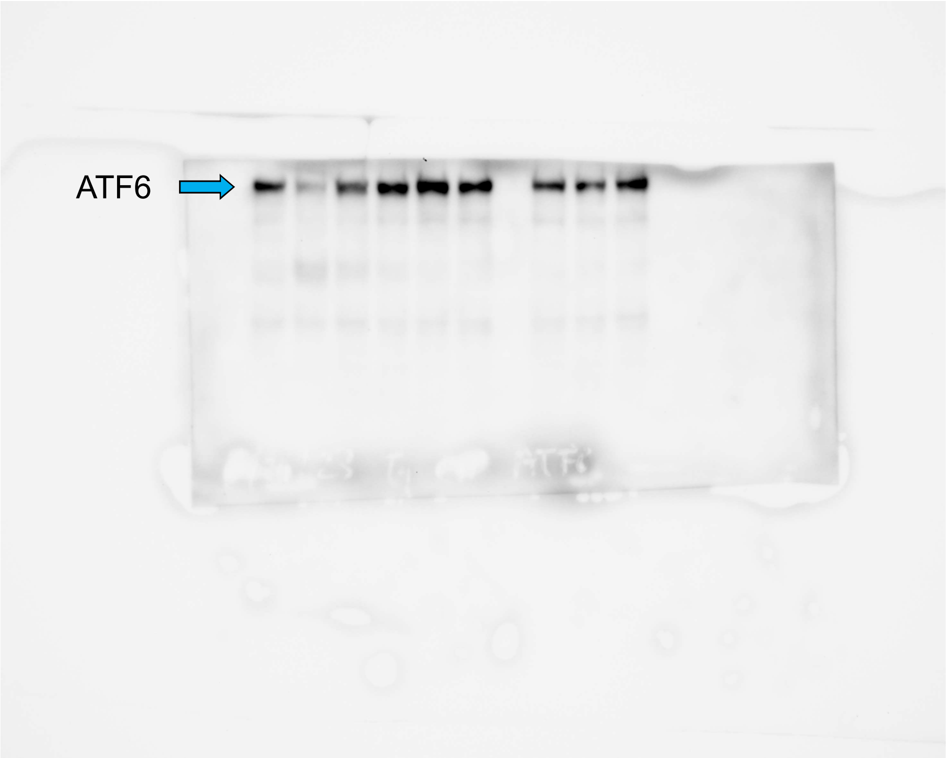

Full and uncropped Western Blot for Figure 1D (Actin)

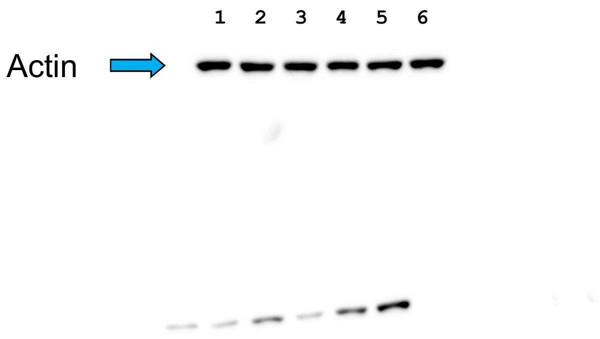

Full and uncropped Western Blot for Figure 2A (PERK)
Blots 1-4 are in the figure.

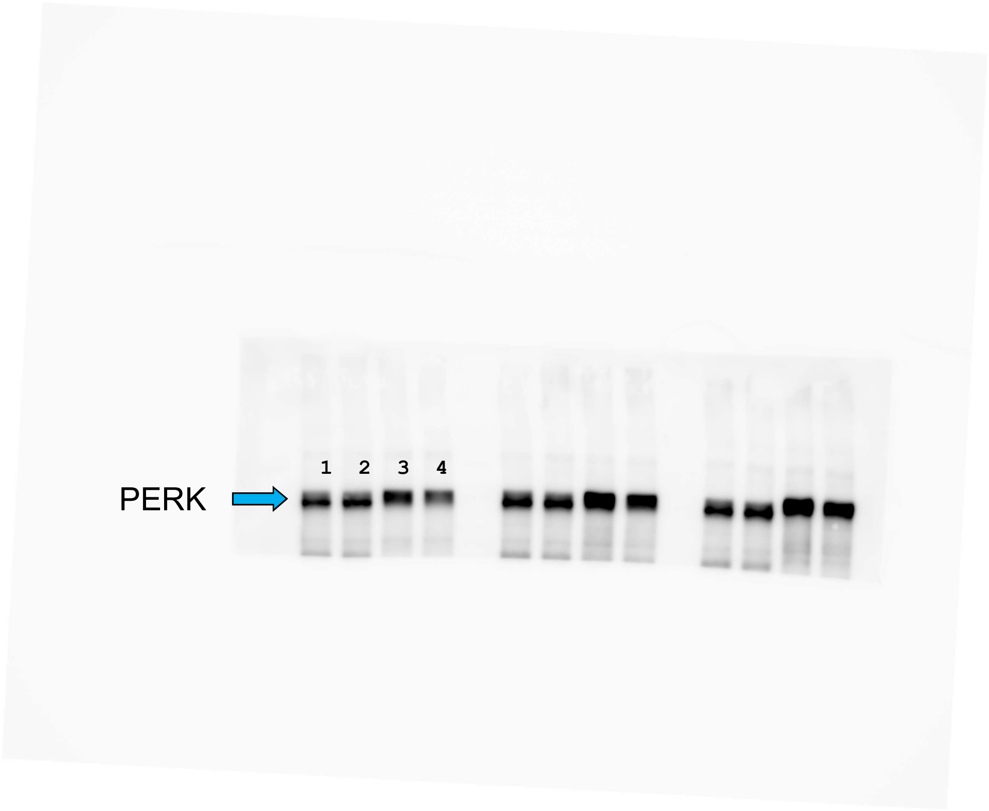

Full and uncropped Western Blot for Figure 2A (IRE1)
Blots 1-4 are in the figure.

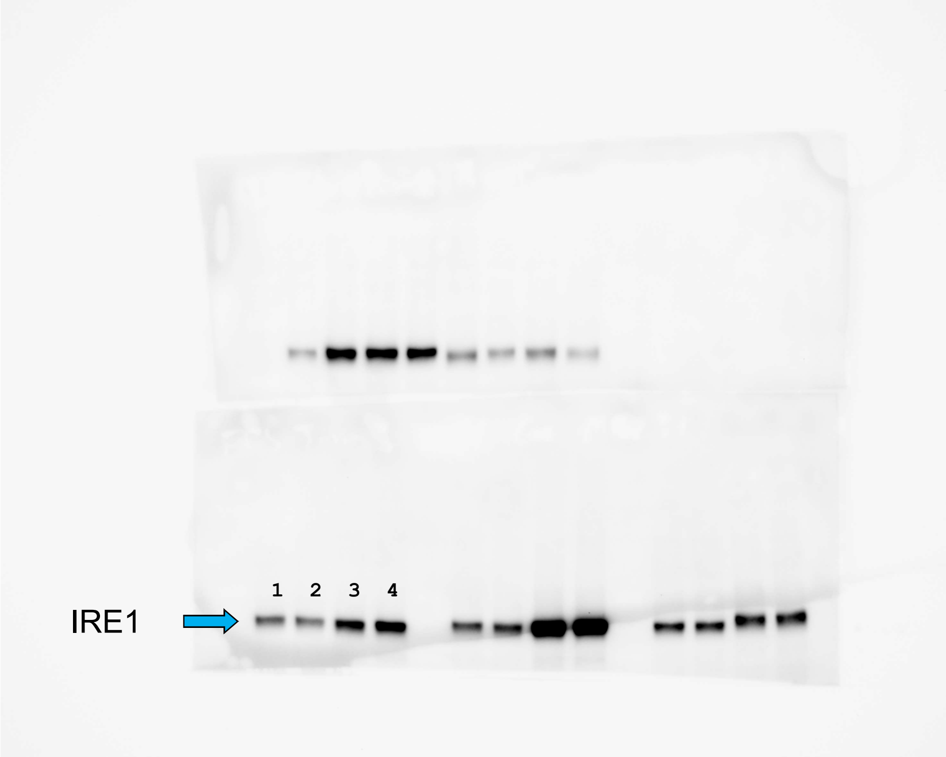

Full and uncropped Western Blot for Figure 2A (XBP1s)
Blots 1-4 are in the figure.

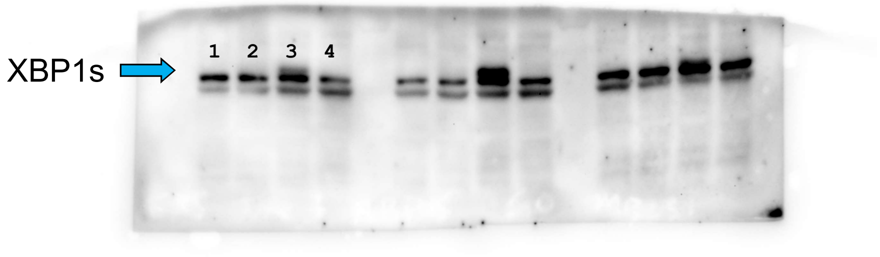

Full and uncropped Western Blot for Figure 2A (Actin)
Blots 1-4 are in the figure.

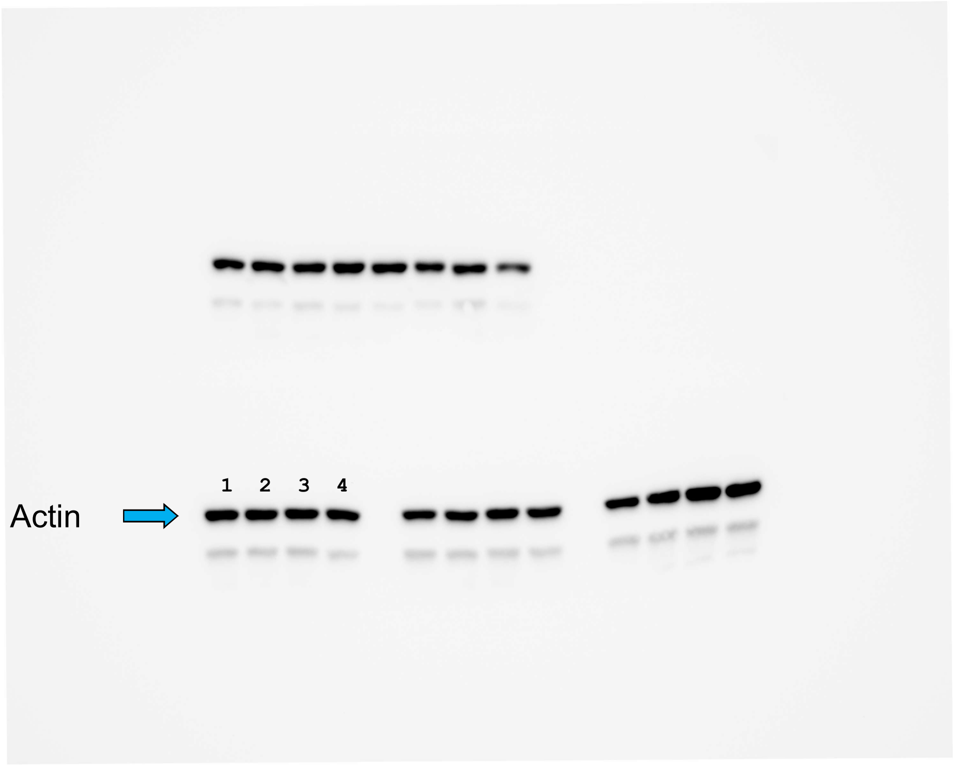

Full and uncropped Western Blot for Figure 2B (PERK)
Blots 1-4 are in the figure.

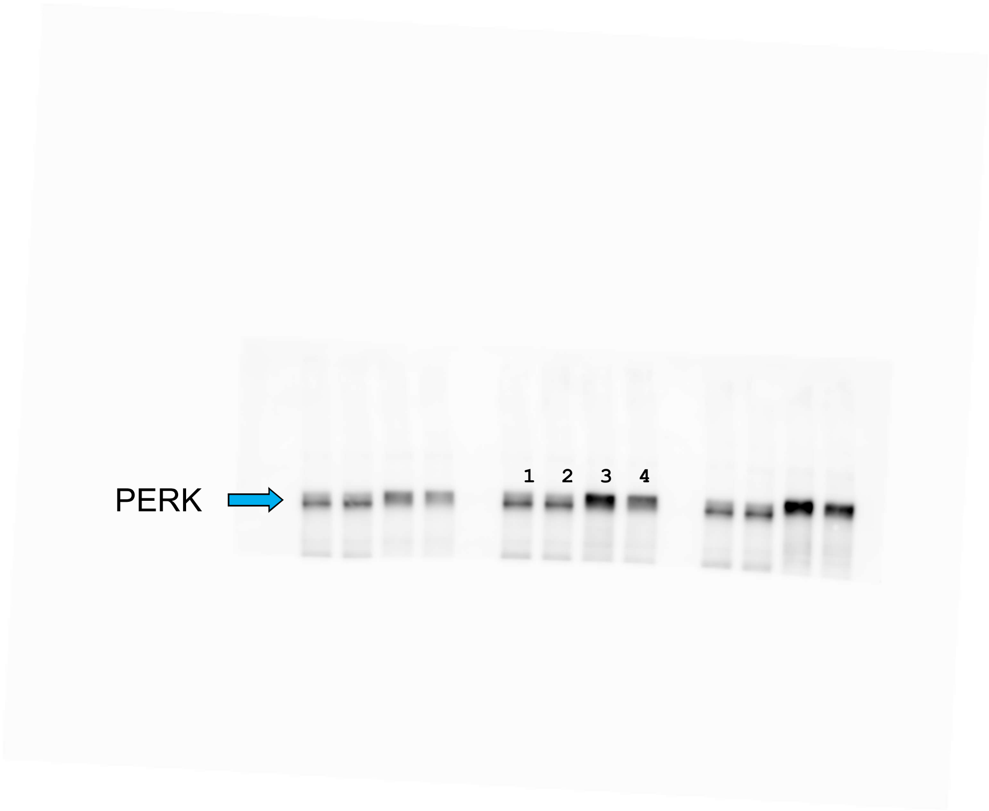

Full and uncropped Western Blot for Figure 2B (IRE1)
Blots 1-4 are in the figure.

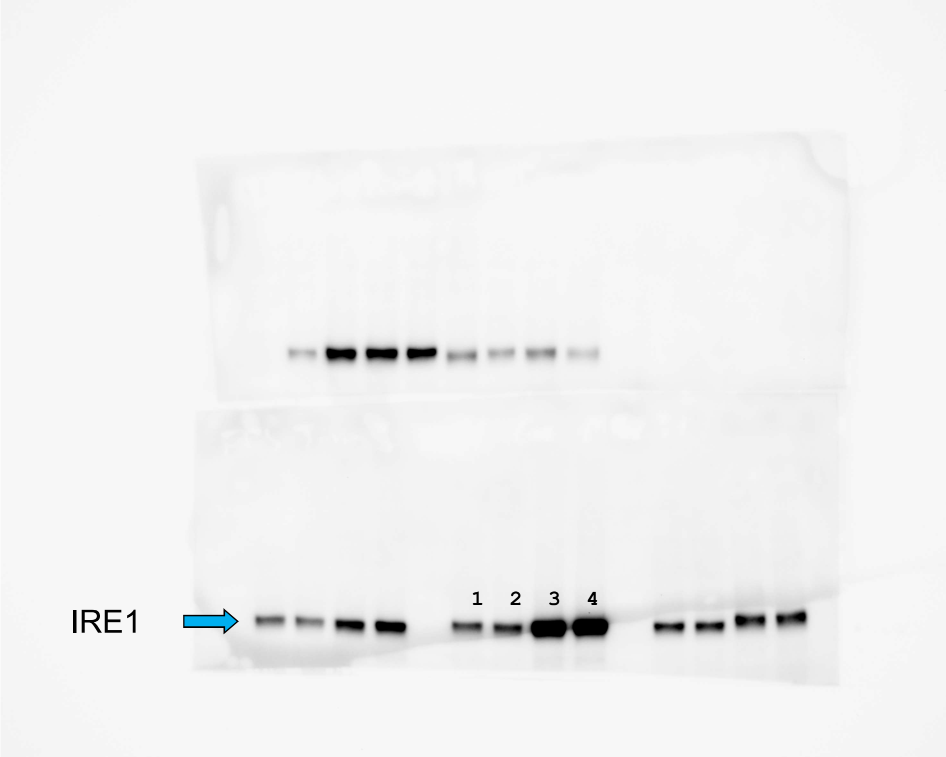

Full and uncropped Western Blot for Figure 2B (XBP1s)
Blots 1-4 are in the figure.

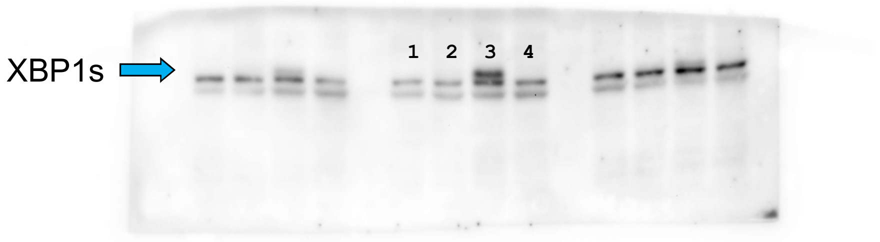

Full and uncropped Western Blot for Figure 2B (Actin)
Blots 1-4 are in the figure.

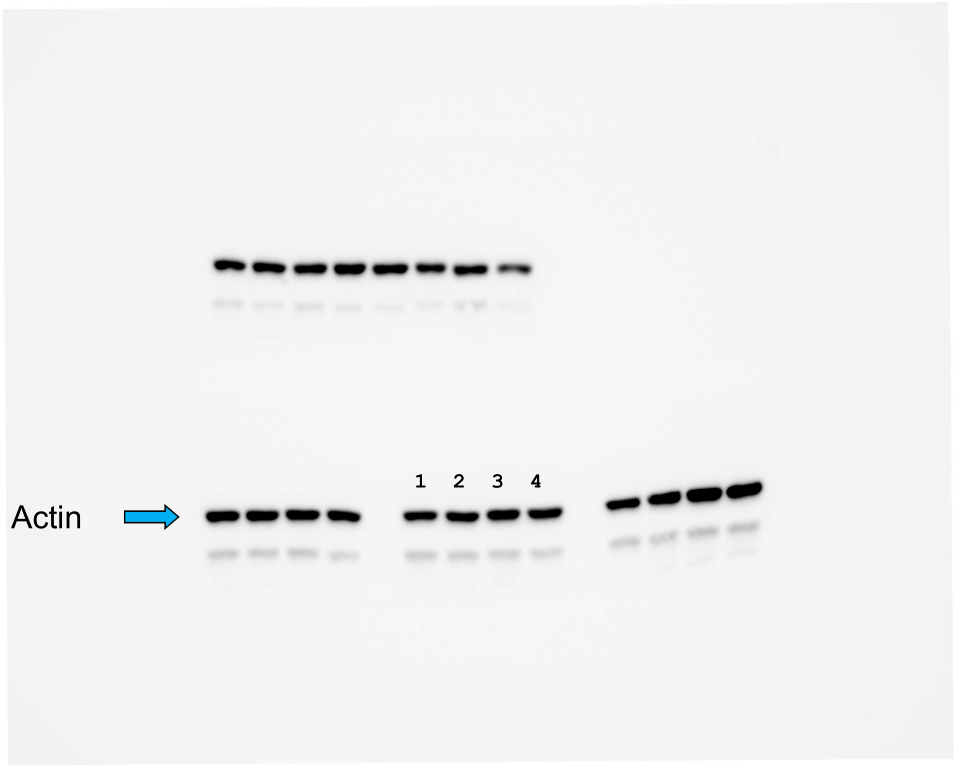

Full and uncropped Western Blot for Figure 2C (PERK)
Blots 1-4 are in the figure.

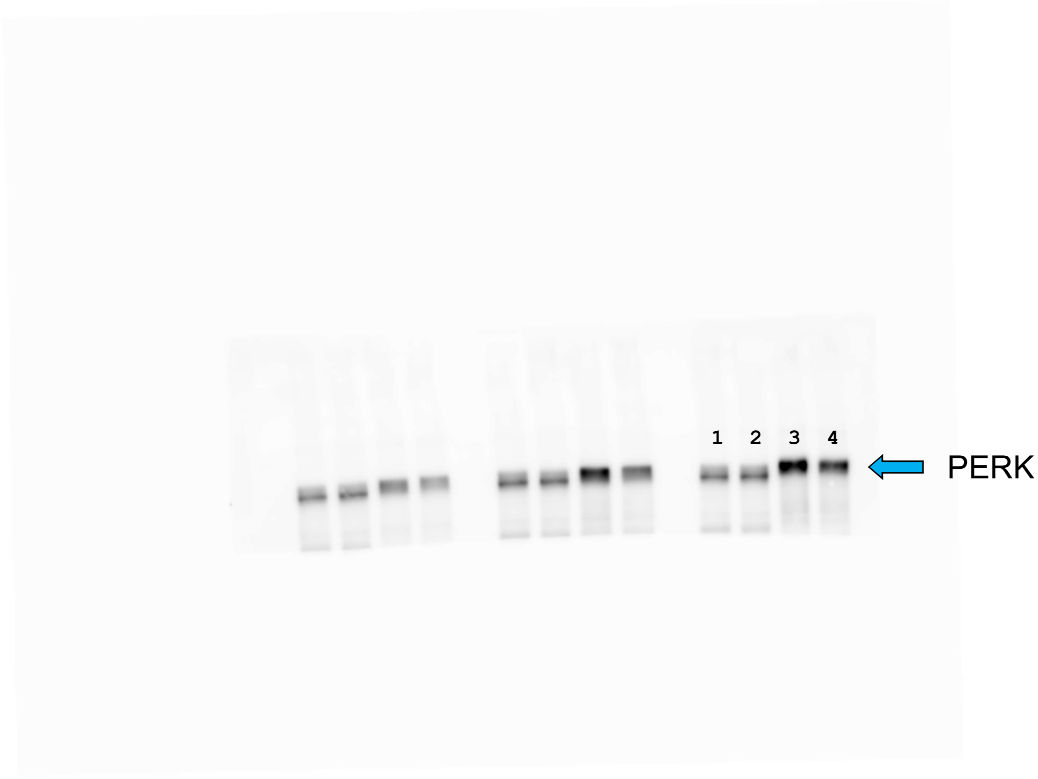

Full and uncropped Western Blot for Figure 2C (IRE1)
Blots 1-4 are in the figure.

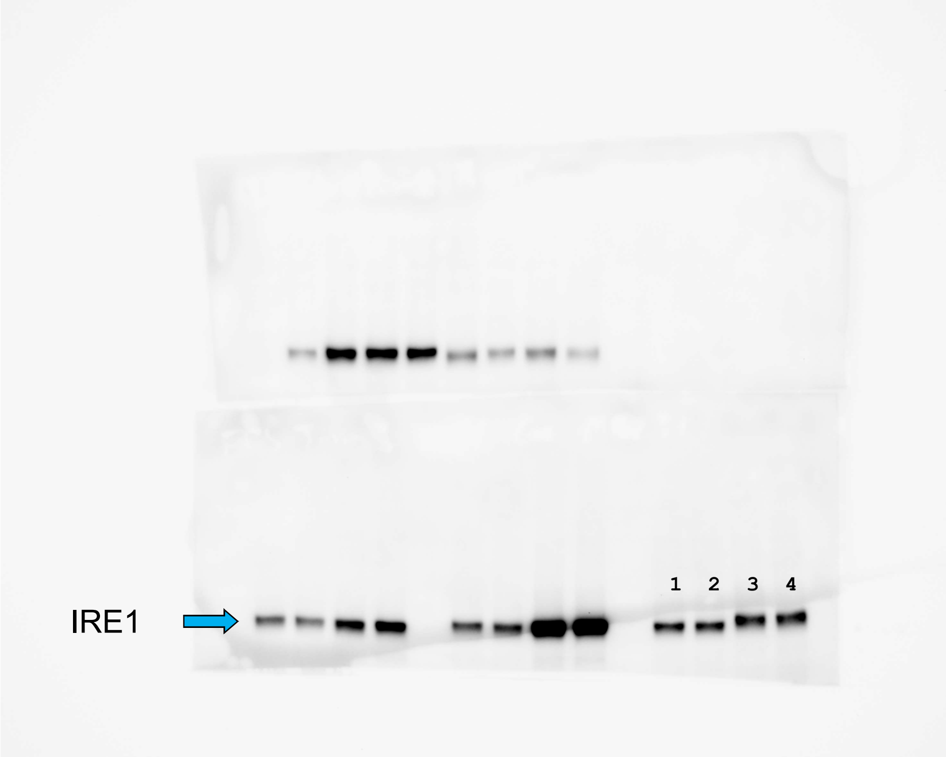

Full and uncropped Western Blot for Figure 2C (XBP1s)
Blots 1-4 are in the figure.

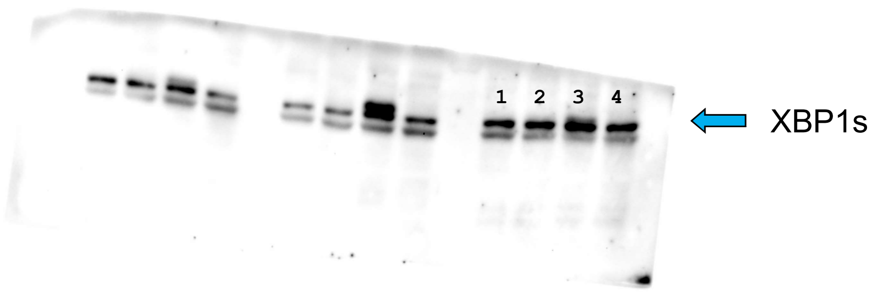

Full and uncropped Western Blot for Figure 2C (Actin)
Blots 1-4 are in the figure.

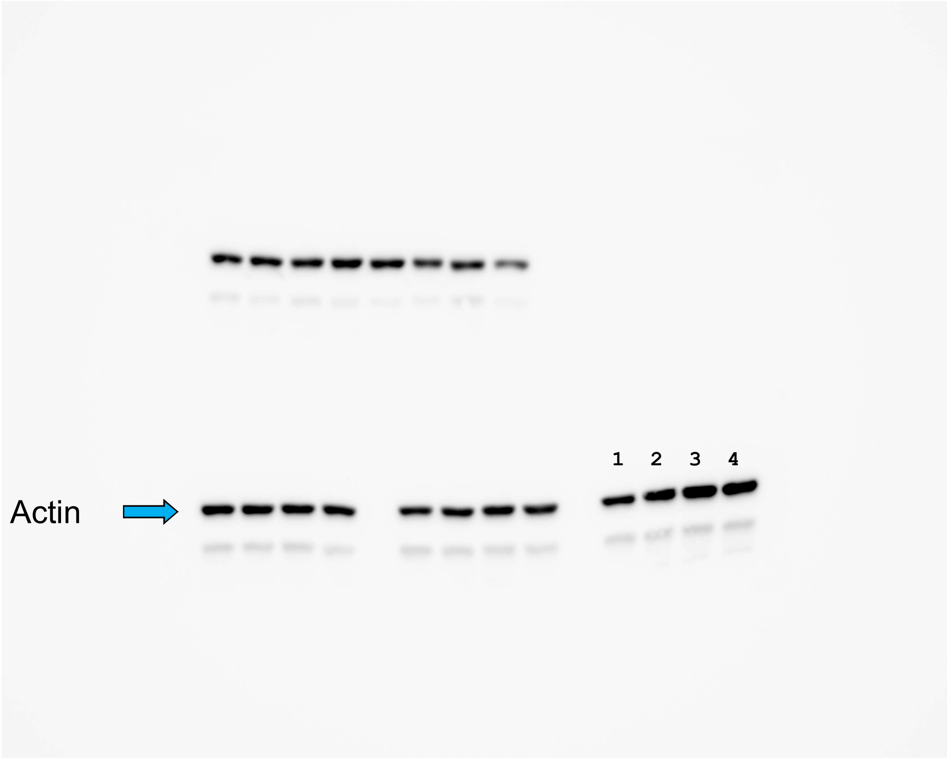

Full and uncropped Western Blot for Figure 2D (PERK)

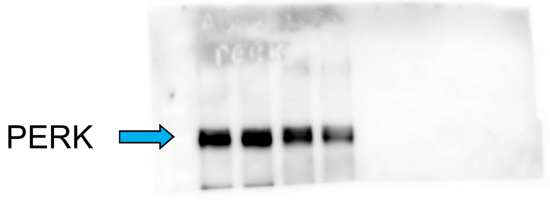

Full and uncropped Western Blot for Figure 2D (XBP1s)

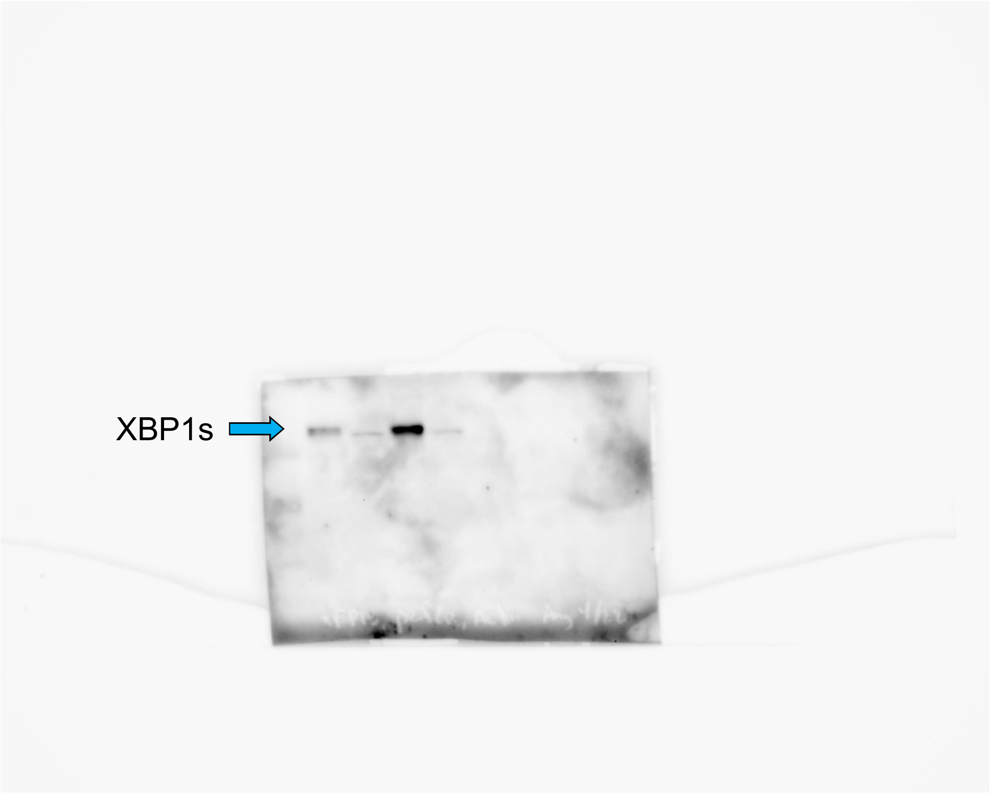

Full and uncropped Western Blot for Figure 2D (Actin)
Blots 1-4 are in the figure.

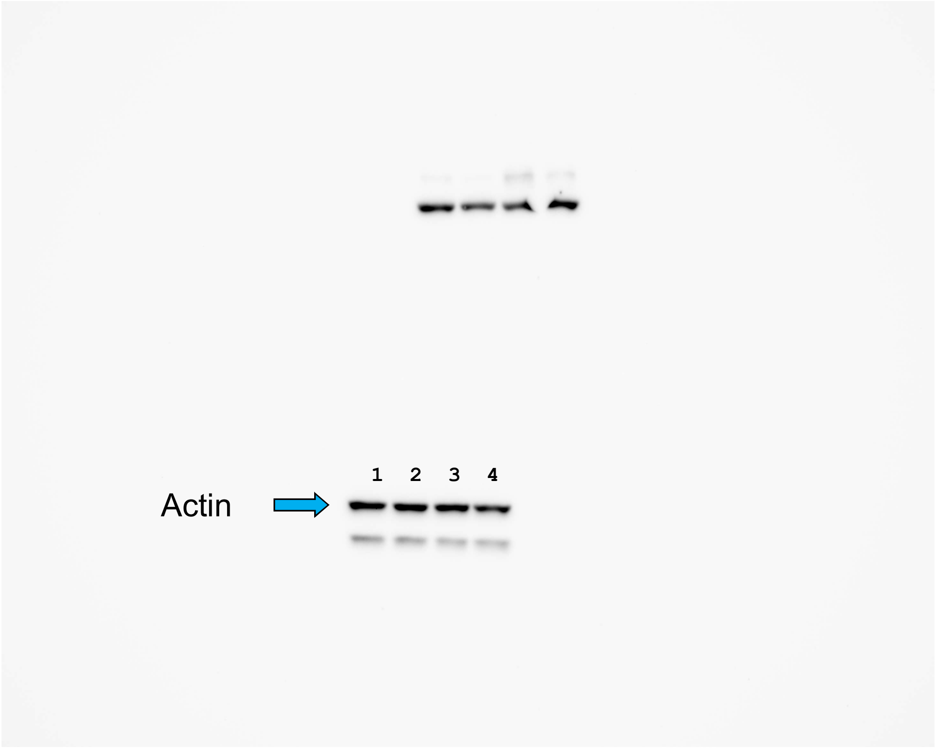

Full and uncropped Western Blot for Figure 2E (PERK)
Blots 1-12 are in the figure.

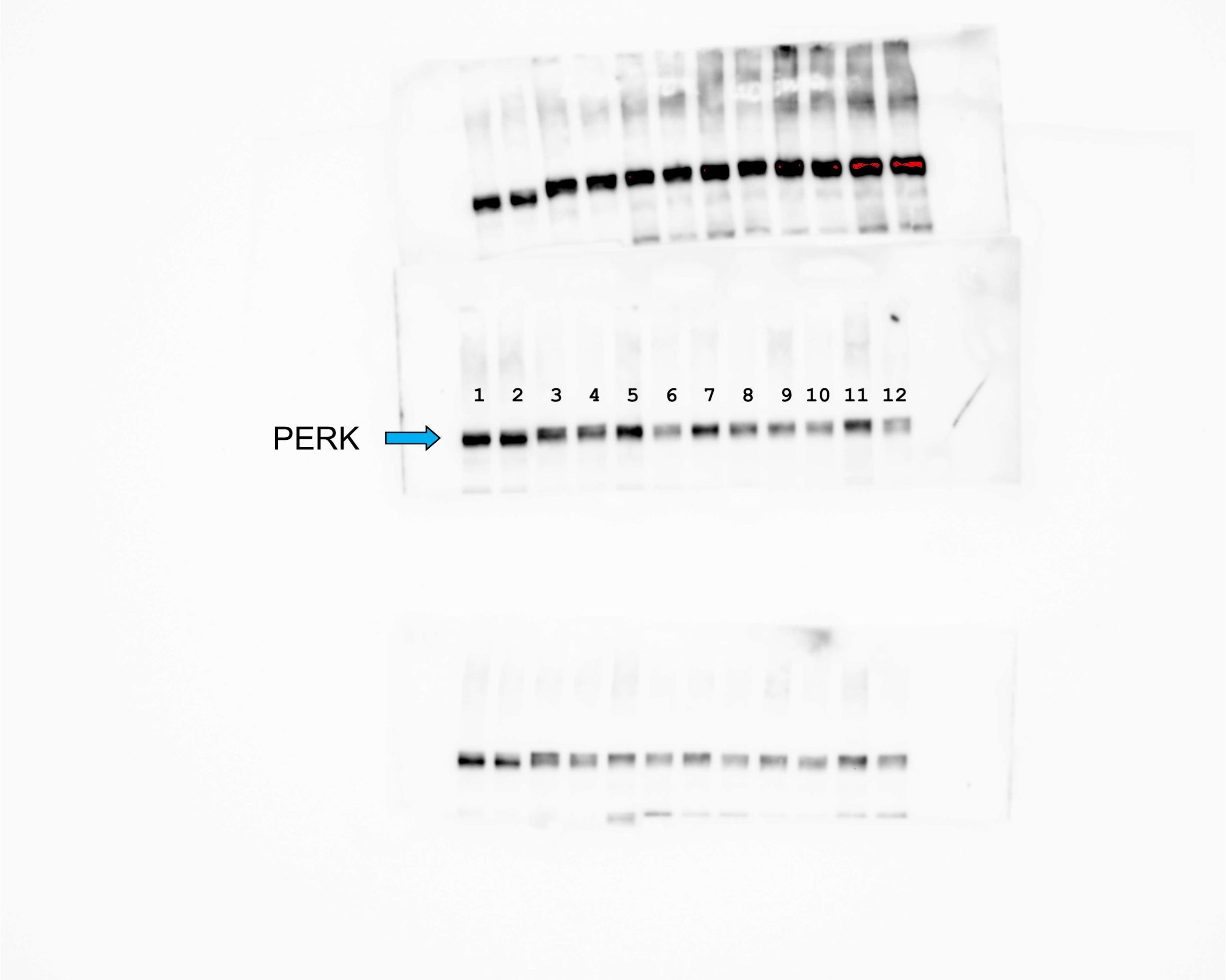

Full and uncropped Western Blot for Figure 2E (XBP1s)

Blots 1-12 are in the figure.

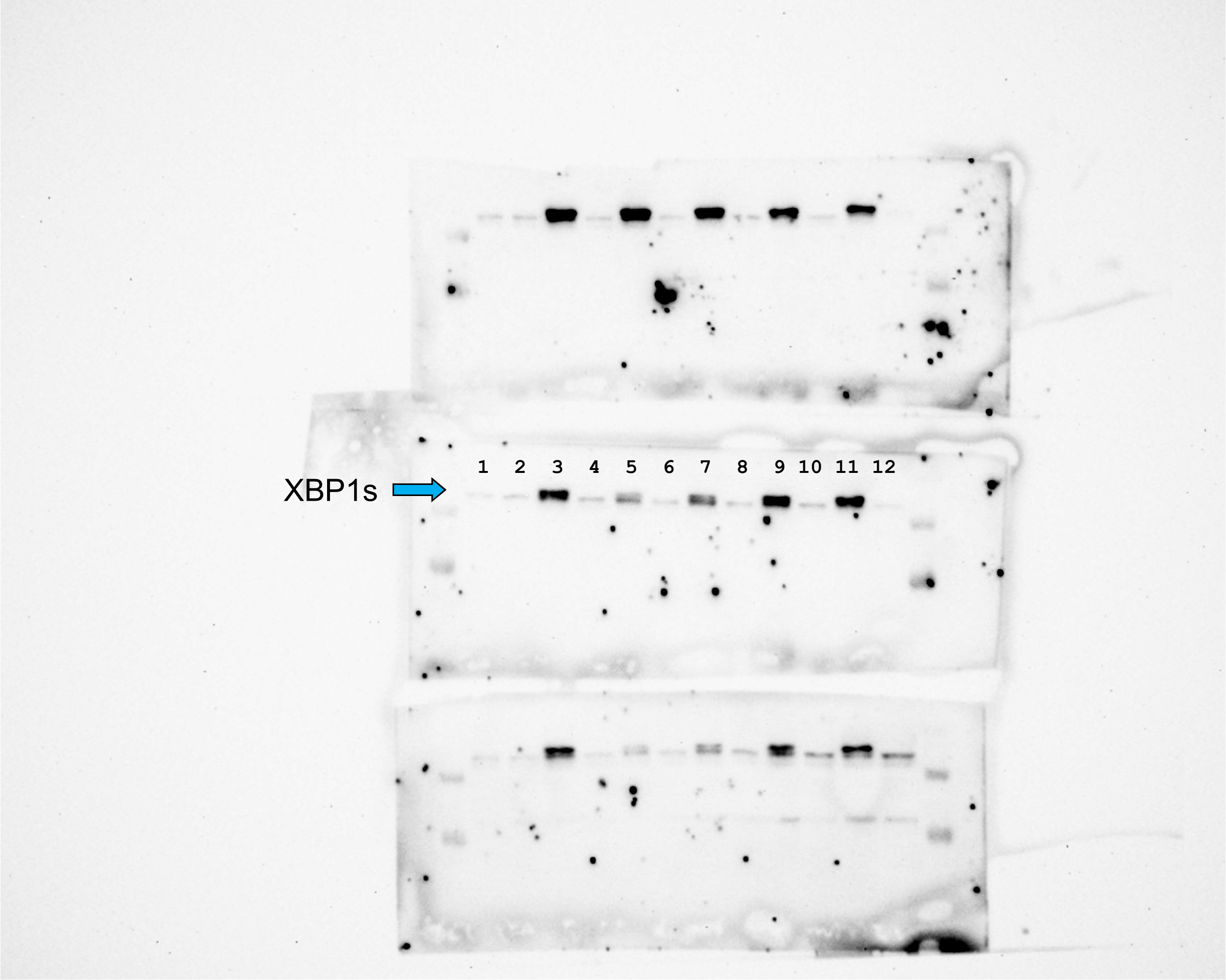

Full and uncropped Western Blot for Figure 2E (Actin)
Blots 1-12 are in the figure.

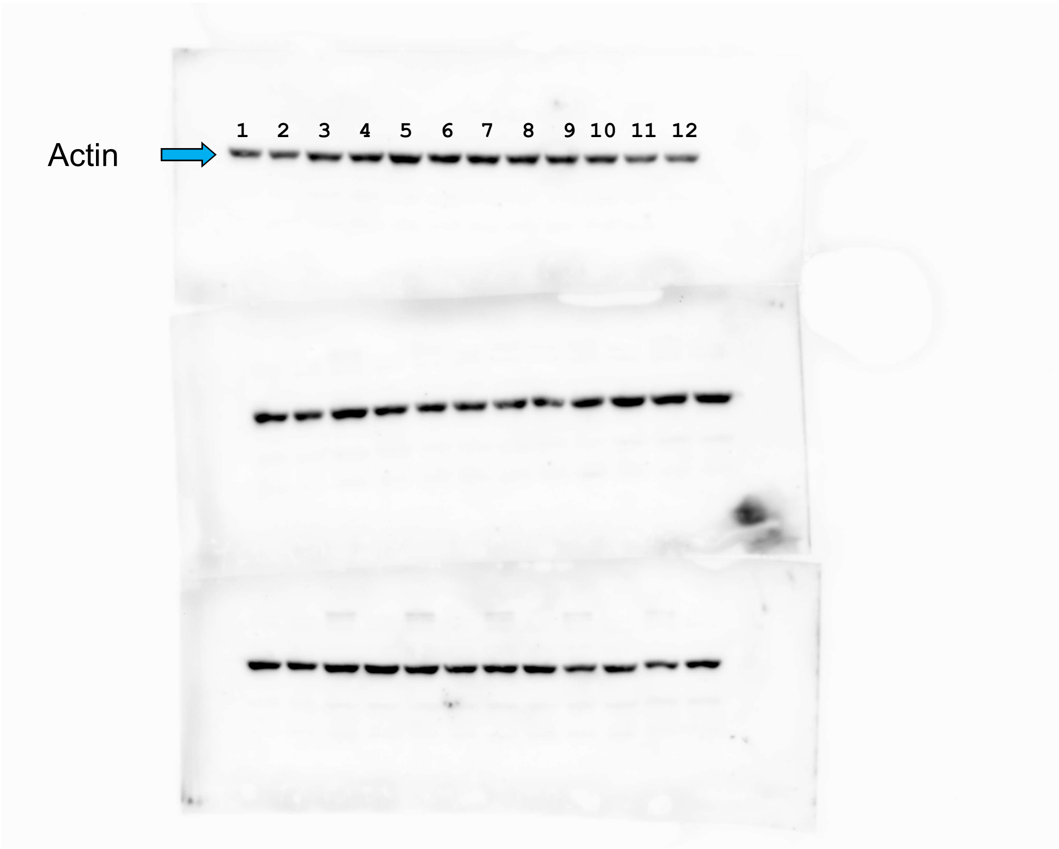

Full and uncropped Western Blot for Figure 2F (PERK)
Blots 1-12 are in the figure.

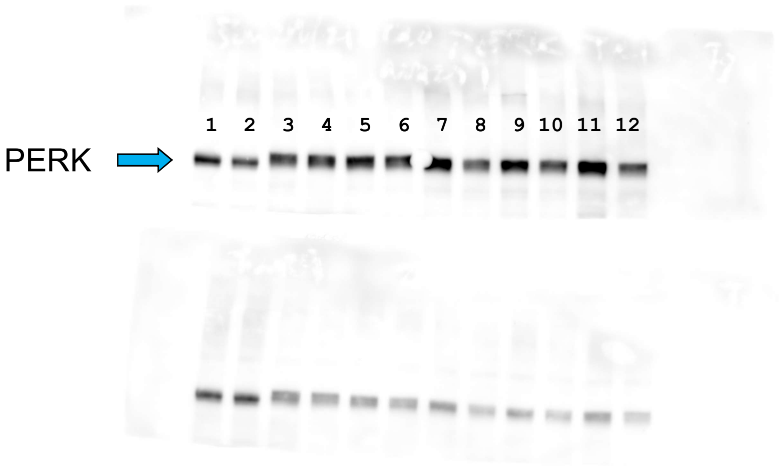

Full and uncropped Western Blot for Figure 2F (XBP1s)
Blots 1-12 are in the figure.

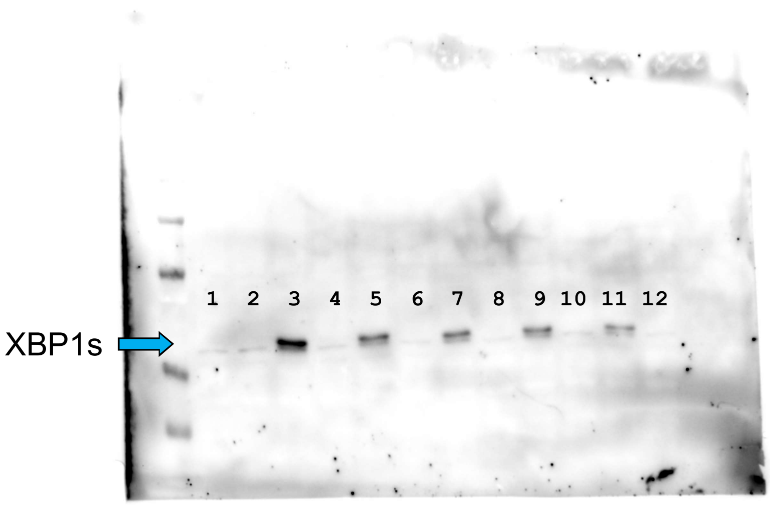

Full and uncropped Western Blot for Figure 2F (Actin)
Blots 1-12 are in the figure.

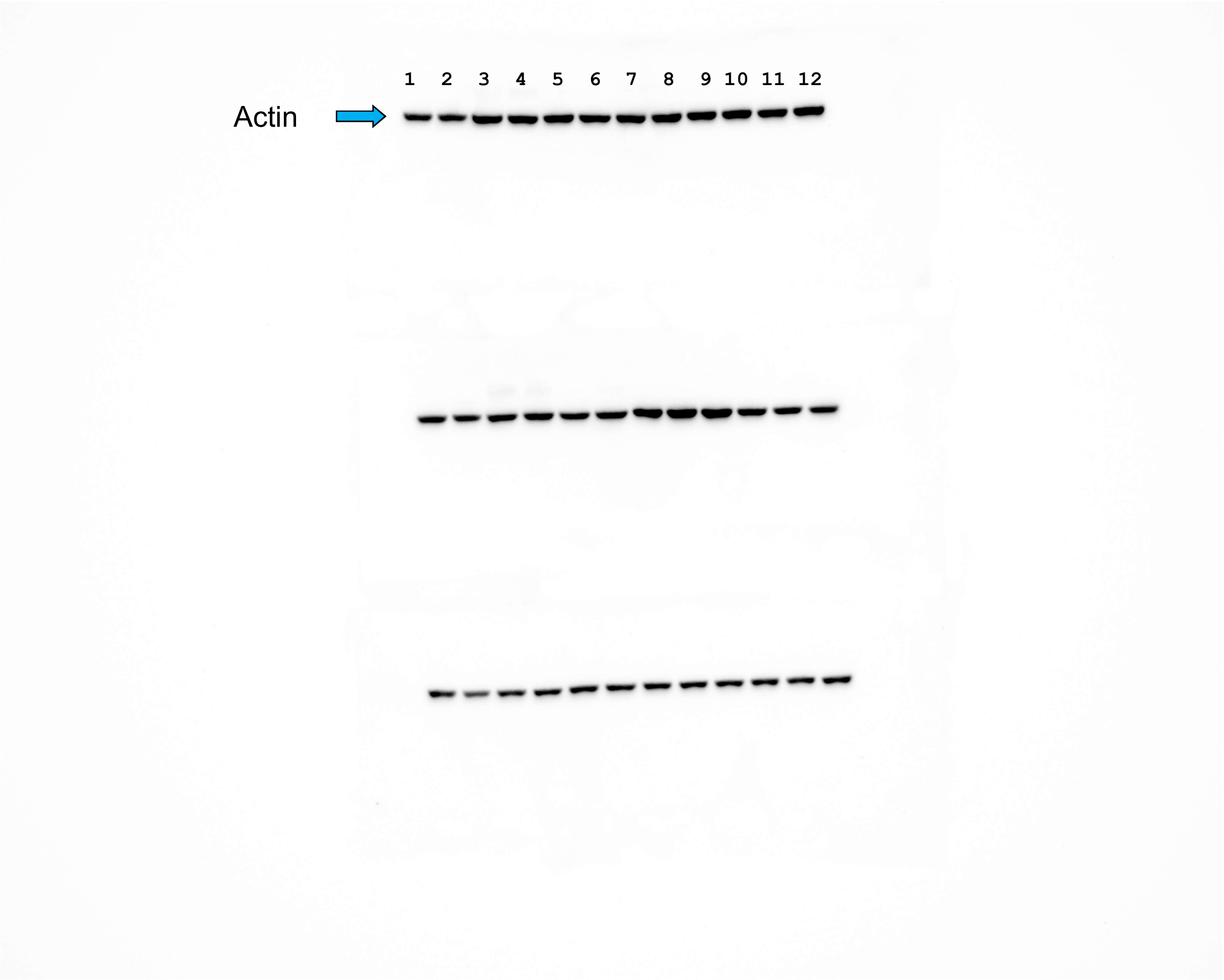

Full and uncropped Western Blot for Figure 2G (PERK)
Blots 1-12 are in the figure.

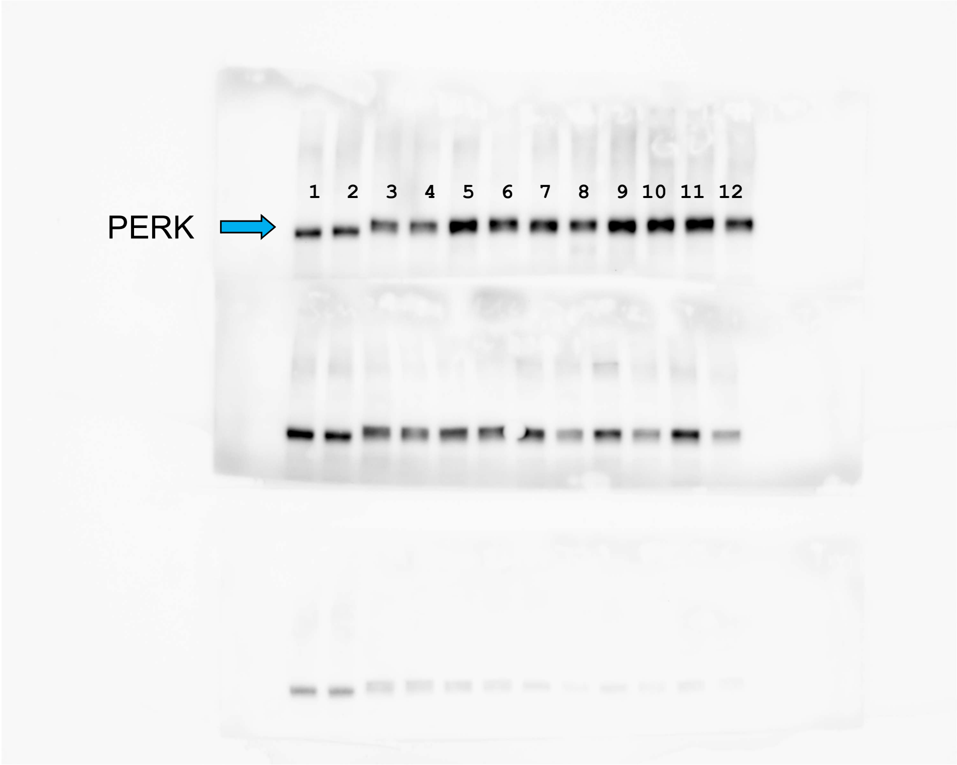

Full and uncropped Western Blot for Figure 2G (XBP1s)
Blots 1-12 are in the figure.

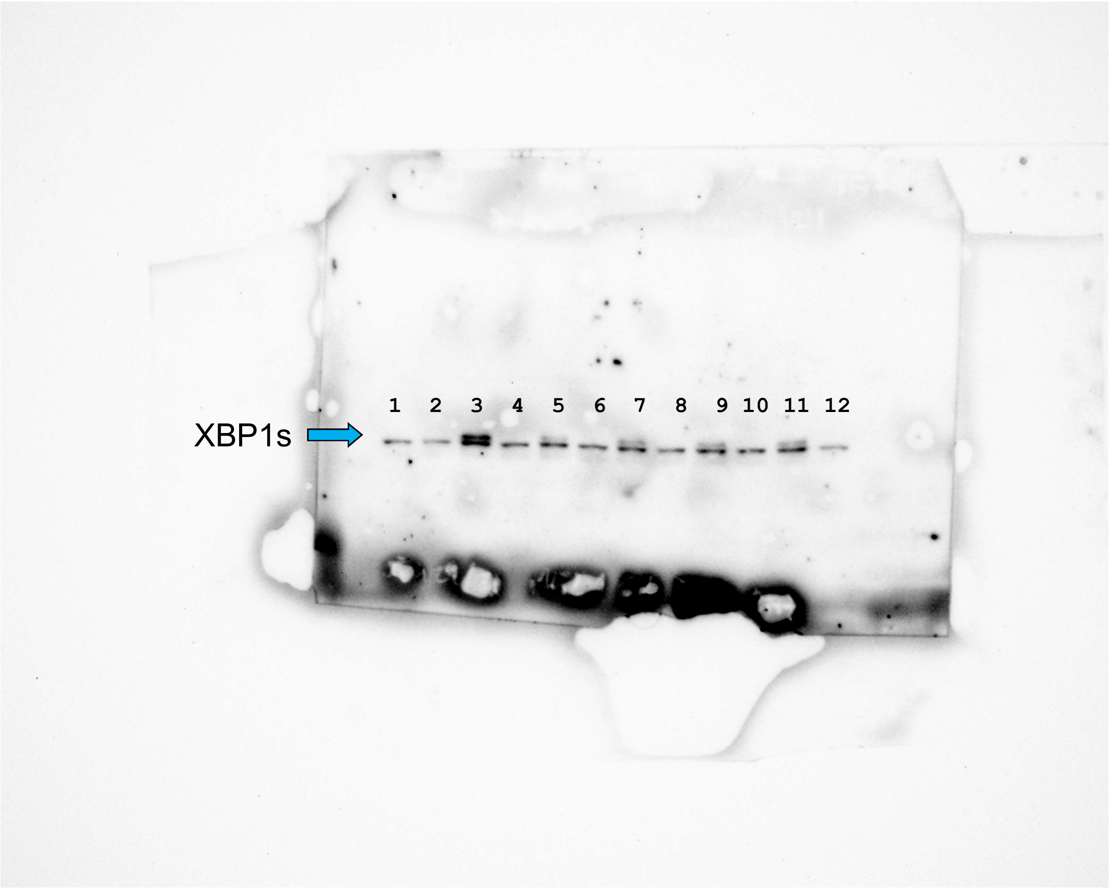

Full and uncropped Western Blot for Figure 2G (Actin)
Blots 1-12 are in the figure.

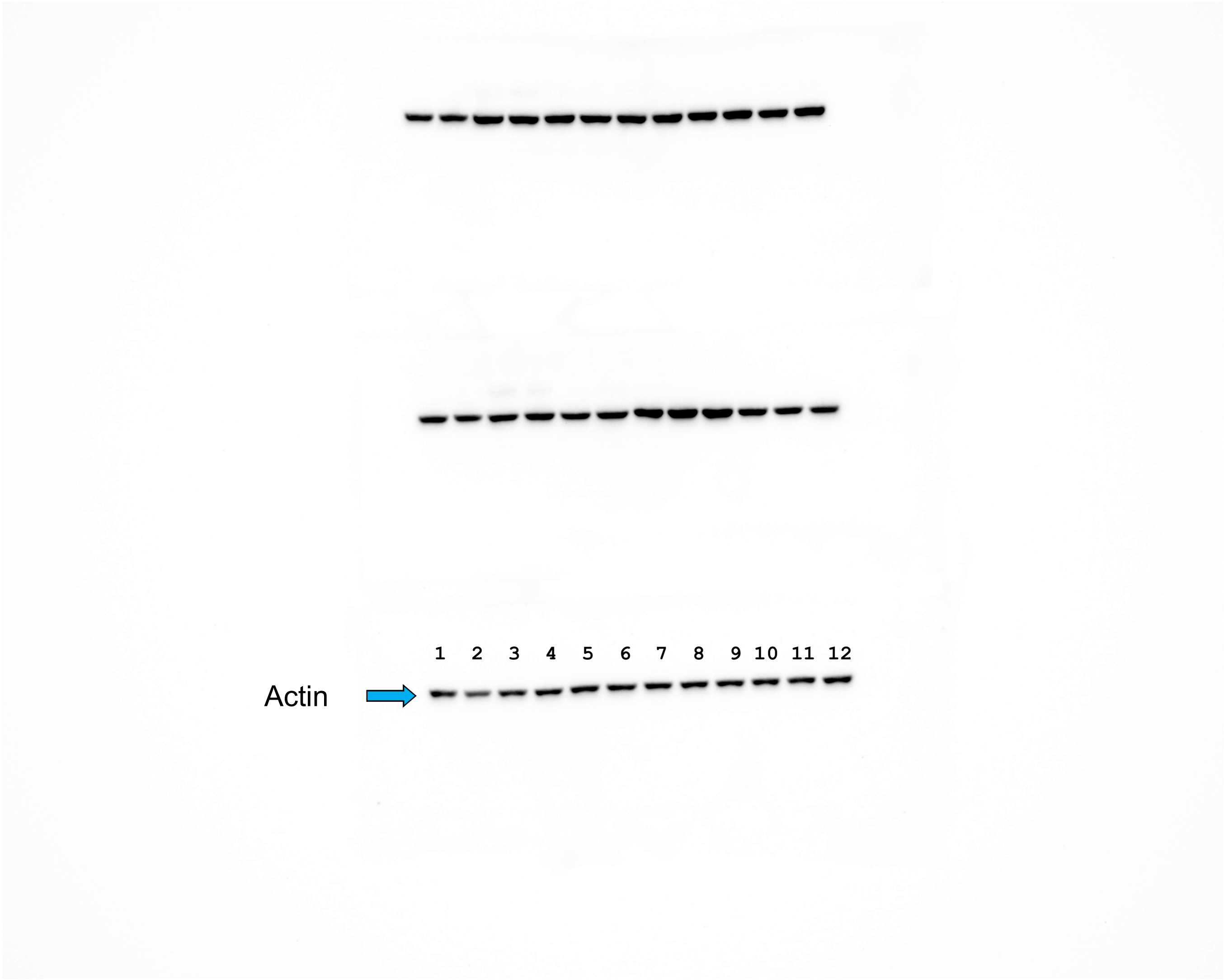

Full and uncropped Western Blot for Figure 2H (ATF6)
Blots 1-11 are in the figure.

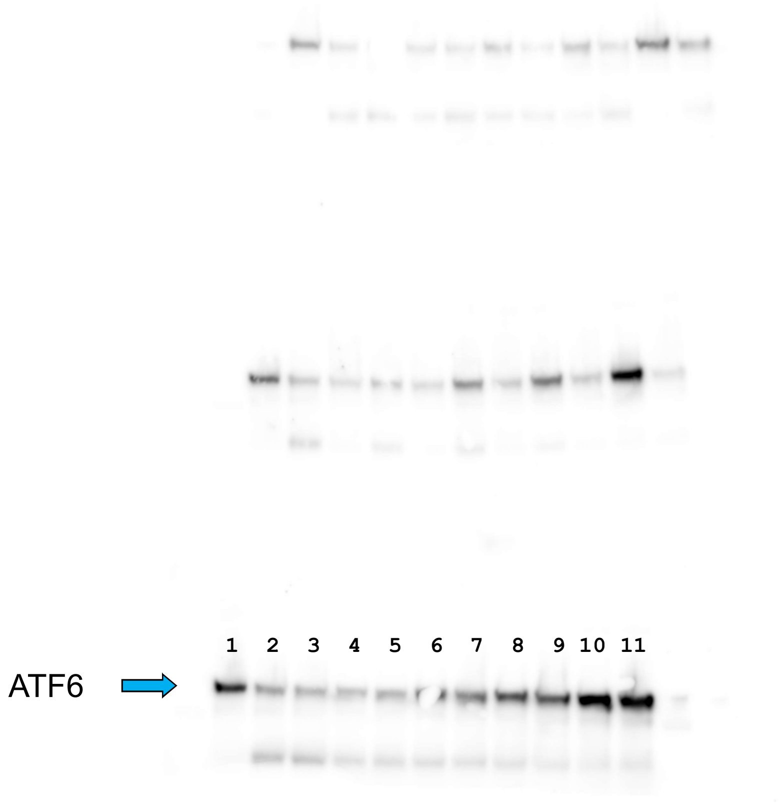

Full and uncropped Western Blot for Figure 2H (ATF6N)
Blots 1-11 are in the figure.

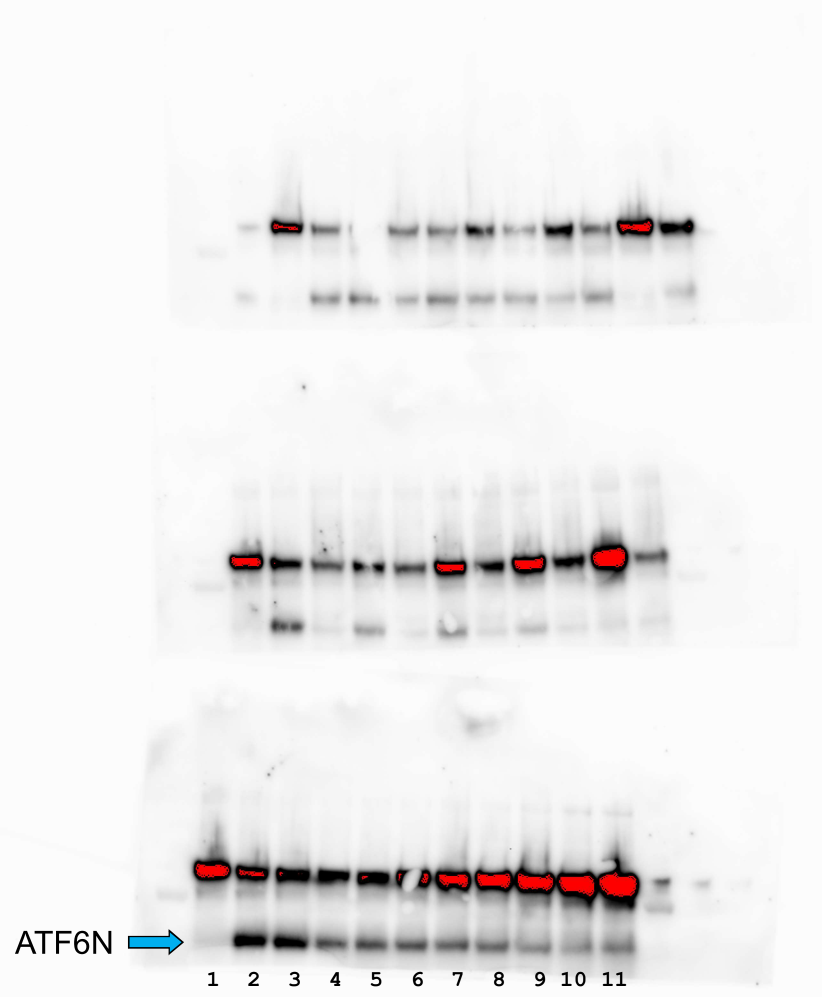

Full and uncropped Western Blot for Figure 2H (XBP1s)

Blots 1-11 are in the figure.

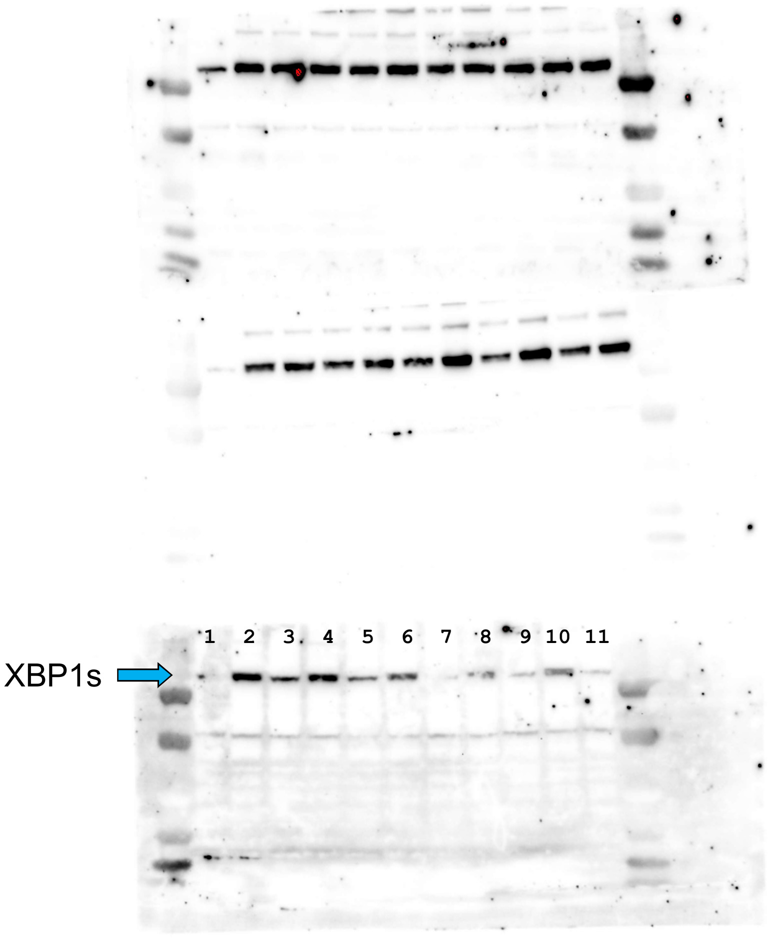

Full and uncropped Western Blot for Figure 2H (Actin)
Blots 1-11 are in the figure.

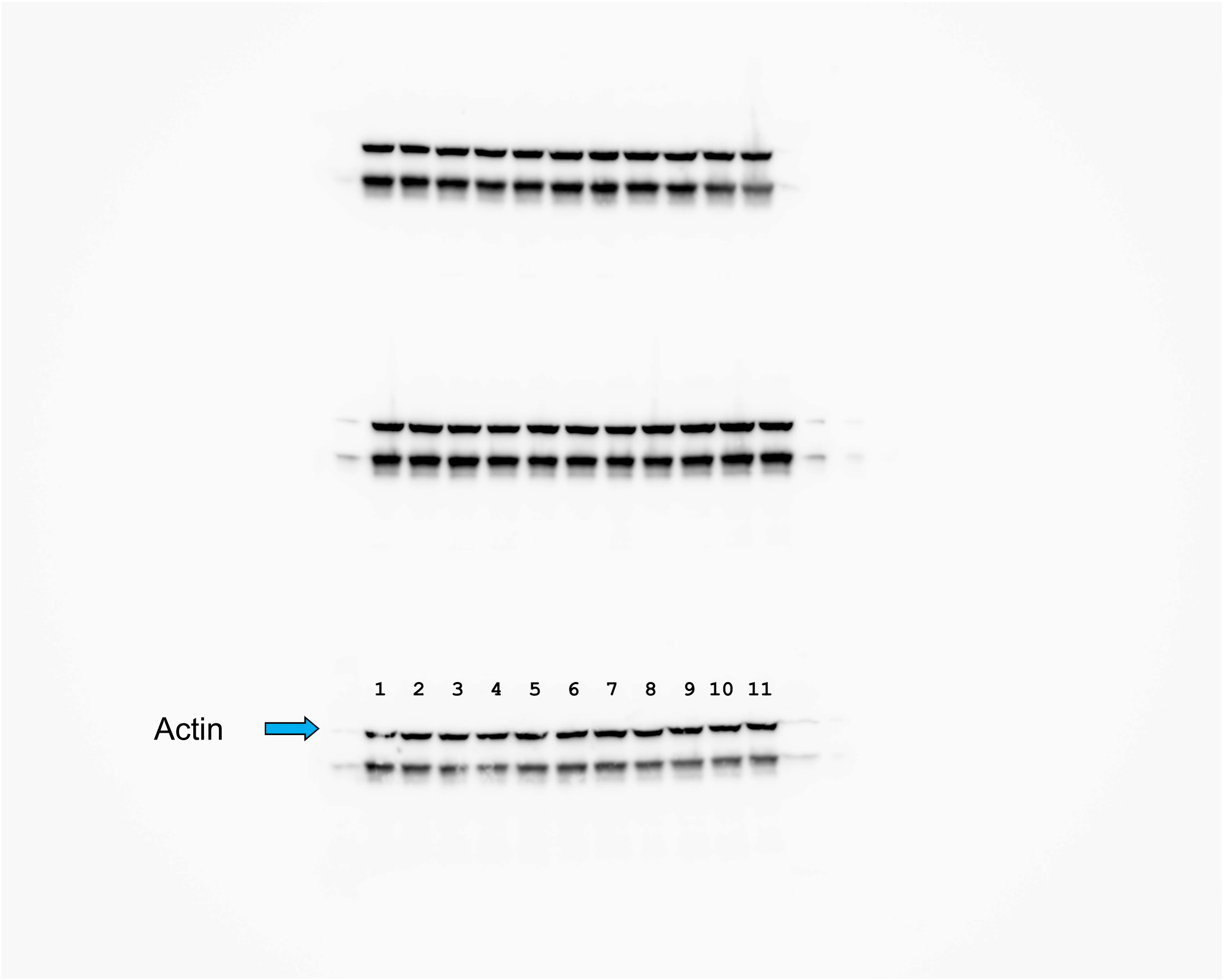

Full and uncropped Western Blot for Figure 3A (PERK)
Blots 1-4 are in the figure.

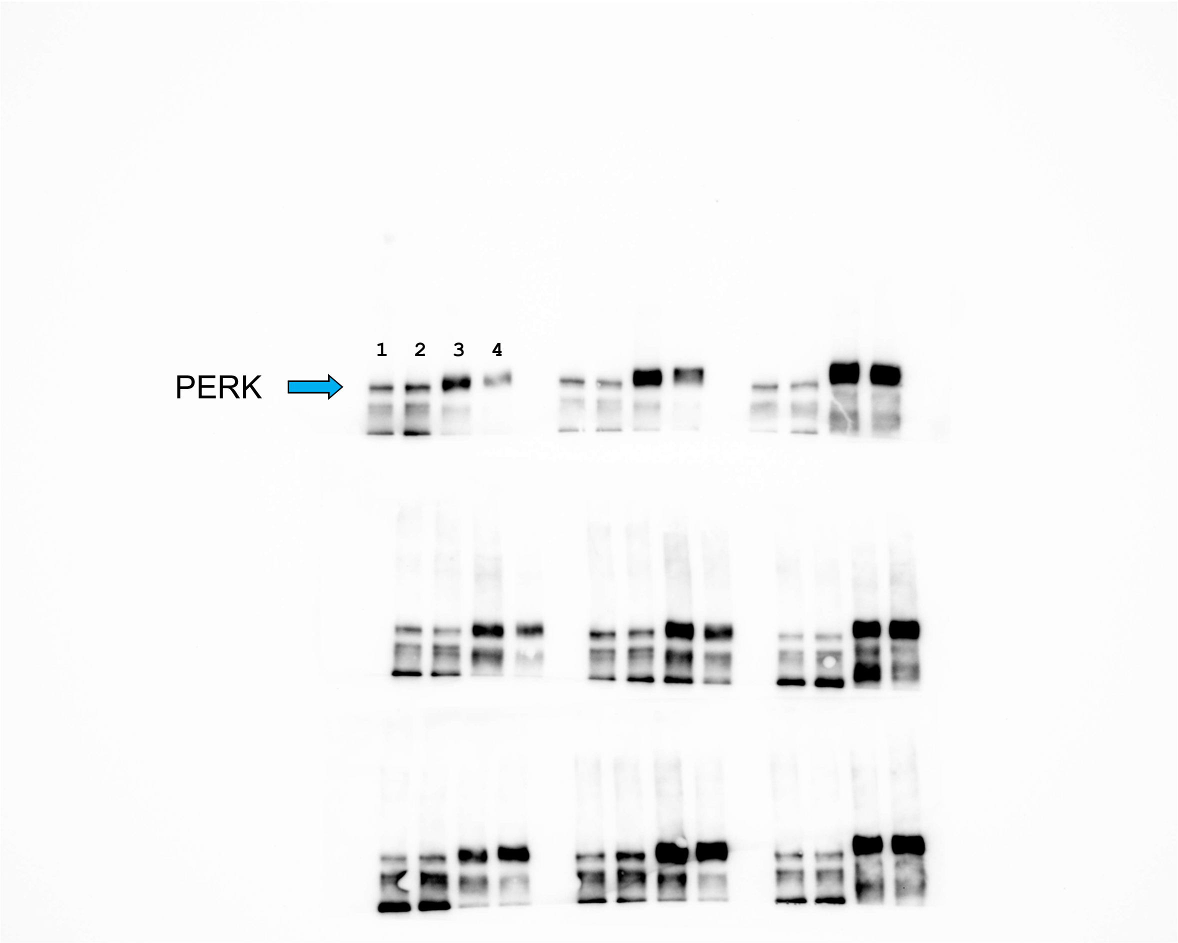

Full and uncropped Western Blot for Figure 3A (XBP1s)
Blots 1-4 are in the figure.

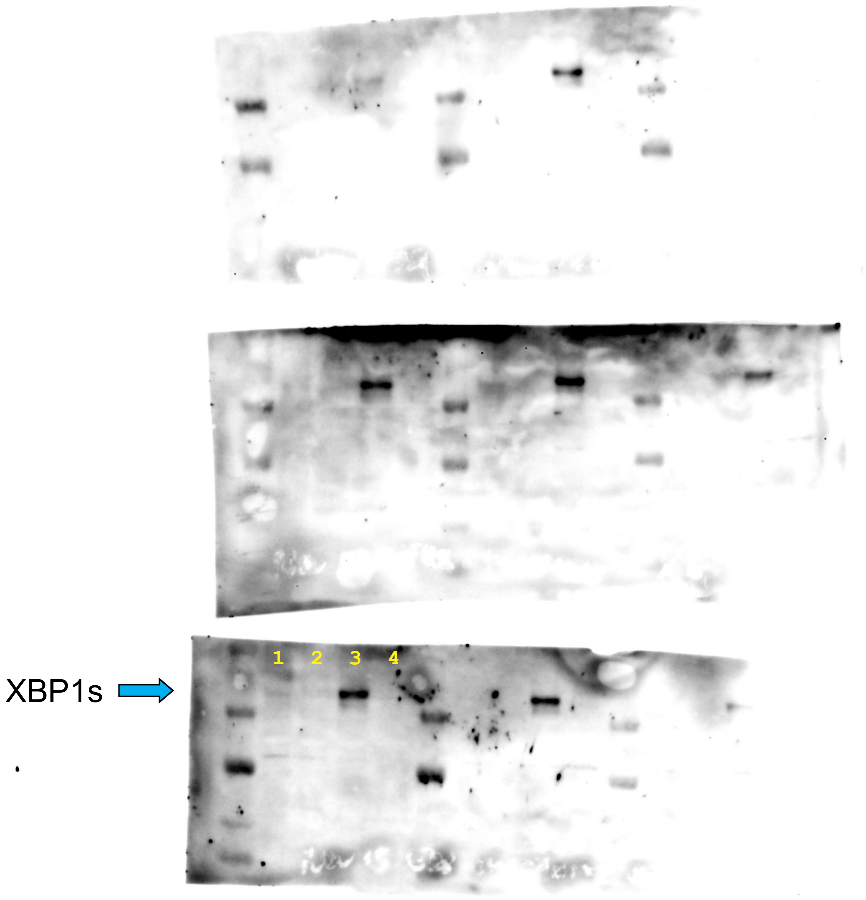

Full and uncropped Western Blot for Figure 3A (Actin)
Blots 1-4 are in the figure.

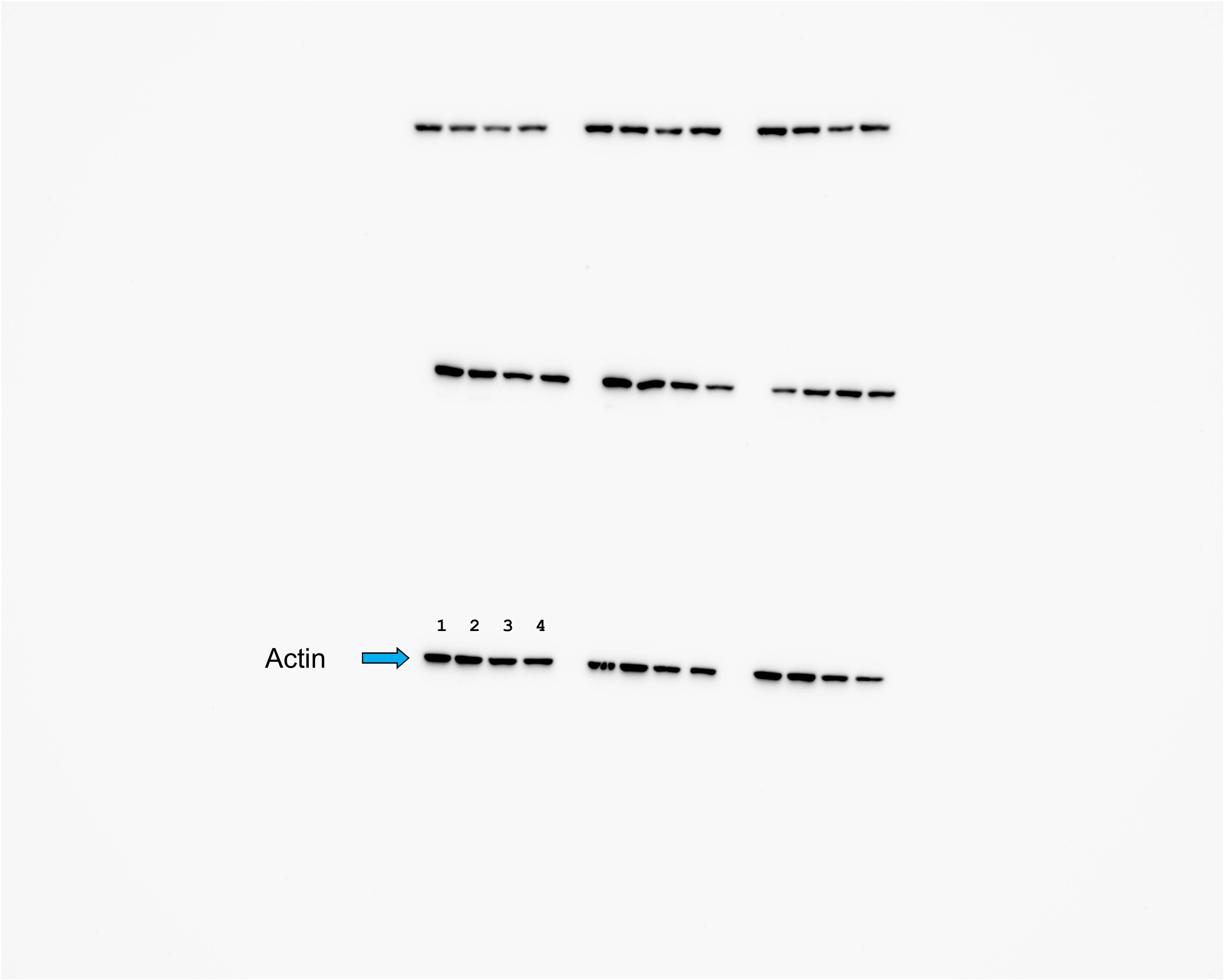

Full and uncropped Western Blot for Figure 3B (PERK)
Blots 1-4 are in the figure.

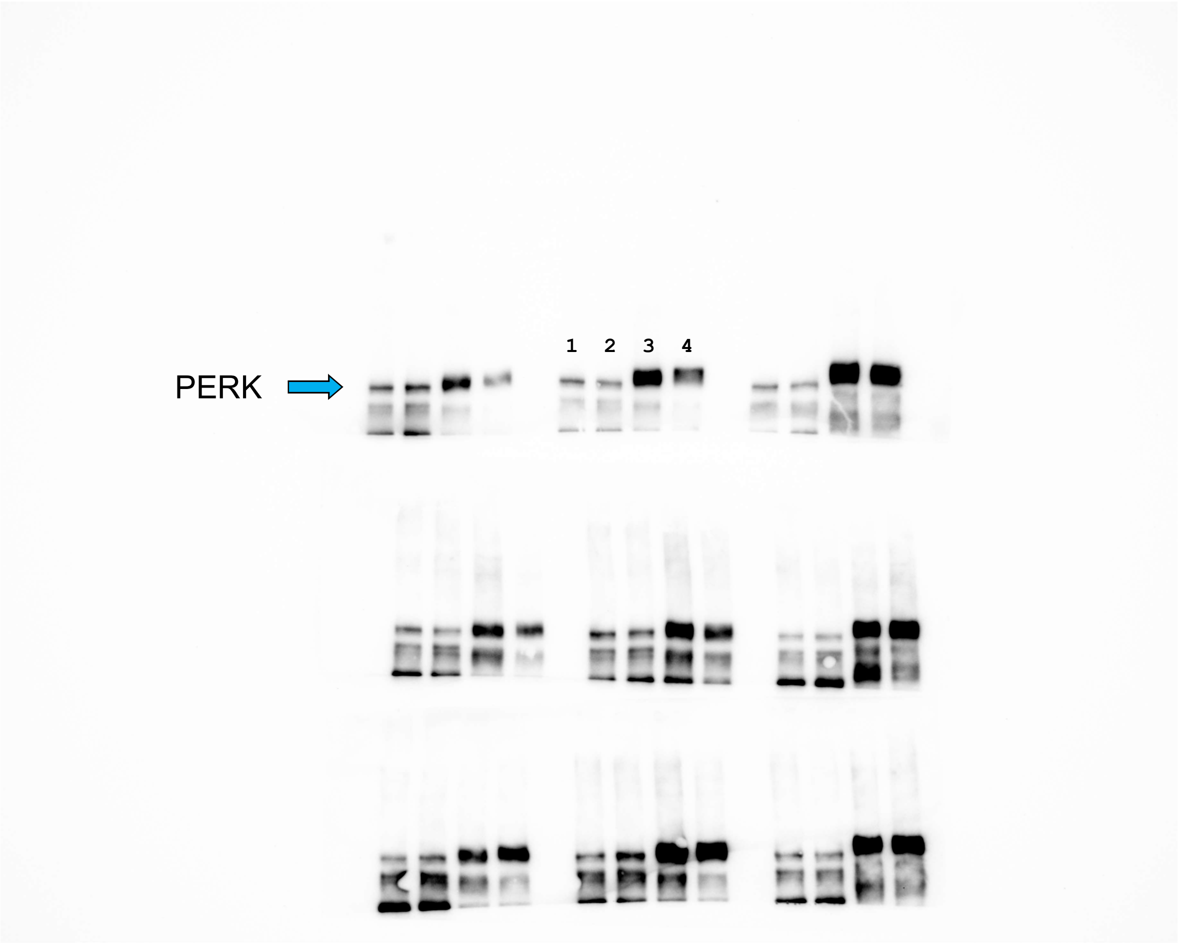

Full and uncropped Western Blot for Figure 3B (XBP1s)
Blots 1-4 are in the figure.

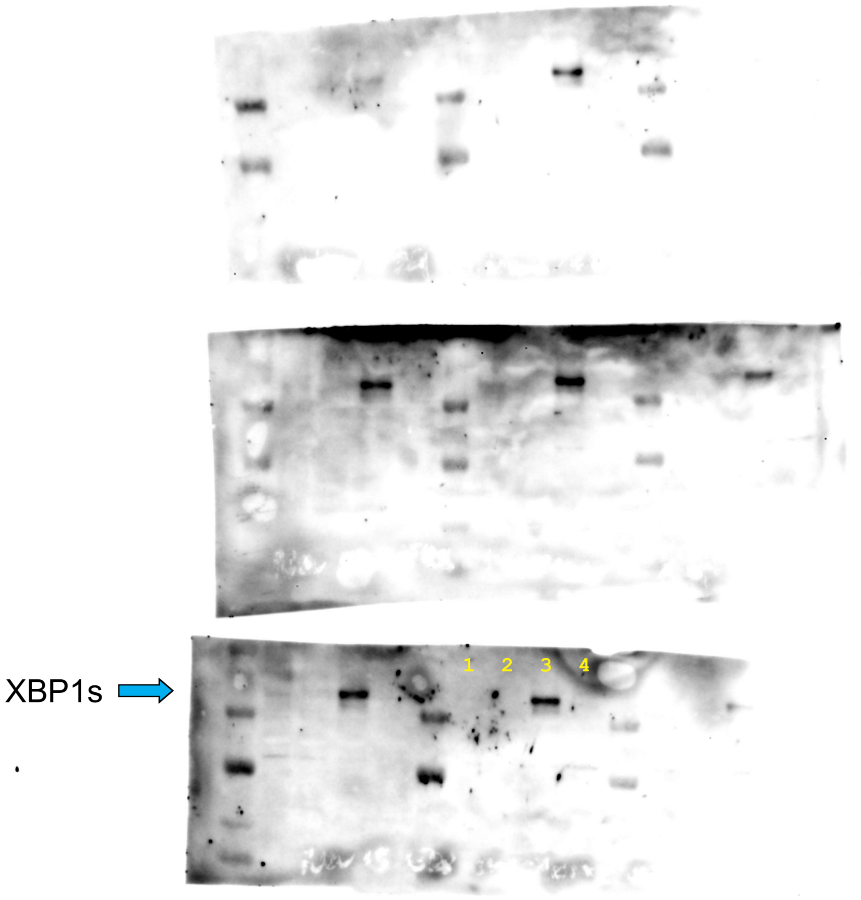

Full and uncropped Western Blot for Figure 3B (Actin)
Blots 1-4 are in the figure.

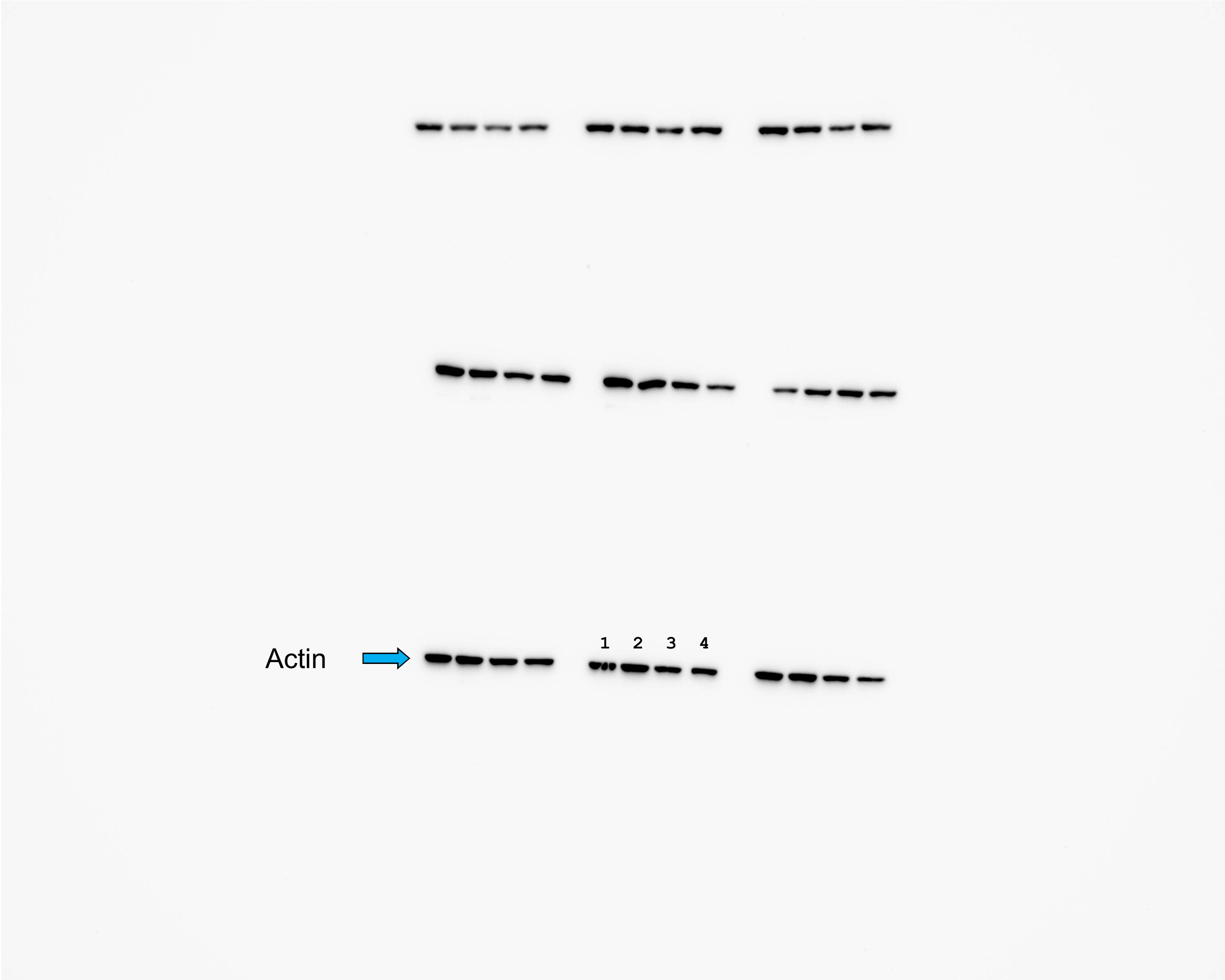

Full and uncropped Western Blot for Figure 3C (PERK)
Blots 1-4 are in the figure.

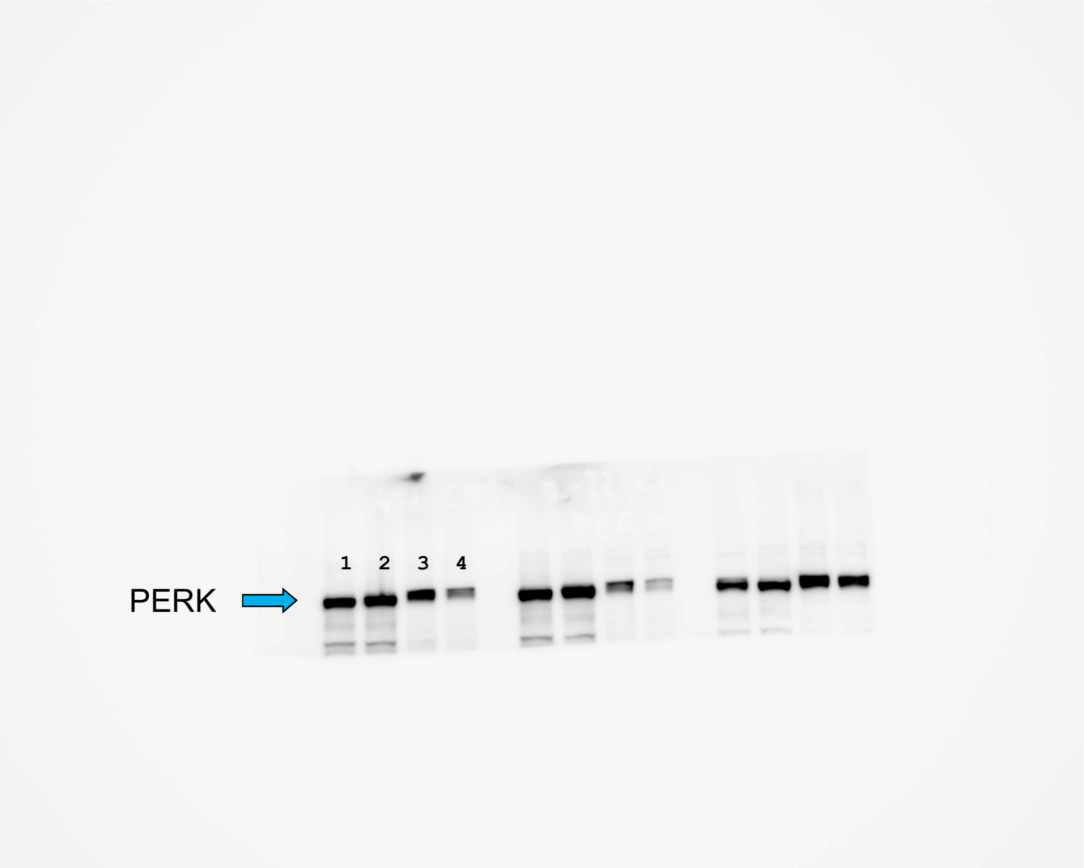

Full and uncropped Western Blot for Figure 3C (XBP1s)

Blots 1-4 are in the figure.

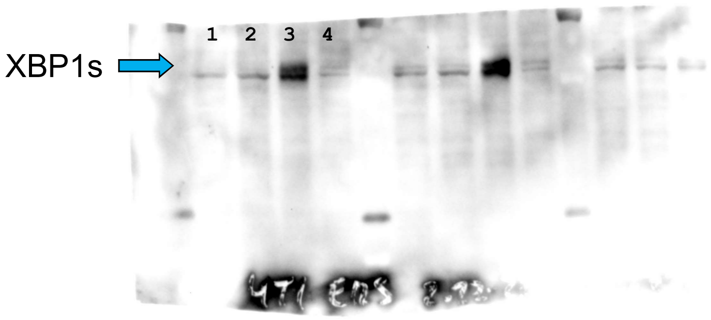

Full and uncropped Western Blot for Figure 3C (Actin)
Blots 1-4 are in the figure.

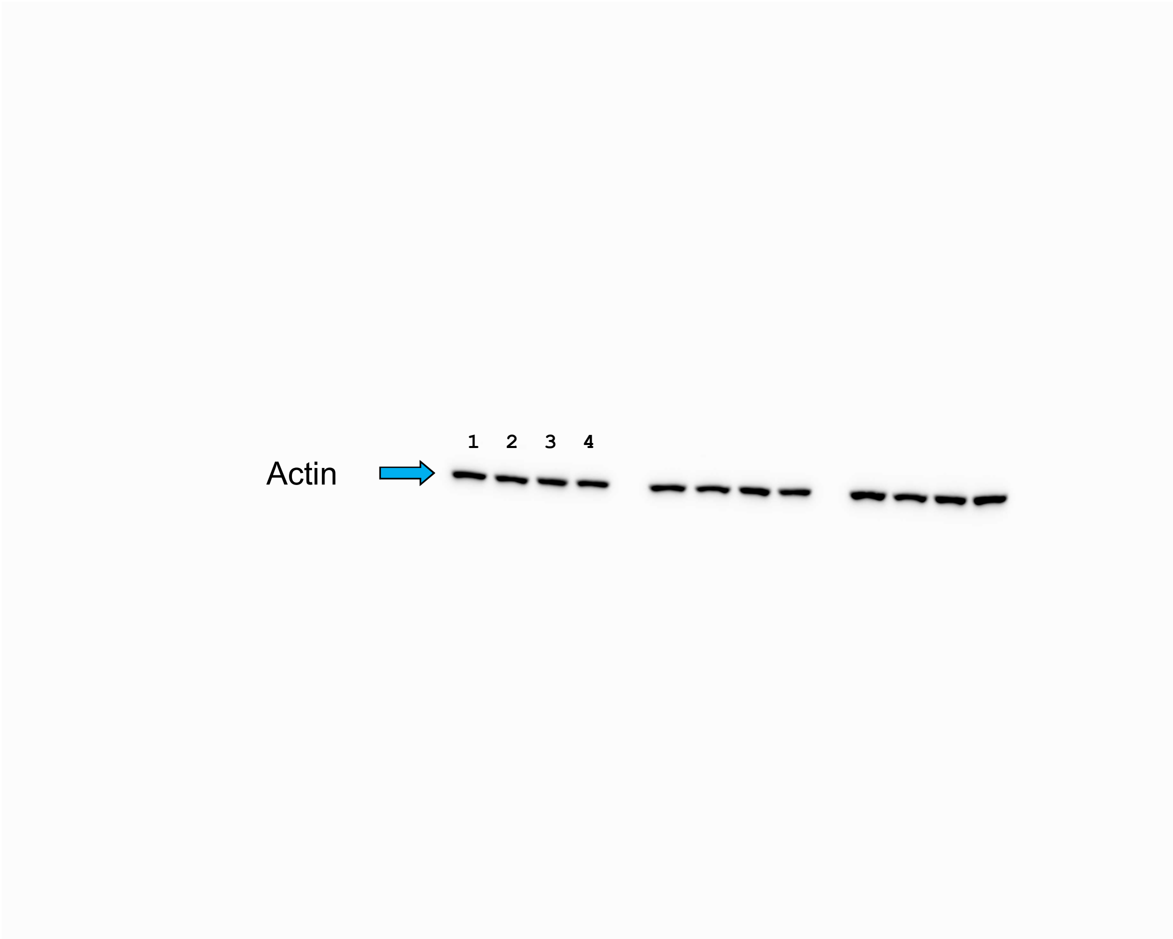

Full and uncropped Western Blot for Figure 3D (PERK)

Blots 1-4 are in the figure.

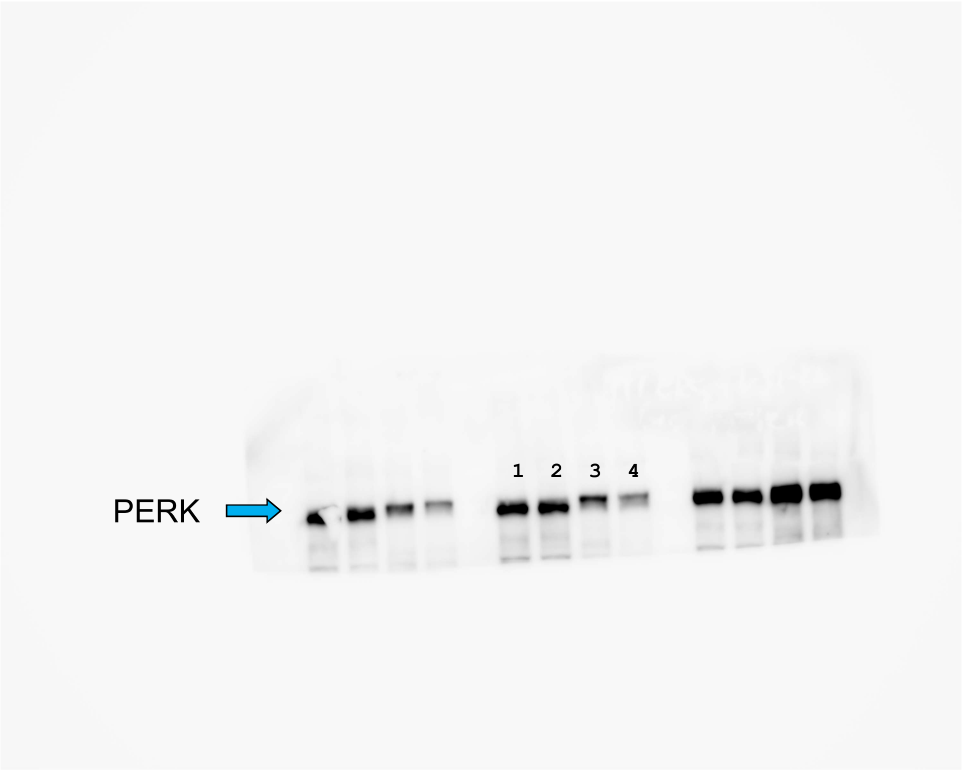

Full and uncropped Western Blot for Figure 3D (XBP1s)
Blots 1-4 are in the figure.

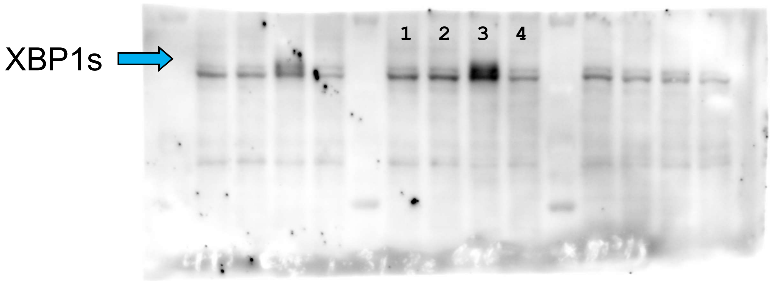

Full and uncropped Western Blot for Figure 3D (Actin)
Blots 1-4 are in the figure.

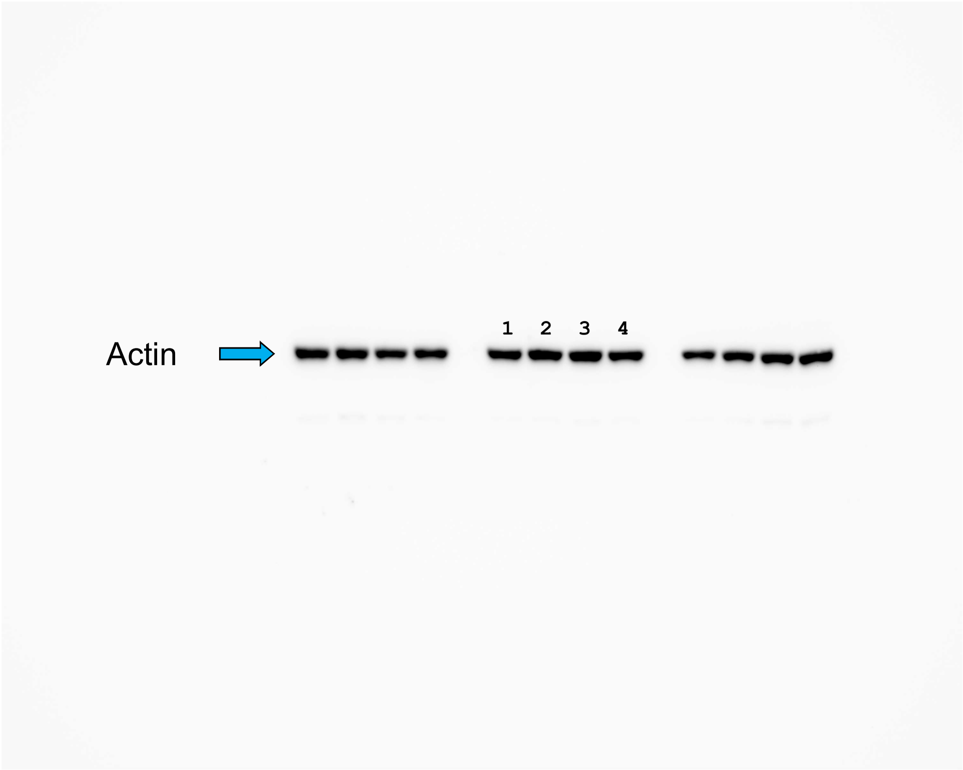

Full and uncropped Western Blot for Figure 3E (PERK)
Blots 1-12 are in the figure.

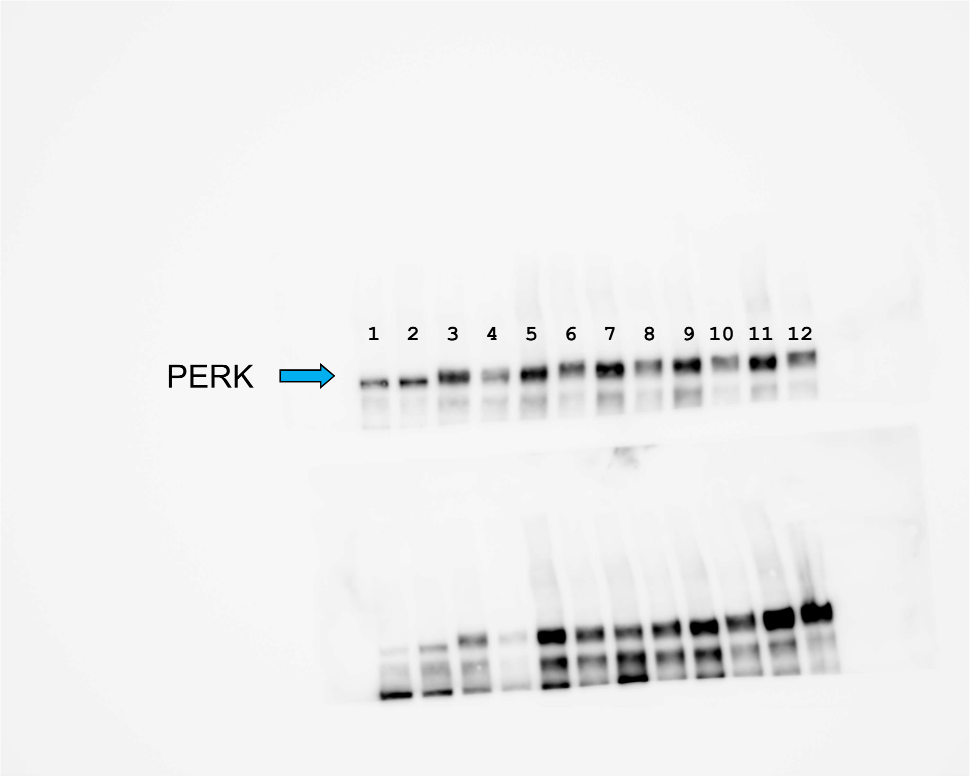

Full and uncropped Western Blot for Figure 3E (XBP1s)

Blots 1-12 are in the figure.

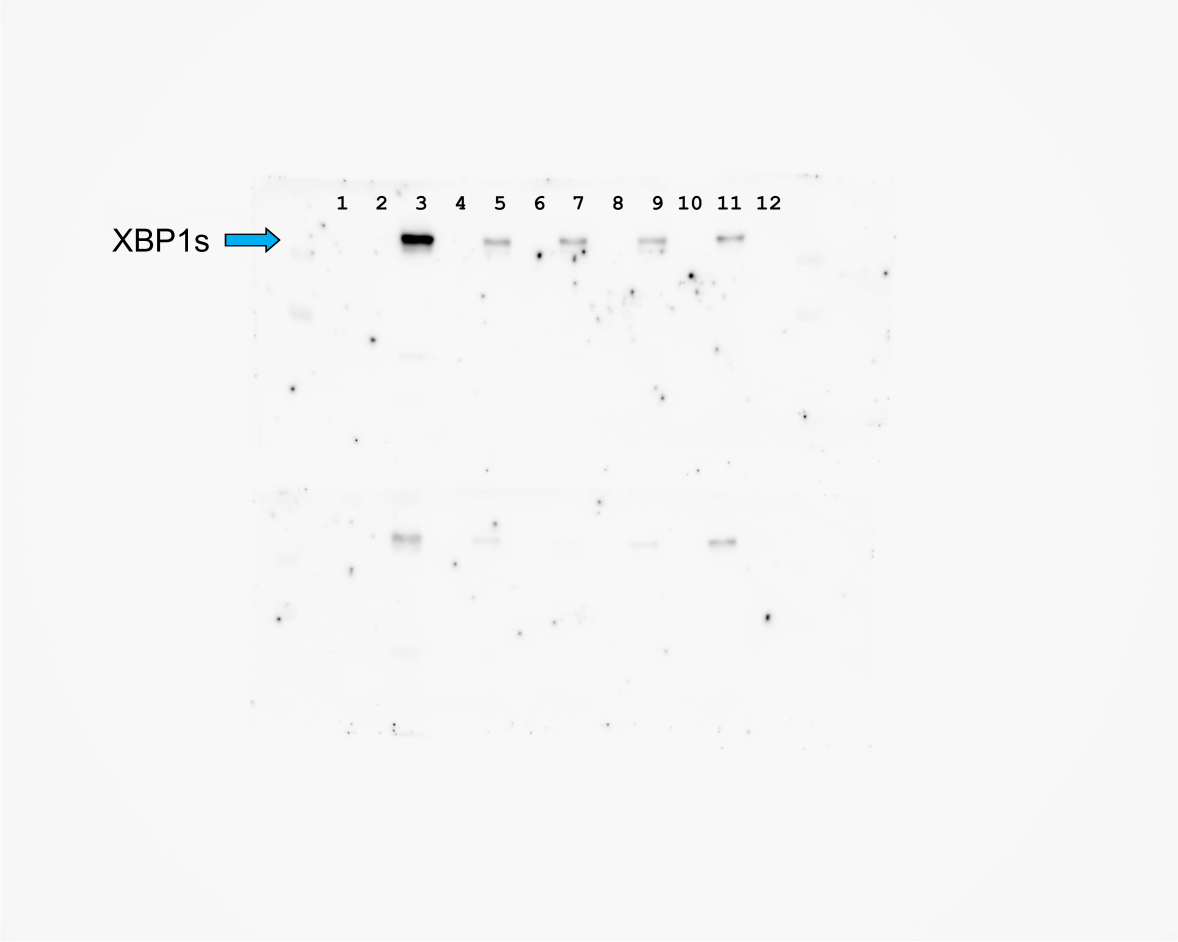

Full and uncropped Western Blot for Figure 3E (Actin)

Blots 1-12 are in the figure.

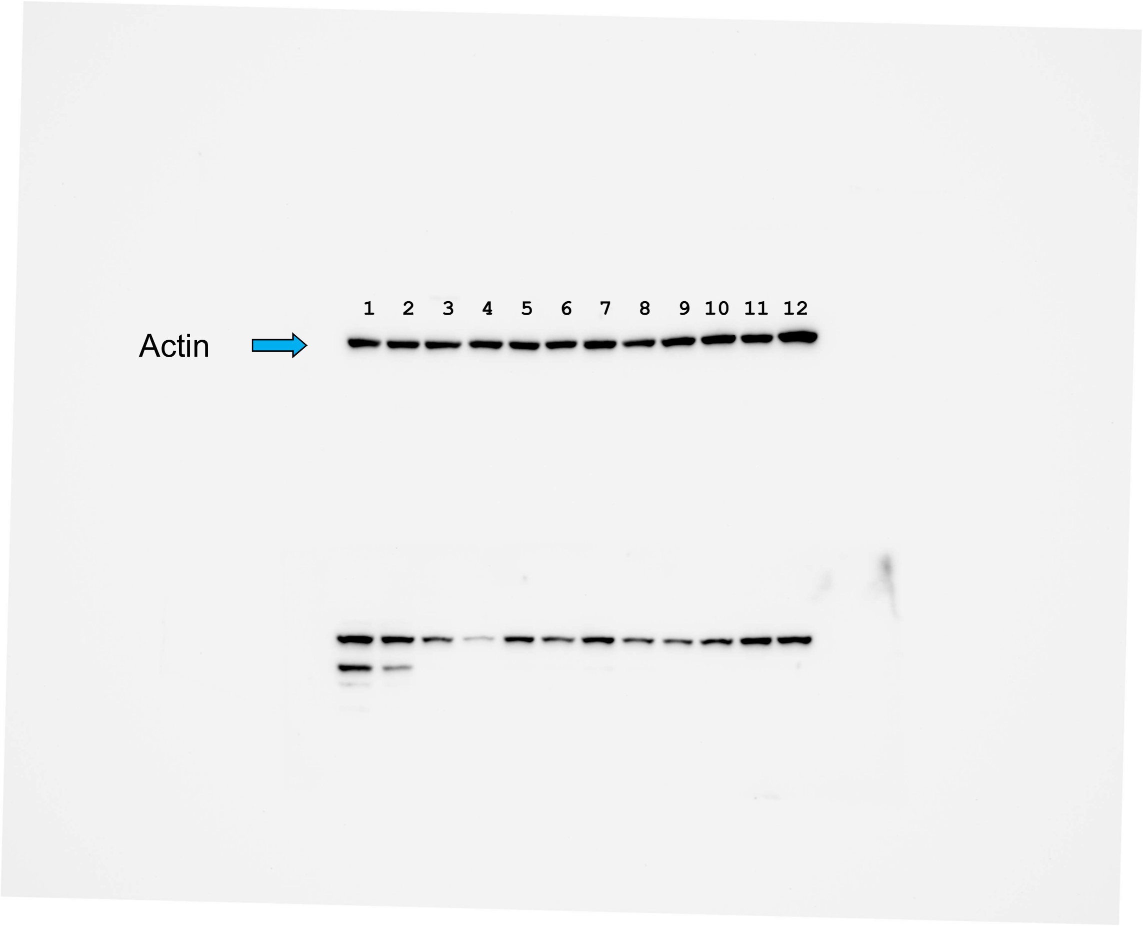

Full and uncropped Western Blot for Figure 3F (PERK)
Blots 1-12 are in the figure.

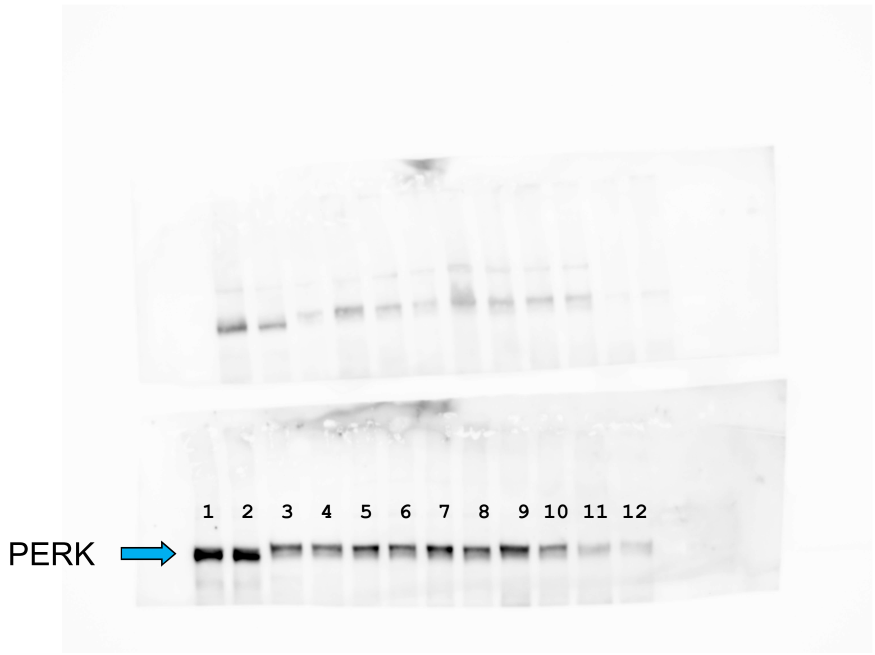

Full and uncropped Western Blot for Figure 3F (XBP1s)
Blots 1-12 are in the figure.

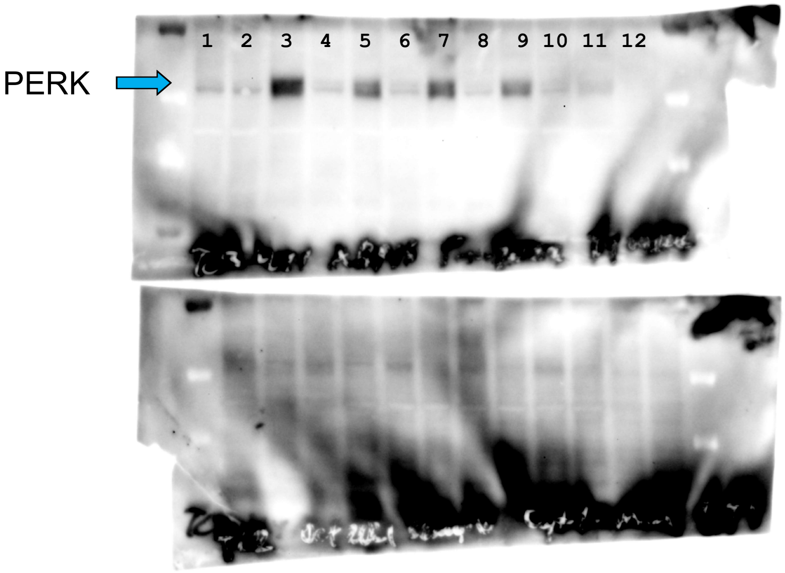

Full and uncropped Western Blot for Figure 3F (Actin)

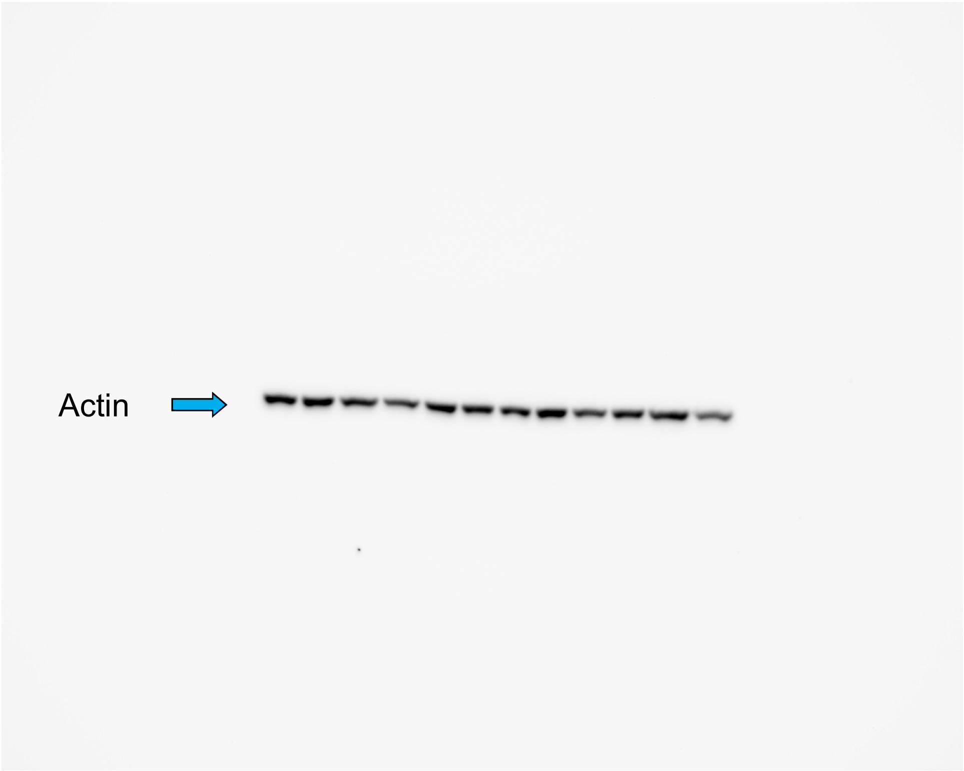

Full and uncropped Western Blot for Figure 4A (PERK)

PERK

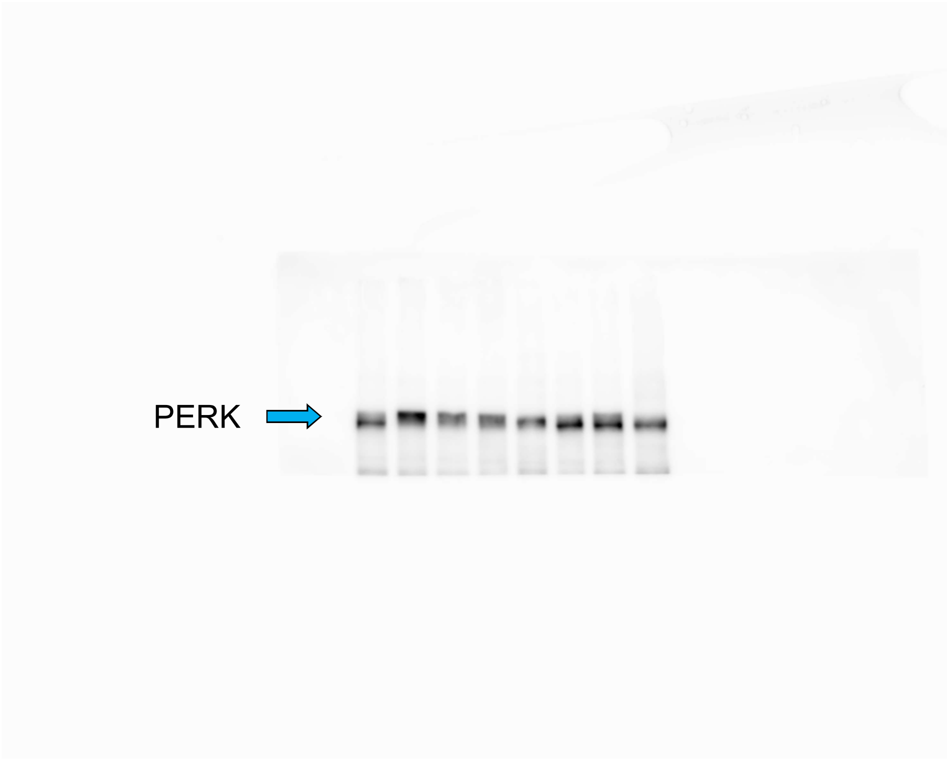

Full and uncropped Western Blot for Figure 4A (IRE1)
Blots 1-8 are in the figure.

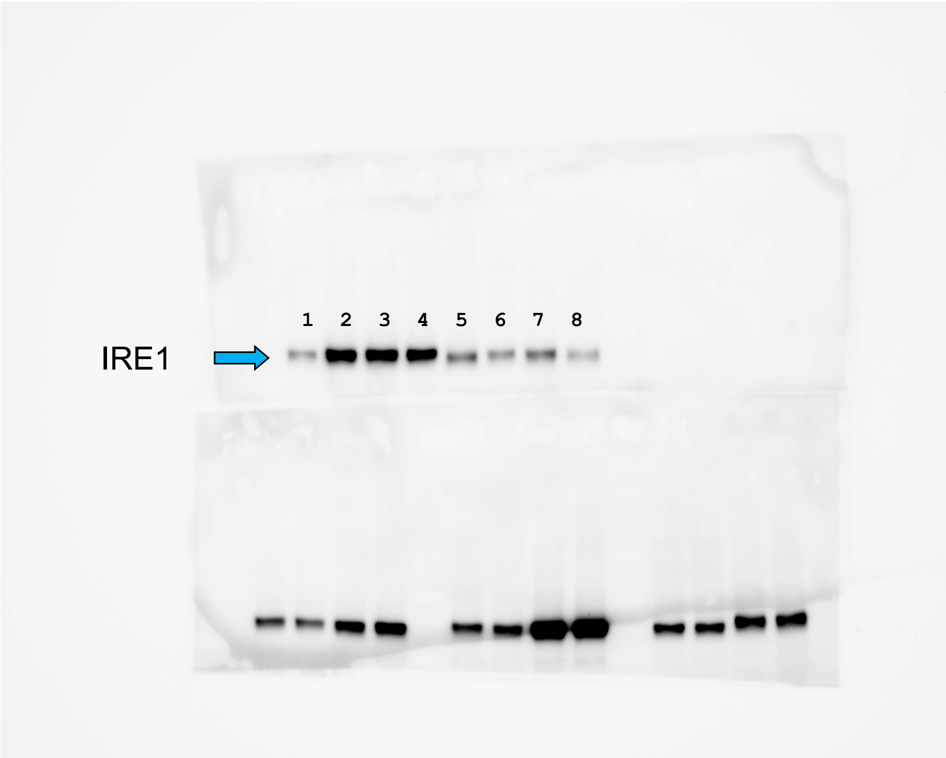

Full and uncropped Western Blot for Figure 4A (XBP1s)

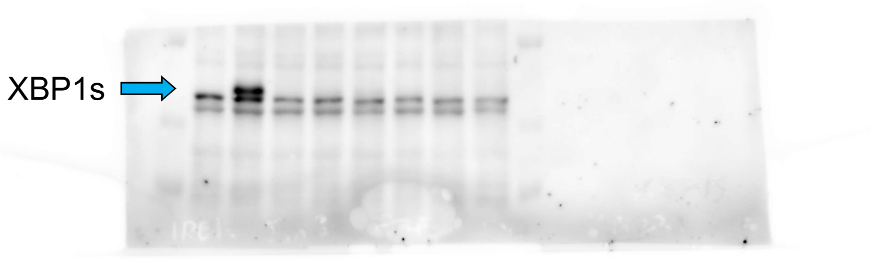

Full and uncropped Western Blot for Figure 4A (Actin)
Blots 1-8 are in the figure.

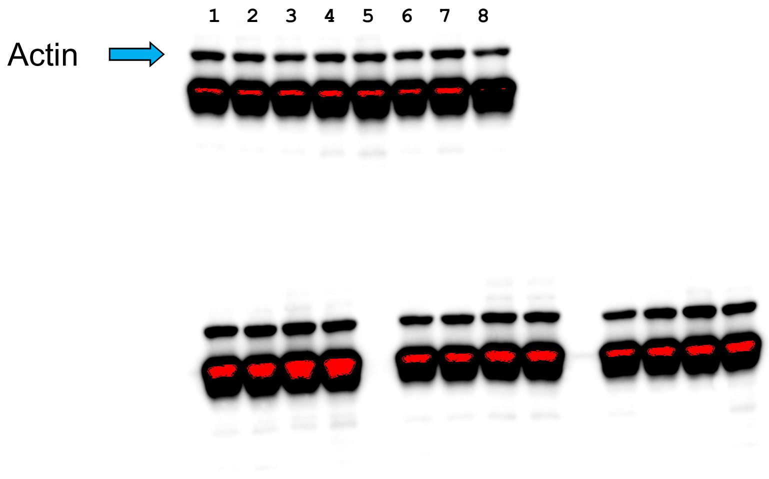

Full and uncropped Western Blot for Figure 4B (PERK)

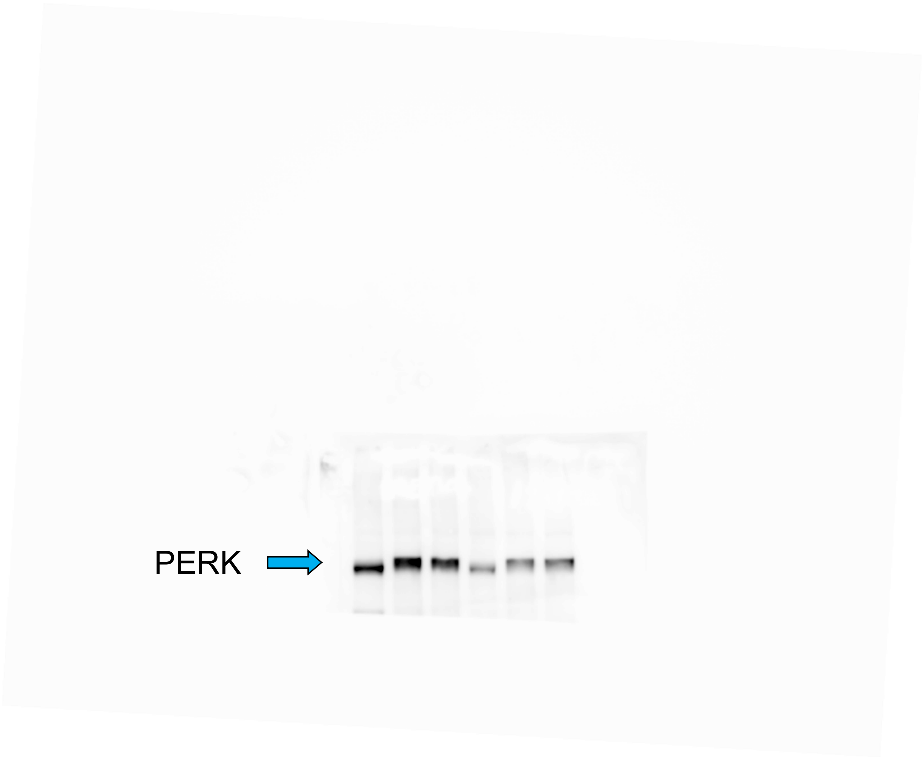

Full and uncropped Western Blot for Figure 4B (IRE1)

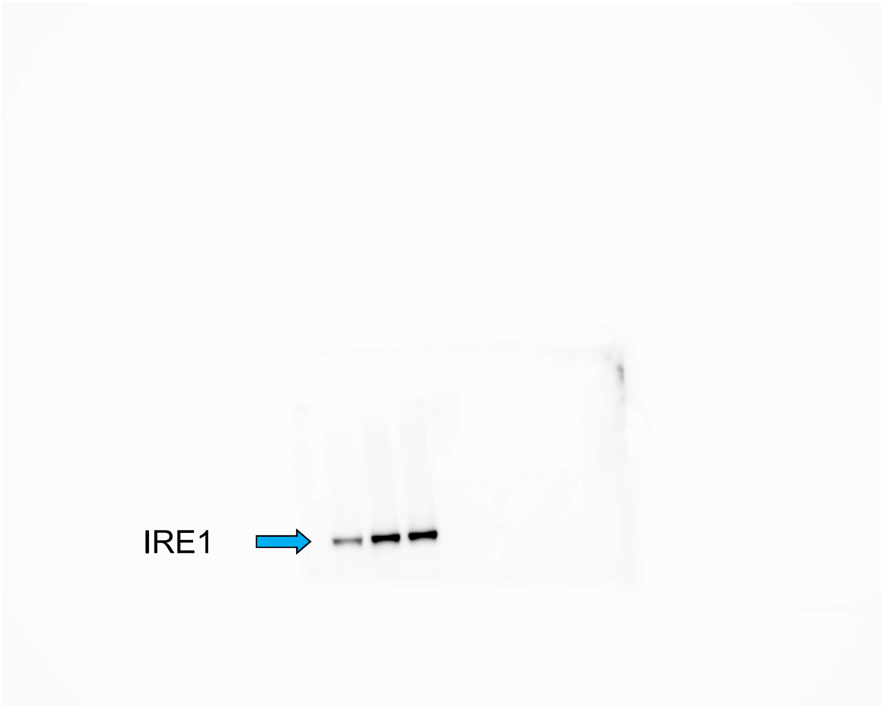

Full and uncropped Western Blot for Figure 4B (XBP1s)

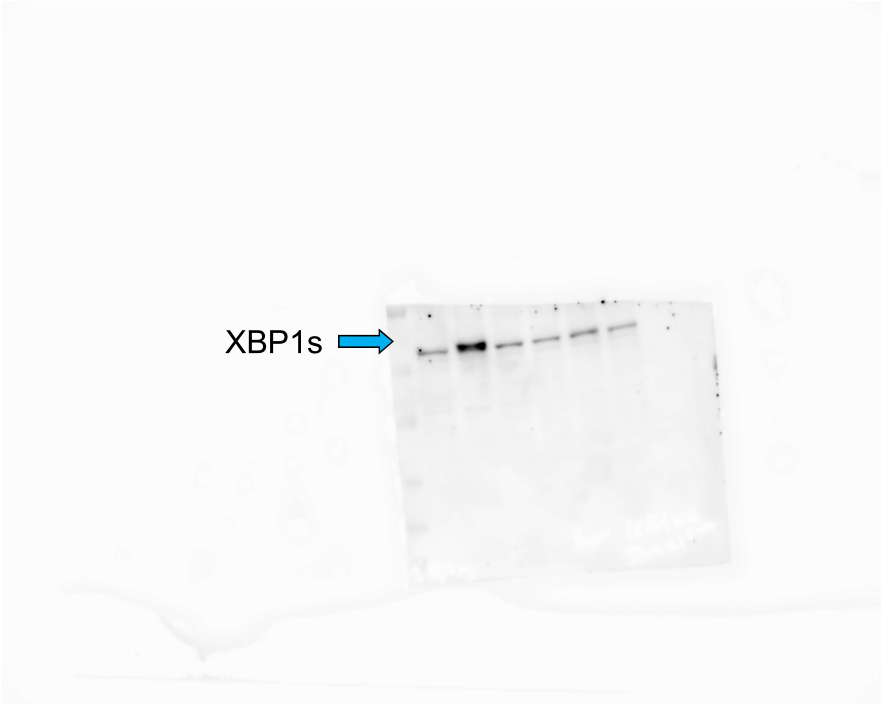

Full and uncropped Western Blot for Figure 4B (Actin)
Blots 1-6 are in the figure.

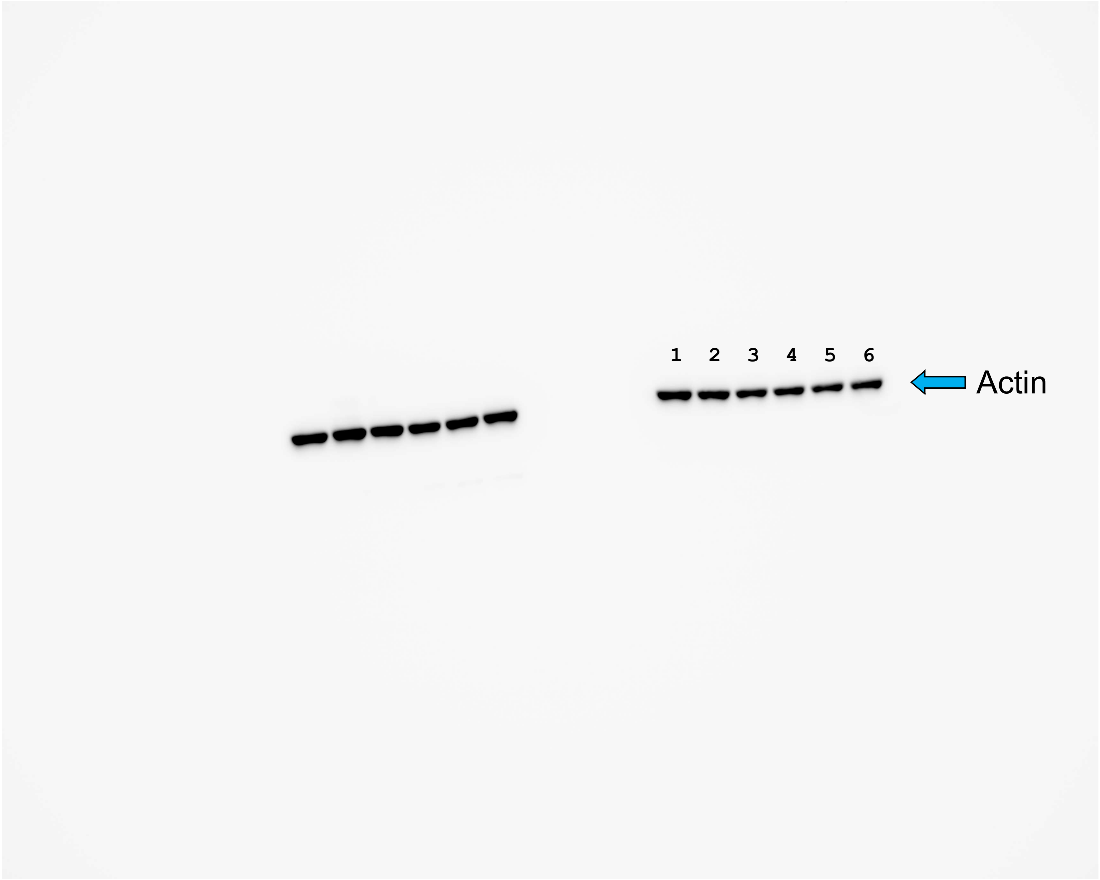

Full and uncropped Western Blot for Figure 6A (PERK)

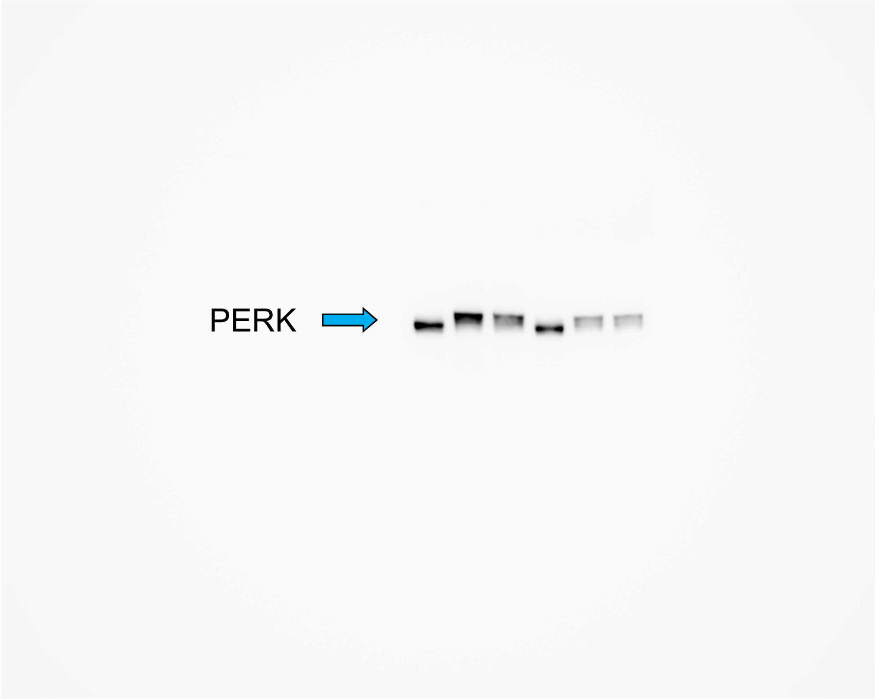

Full and uncropped Western Blot for Figure 6A (IRE1)

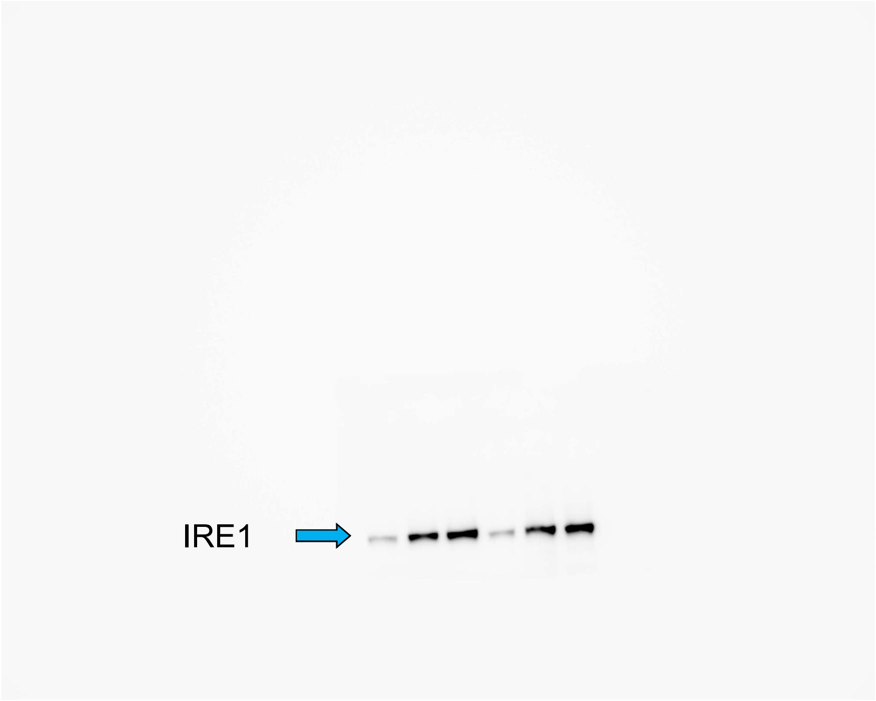

Full and uncropped Western Blot for Figure 6A (XBP1s)

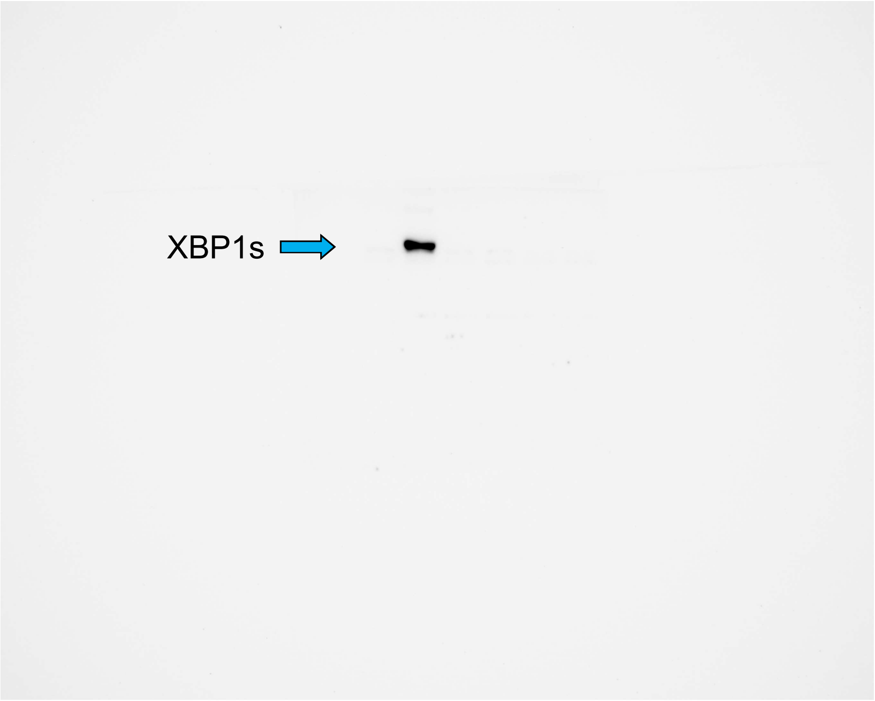

Full and uncropped Western Blot for Figure 6A (Actin)

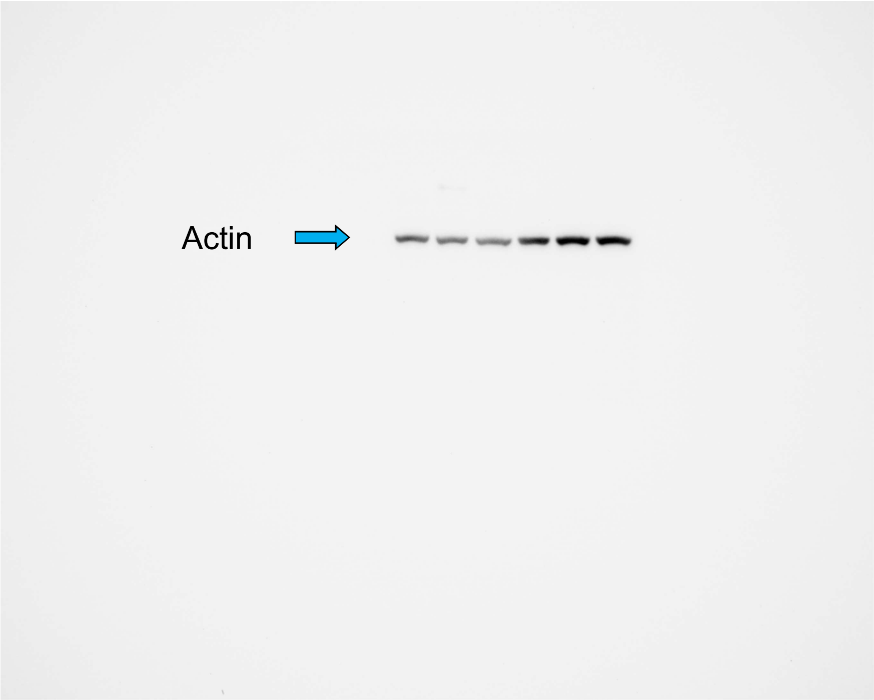

Full and uncropped Western Blot for Figure 6E (PERK)
Blots 1-5 are in the figure.

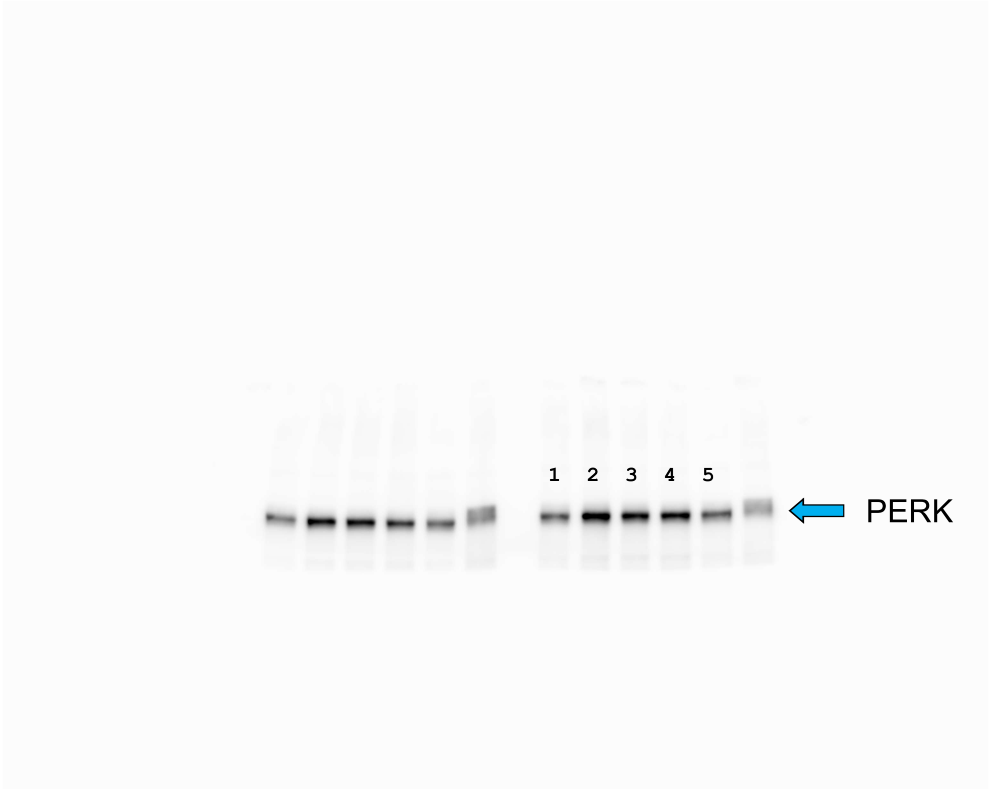

Full and uncropped Western Blot for Figure 6E (XBP1s)
Blots 1-5 are in the figure.

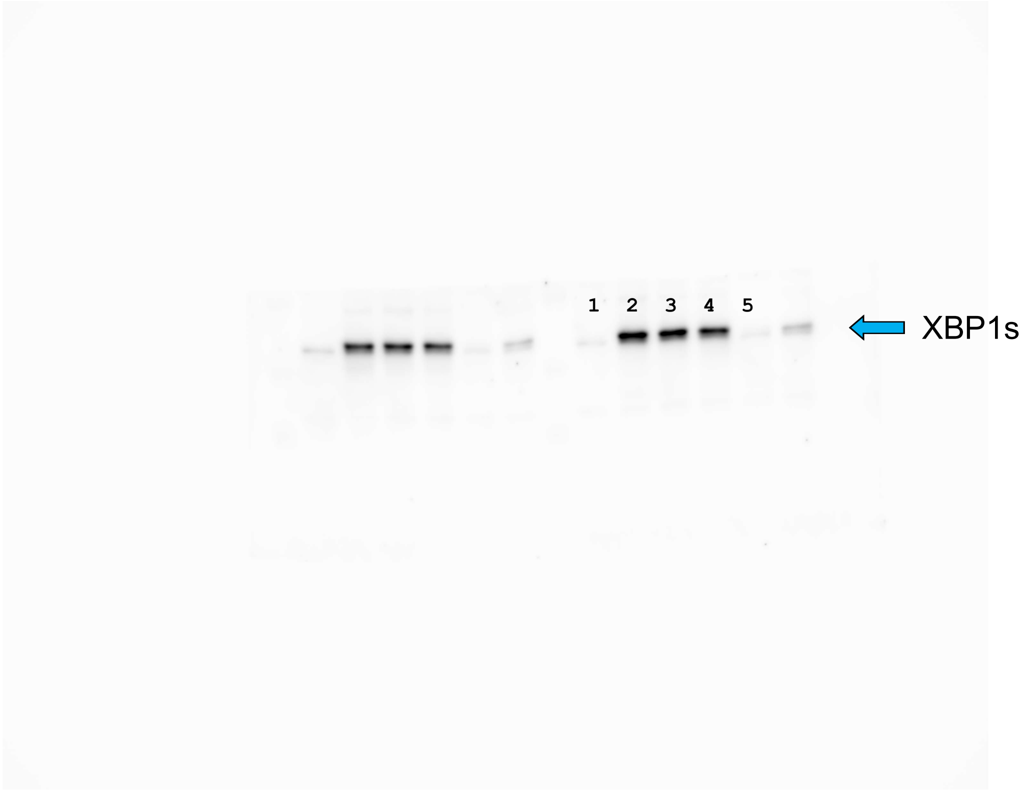

Full and uncropped Western Blot for Figure 6E (Actin)
Blots 1-5 are in the figure.

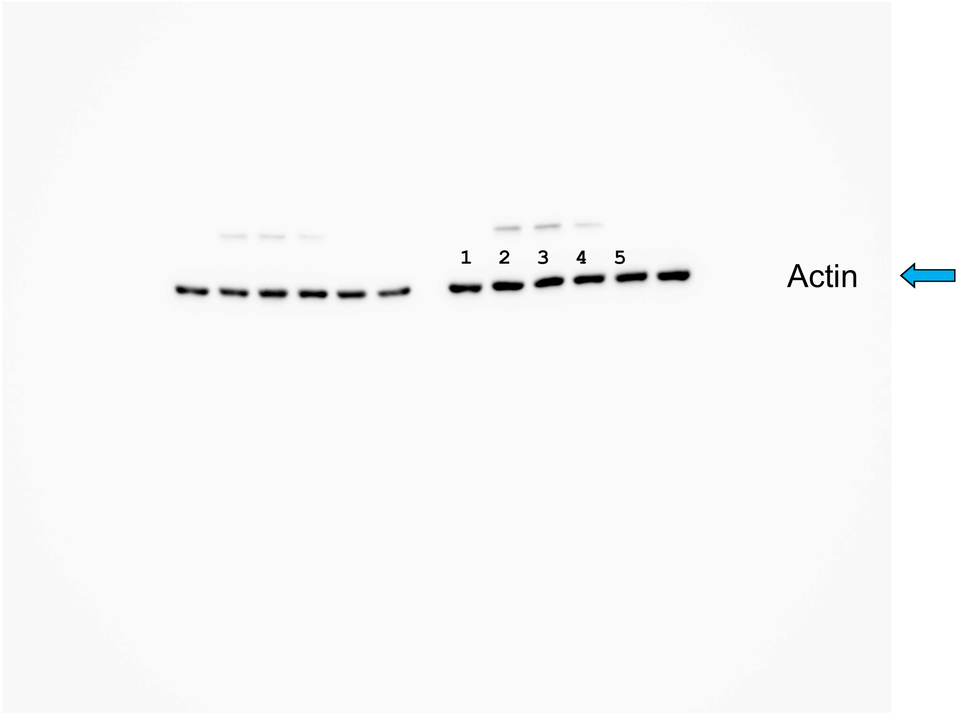

Full and uncropped Western Blot for Figure 8A (PERK)

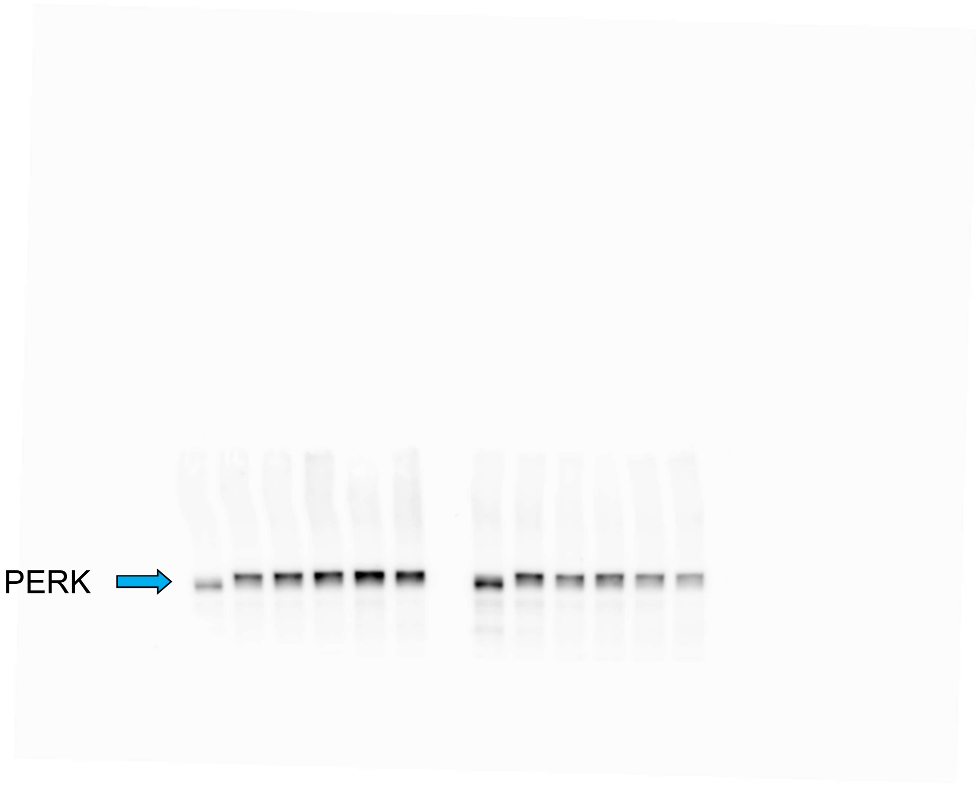

Full and uncropped Western Blot for Figure 8A (P-eif2a)

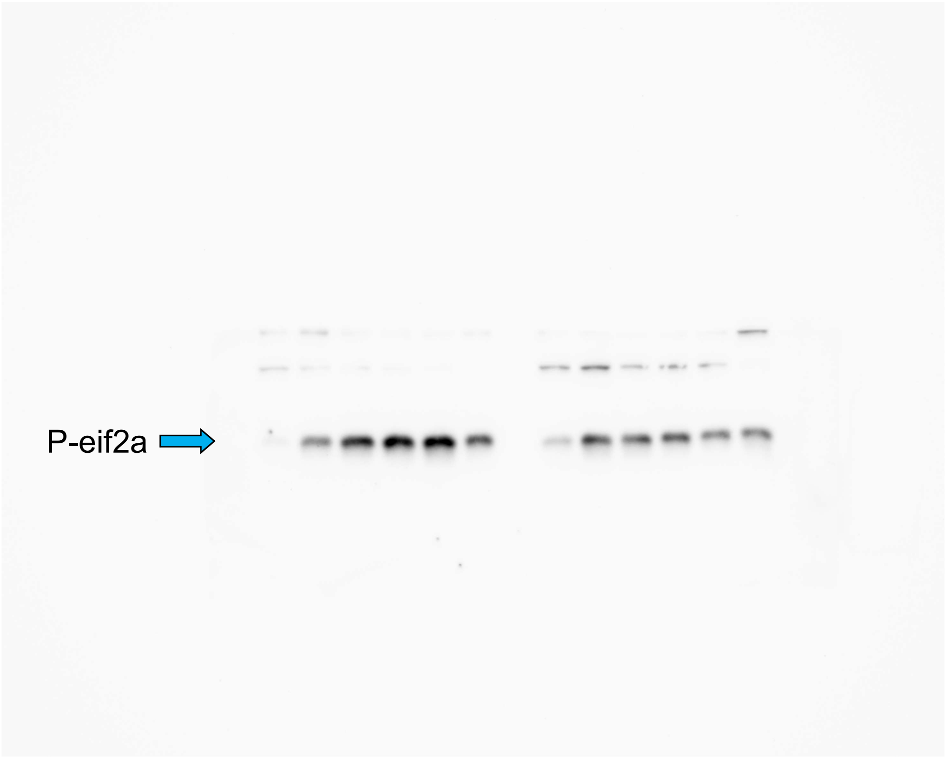

Full and uncropped Western Blot for Figure 8A (eif2a)
Blots 1-12 are in the figure.

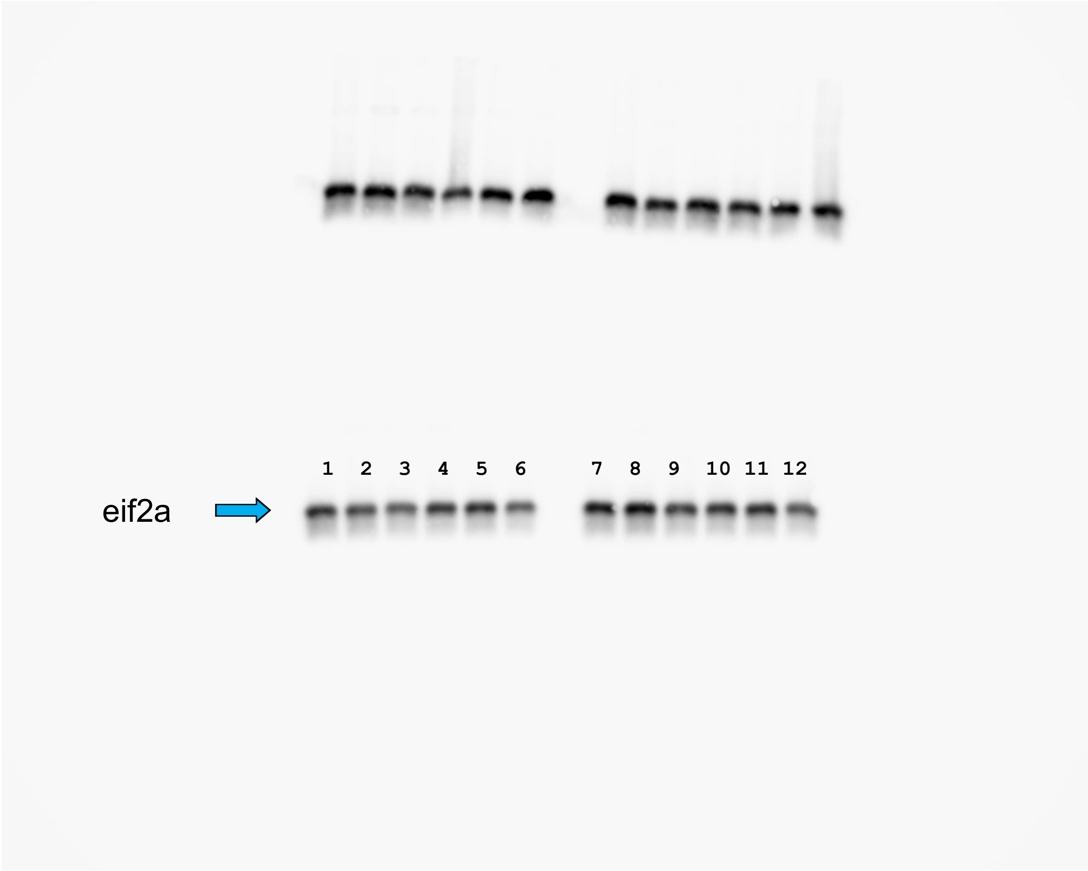

Full and uncropped Western Blot for Figure 8A (XBP1s)

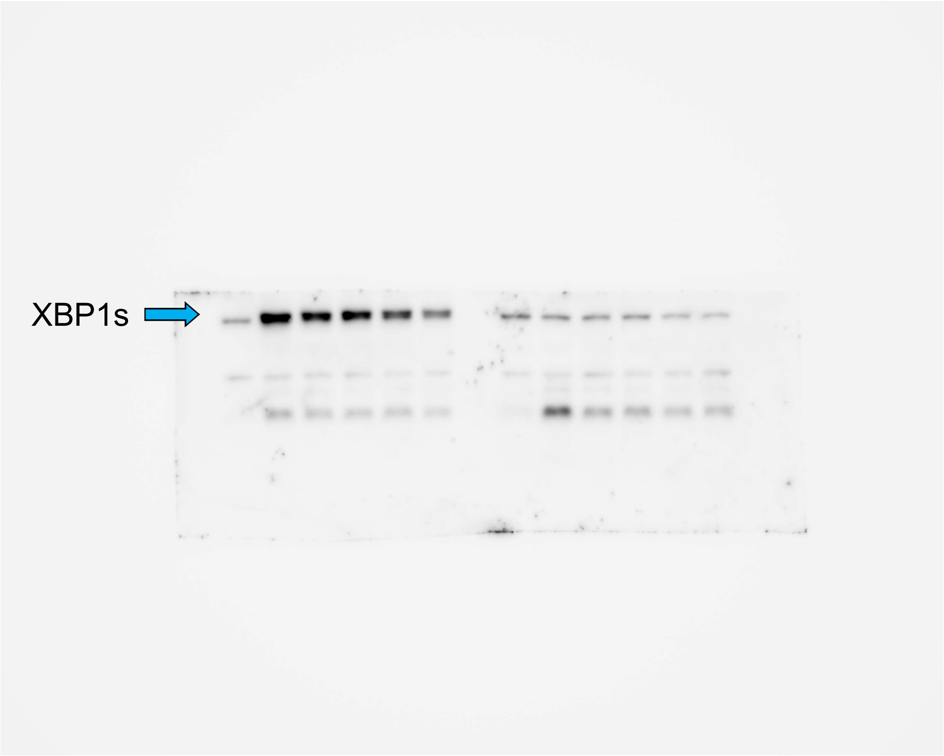

Full and uncropped Western Blot for Figure 8A (Actin)
Blots 1-12 are in the figure.

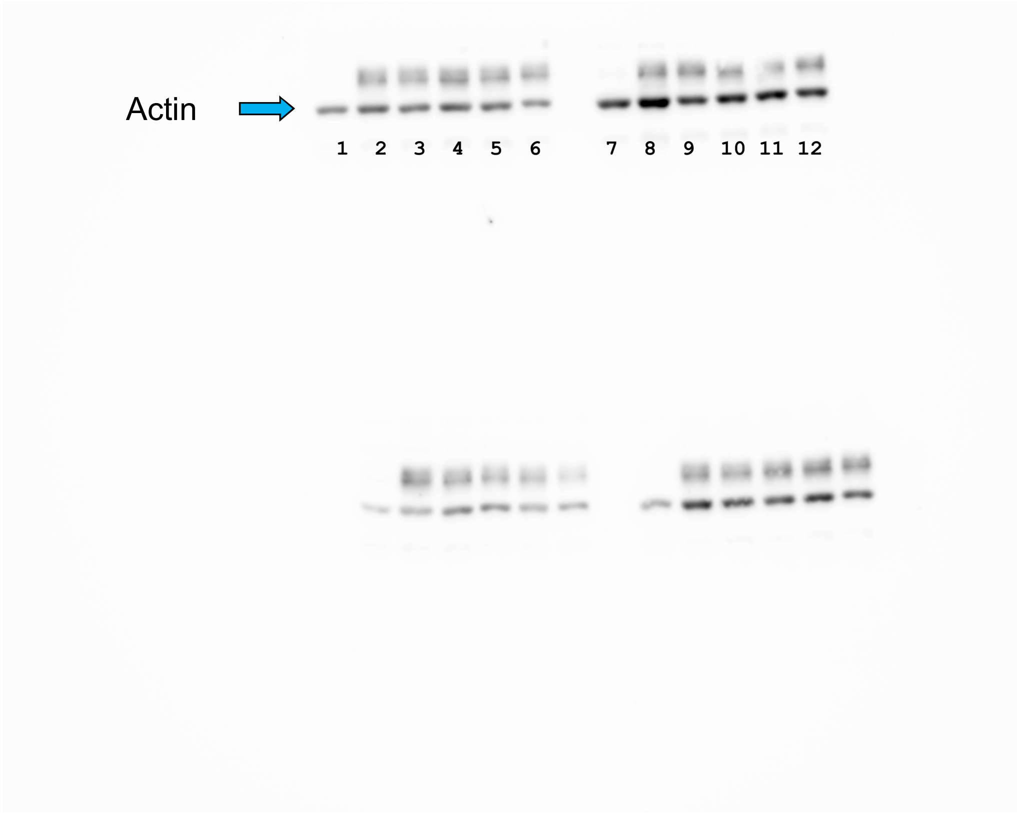

Full and uncropped Western Blot for Figure 8B (PERK)

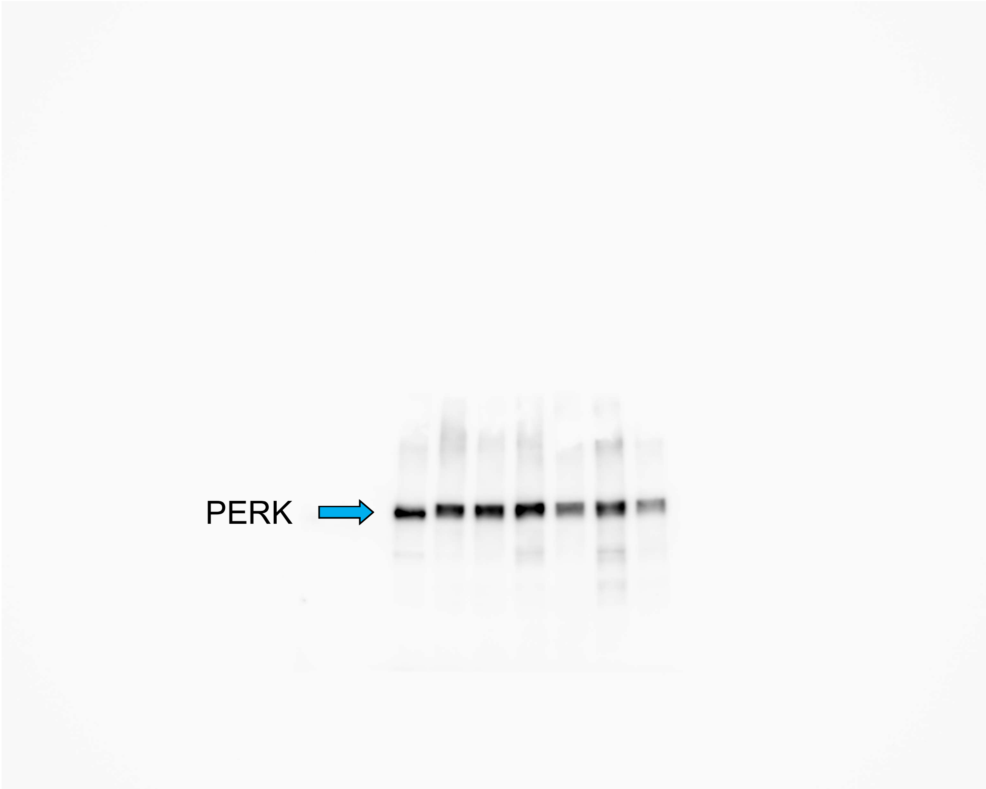

Full and uncropped Western Blot for Figure 8B (P-eif2a)

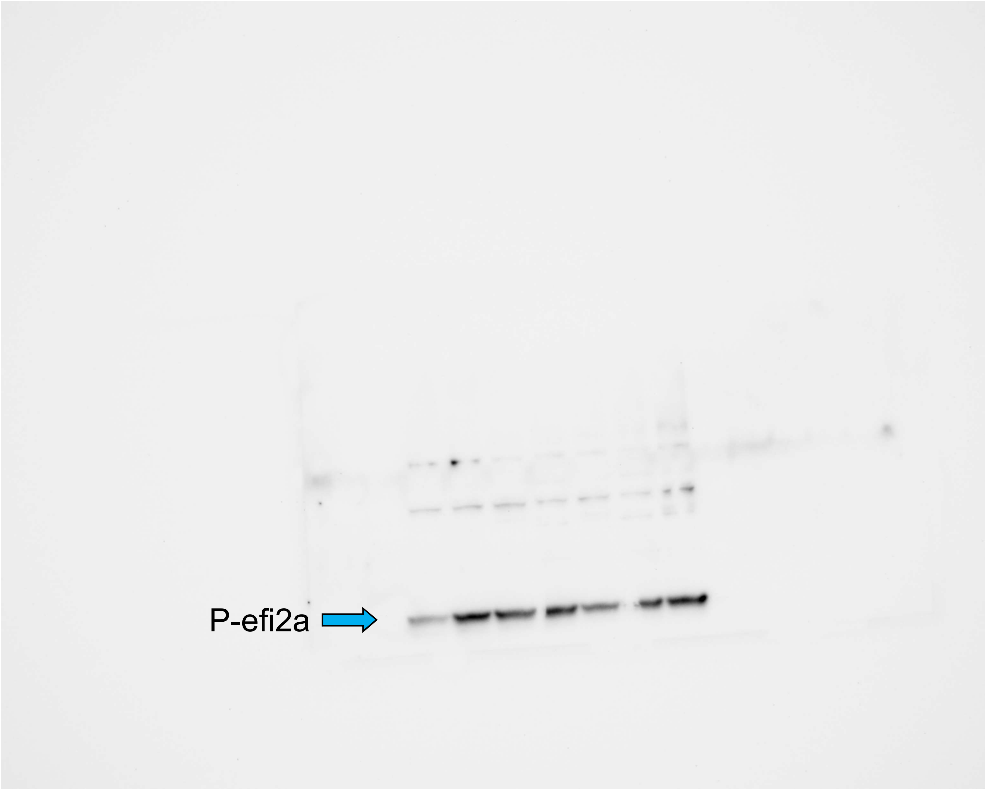

Full and uncropped Western Blot for Figure 8B (eif2a)

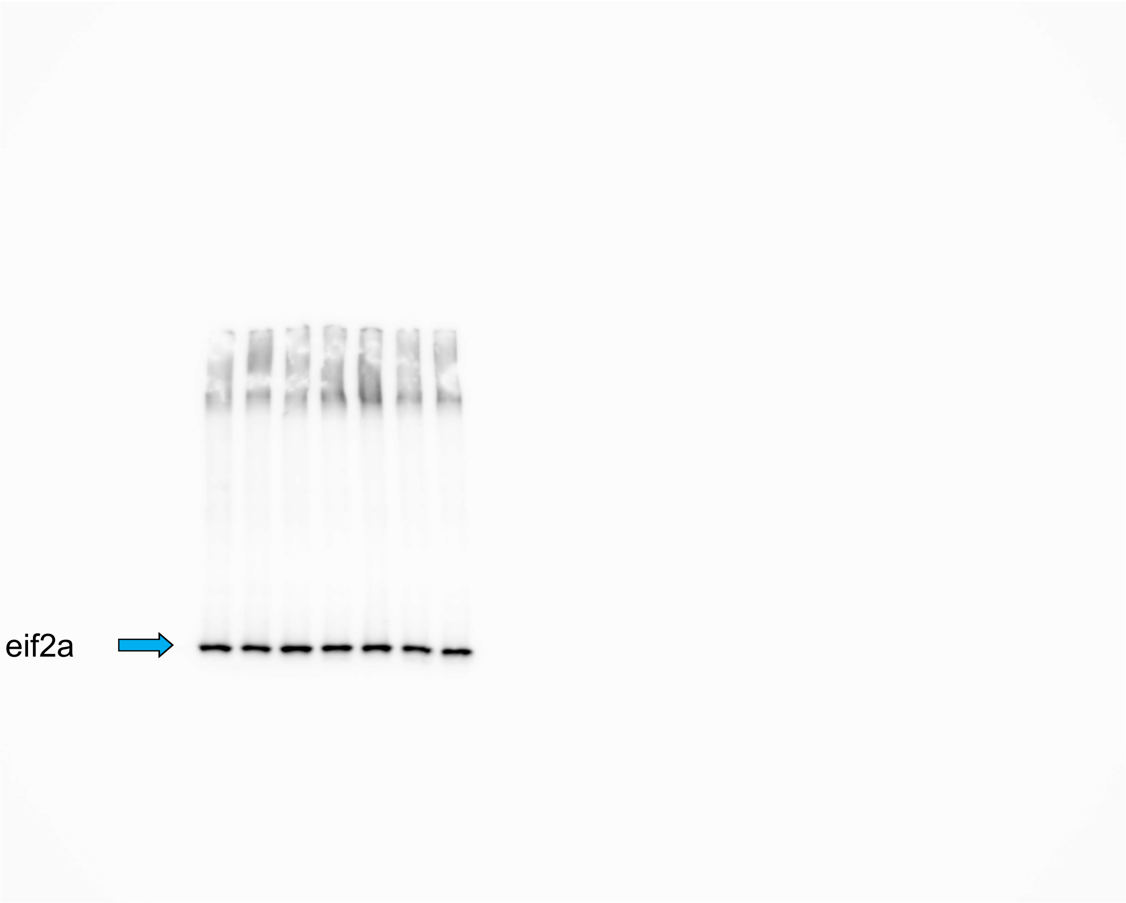

Full and uncropped Western Blot for Figure 8B (Pro-Cas3)

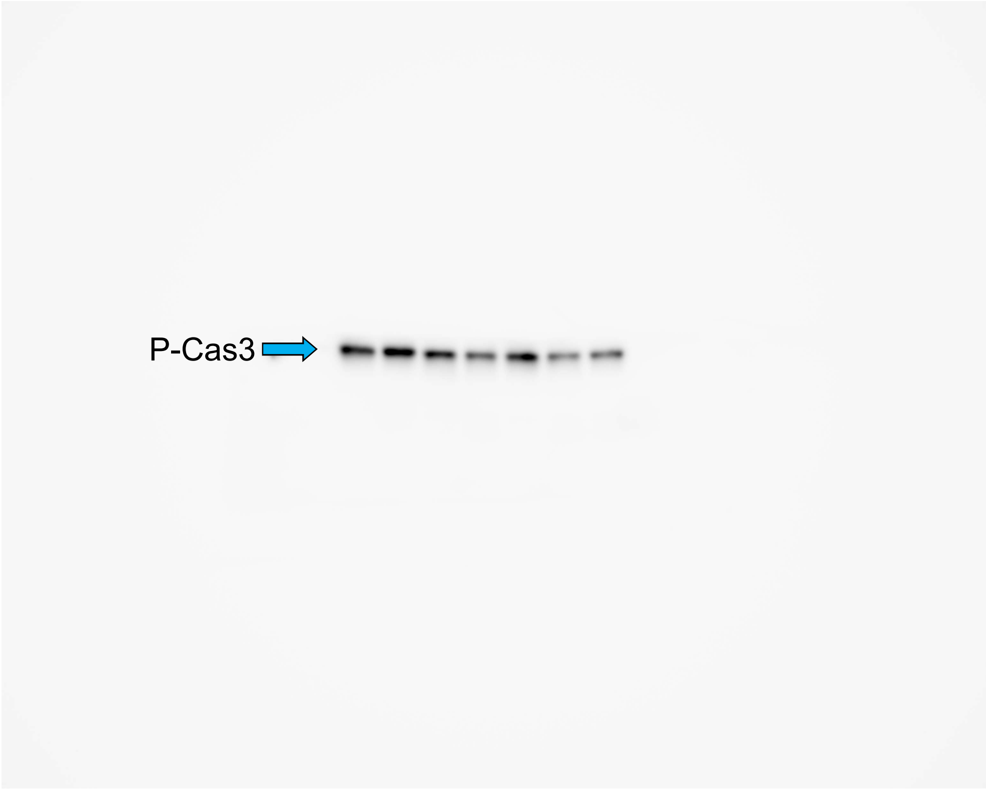

Full and uncropped Western Blot for Figure 8B (C-Cas3)

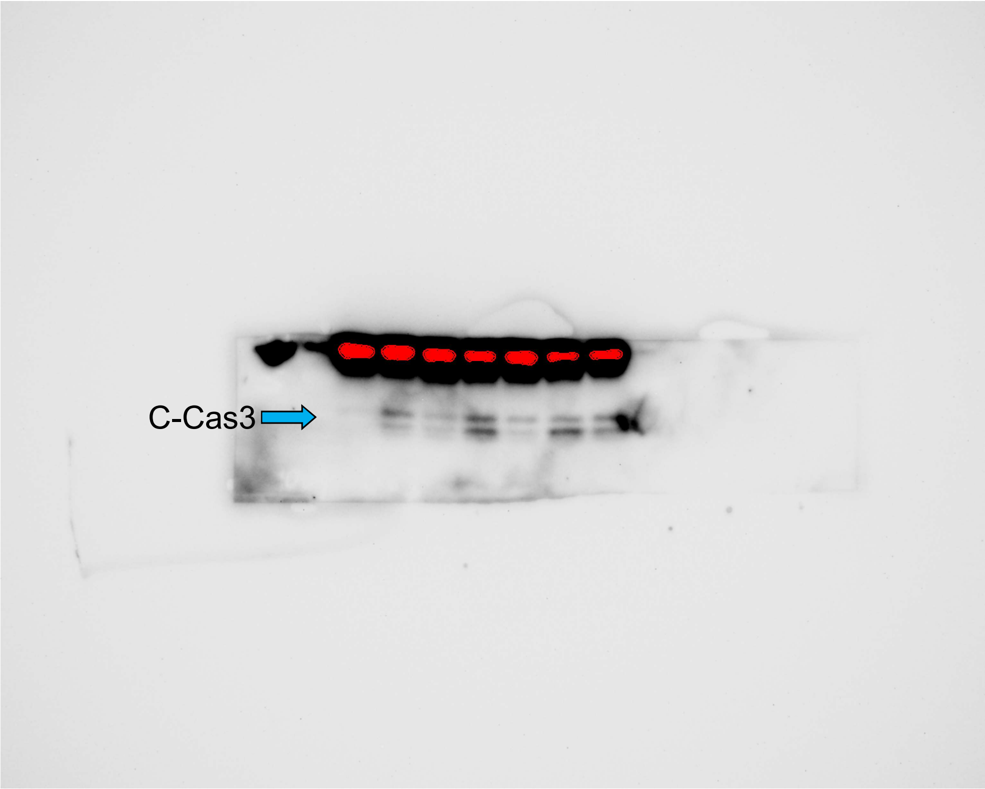

Full and uncropped Western Blot for Figure 8B (Actin)
Blots 1-7 are in the figure.

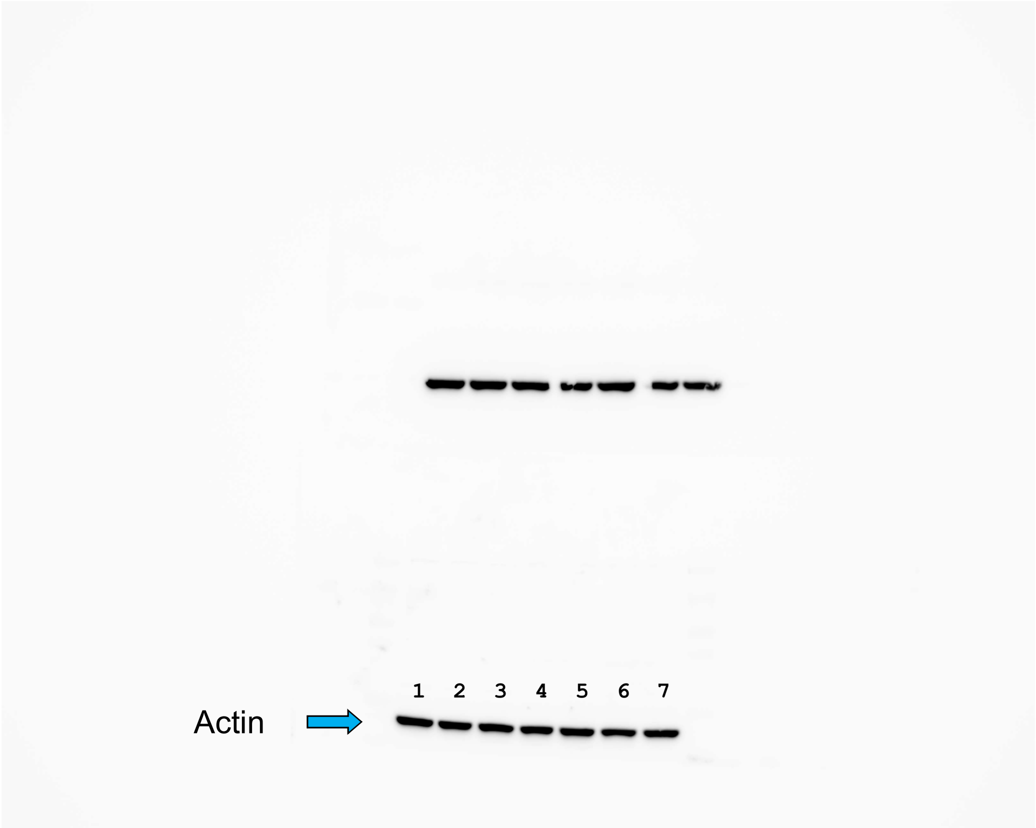

Full and uncropped Western Blot for Figure 8E (PERK)
Blots 1-8 are in the figure.

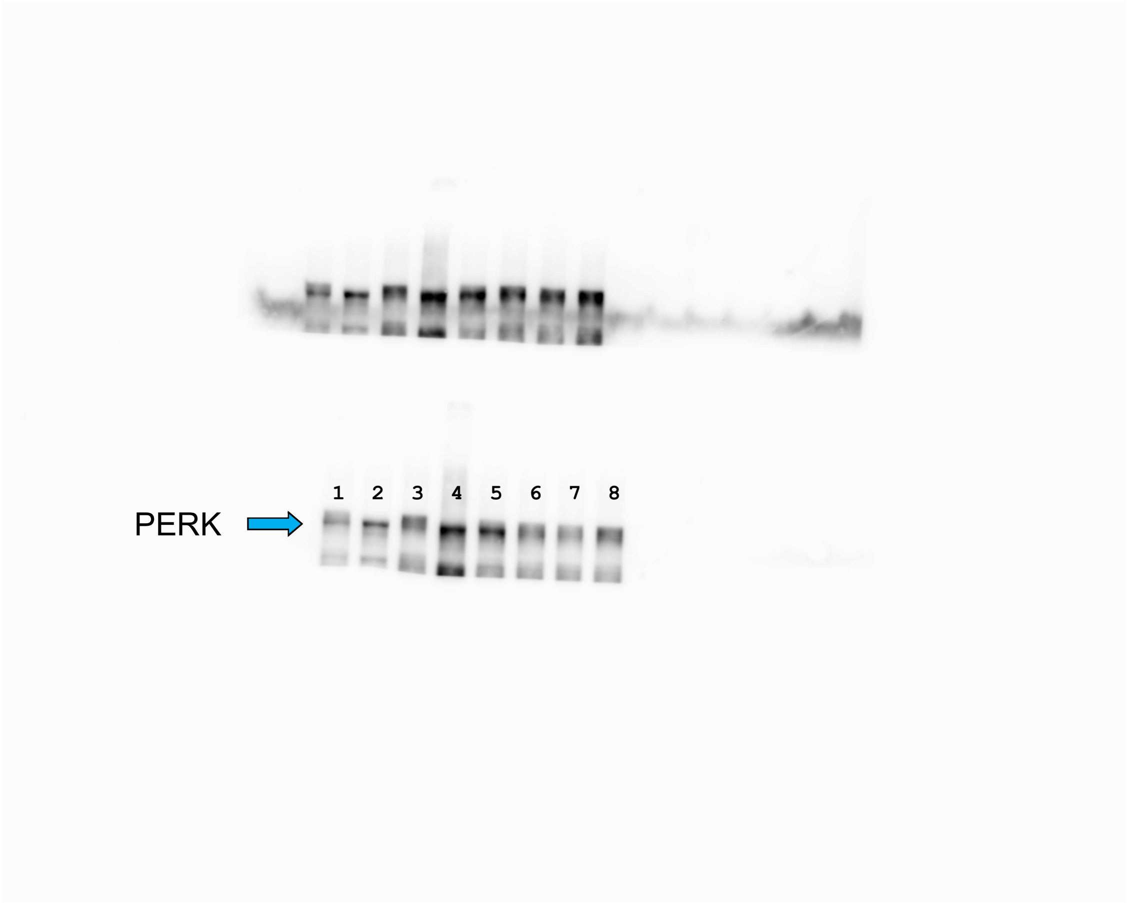

Full and uncropped Western Blot for Figure 8E (P-eif2a)
Blots 1-8 are in the figure.

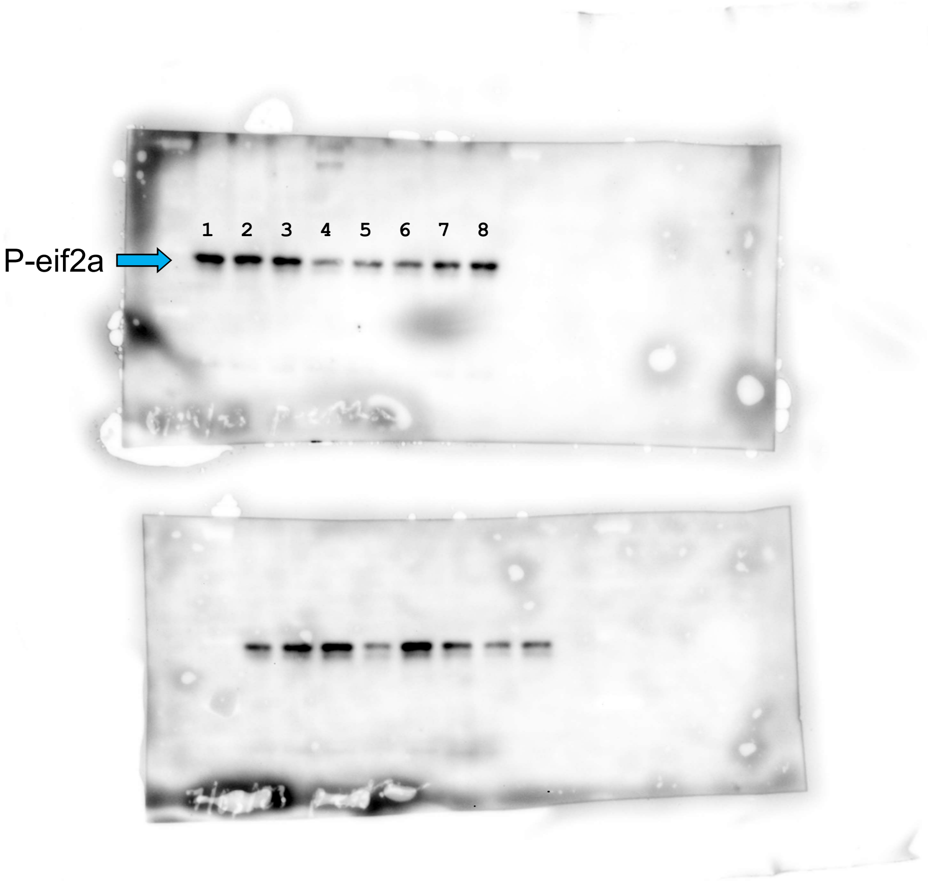

Full and uncropped Western Blot for Figure 8E (eif2a)

Blots 1-8 are in the figure.

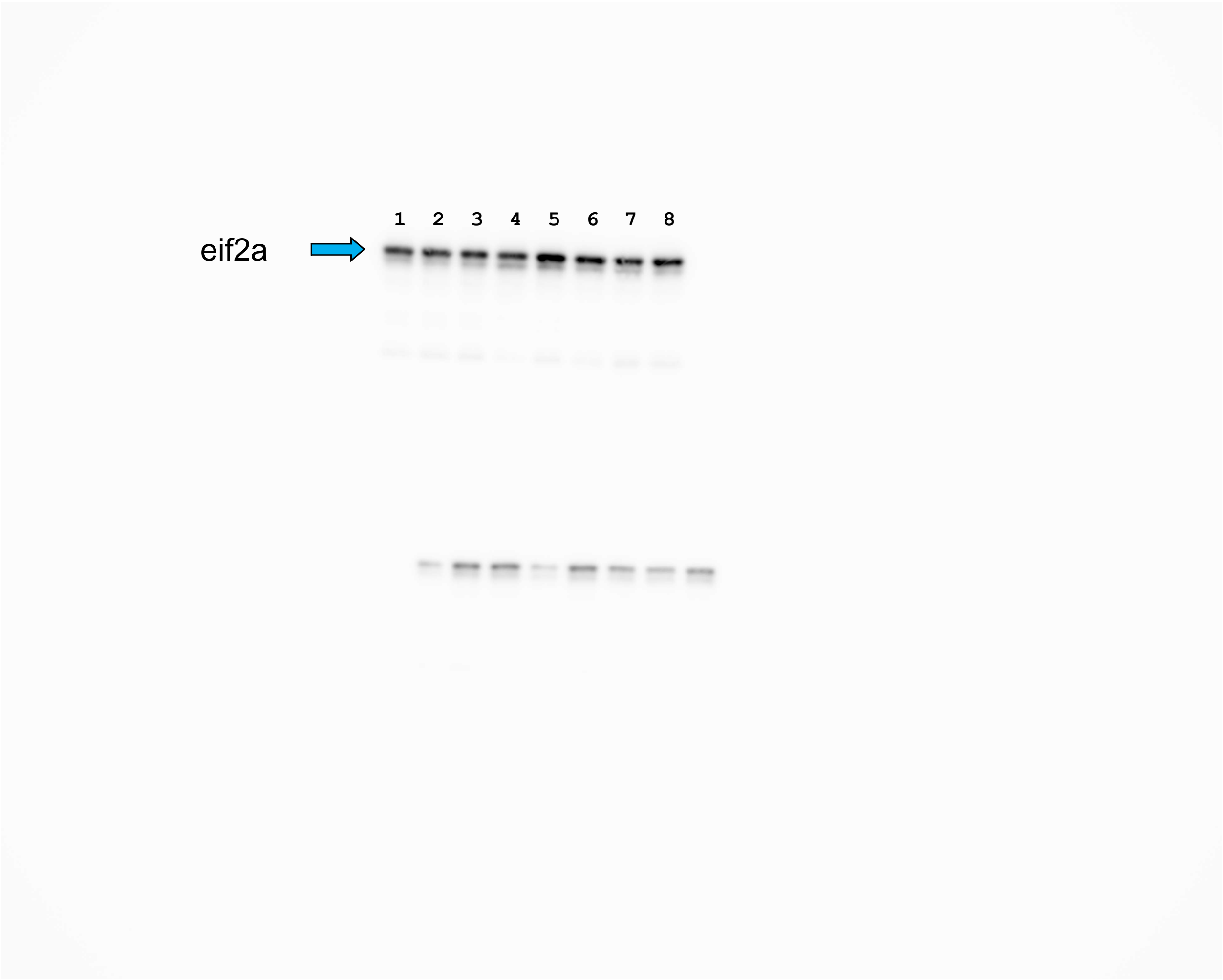

Full and uncropped Western Blot for Figure 8E (Actin)

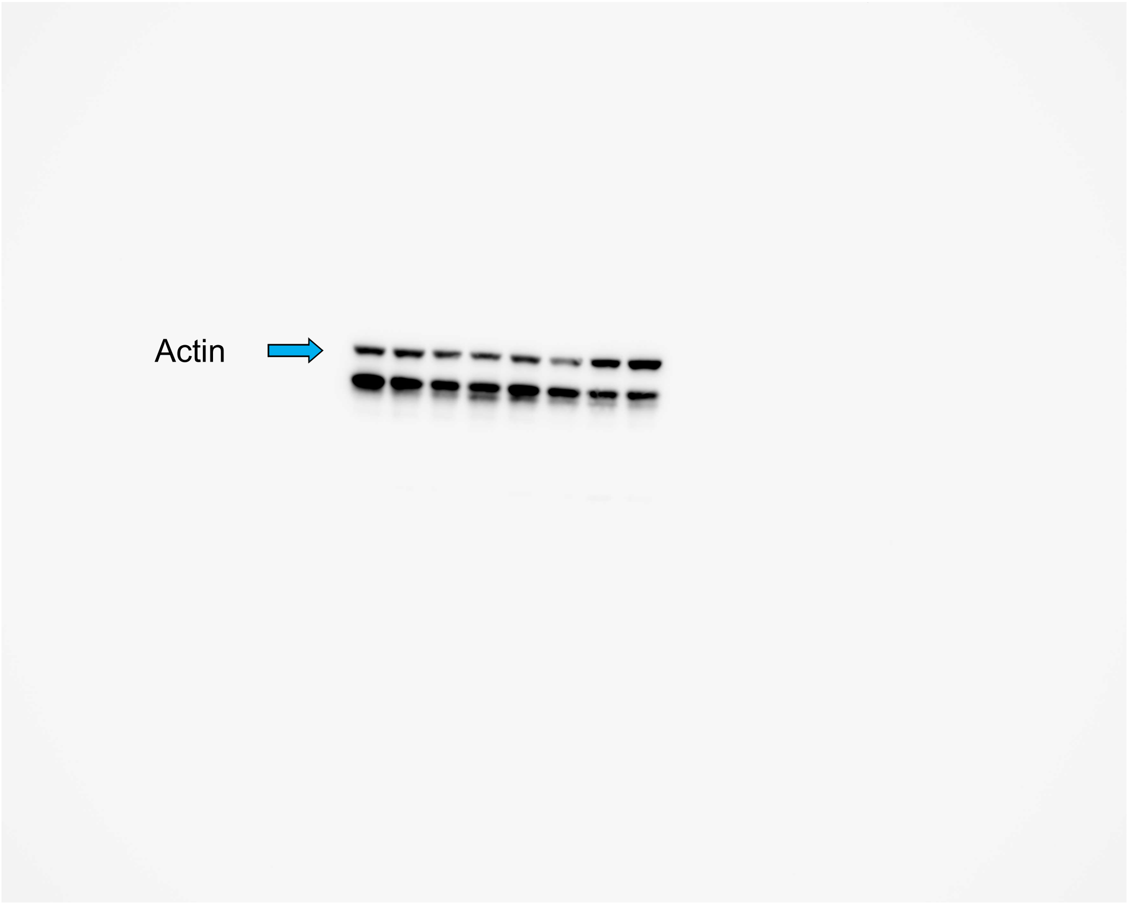

Full and uncropped Western Blot for S.Fig. 1 (PERK)

Blots 1-11 are on the Figure

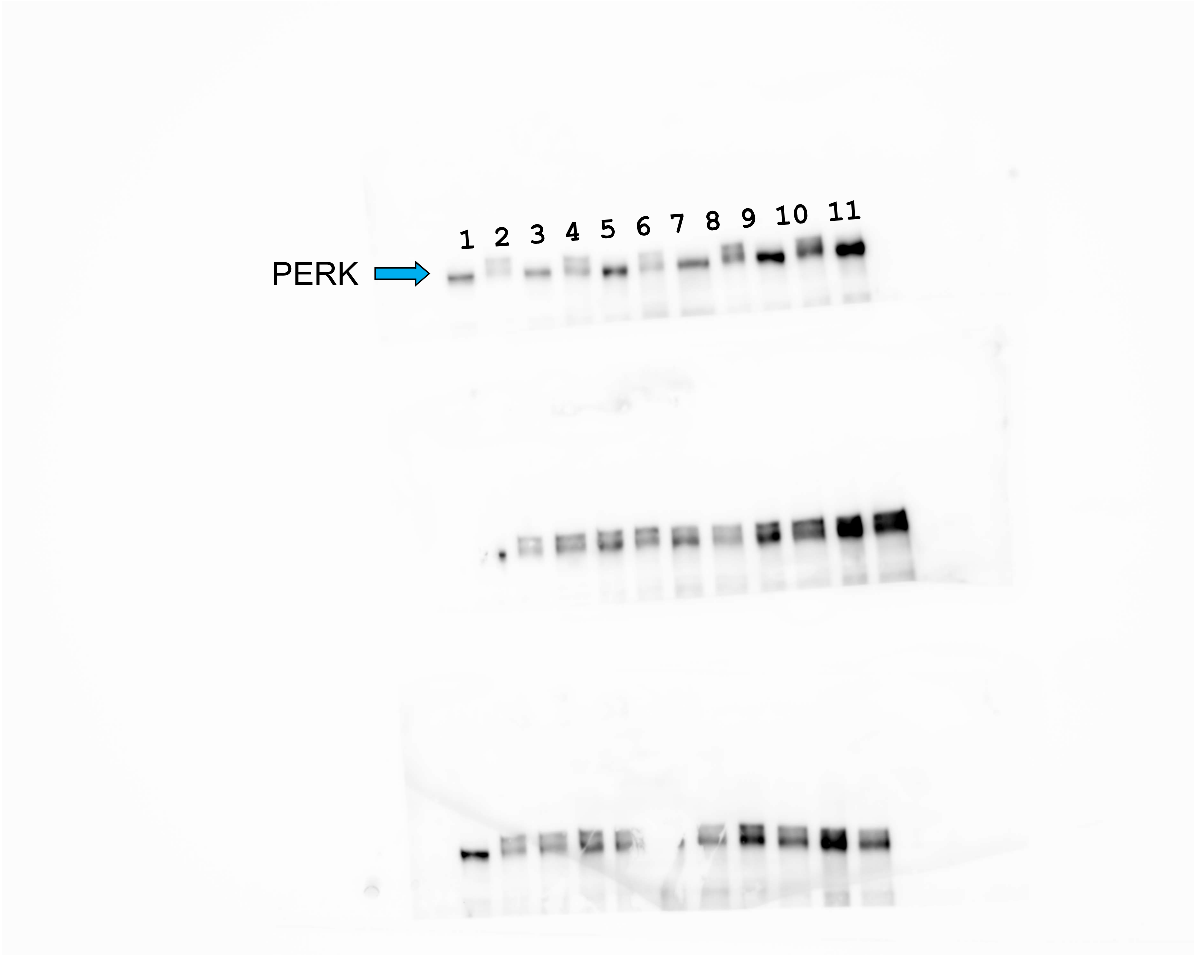

Full and uncropped Western Blot for S.Fig. 1 (P-eif2a)
Blots 1-11 are in the figure.

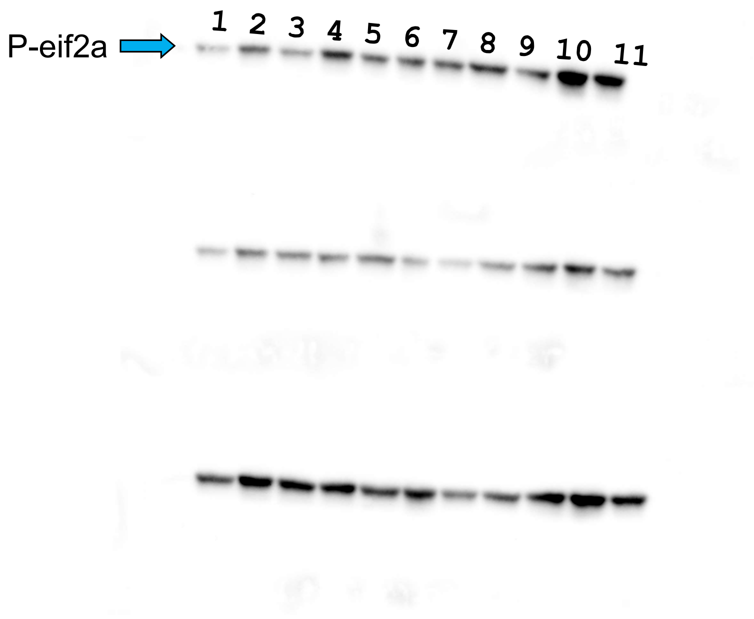

Full and uncropped Western Blot for S.Fig. 1 (eif2a)
Blots 1-11 are in the figure.

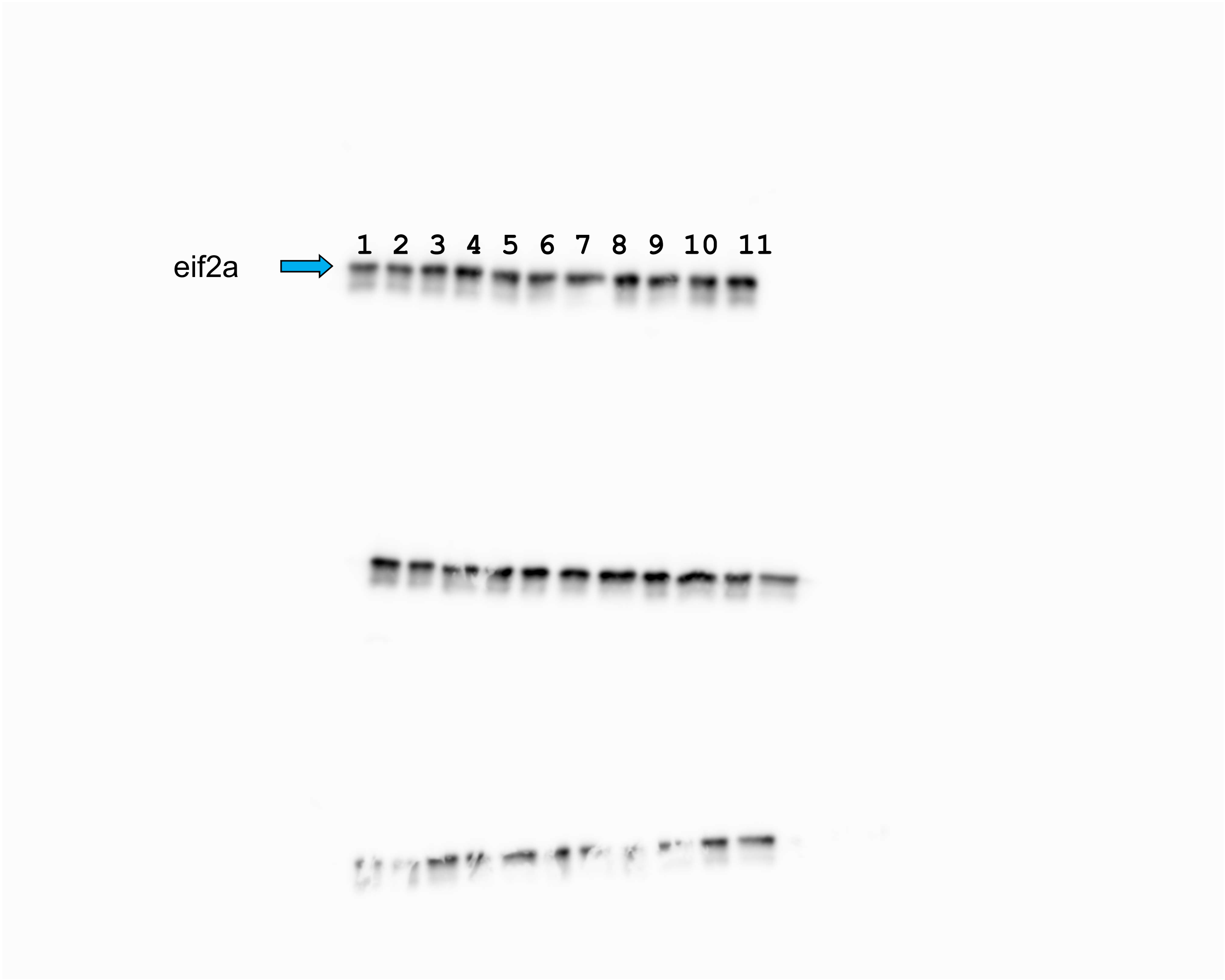

Full and uncropped Western Blot for S.Fig. 1 (IRE1)

Blots 1-11 are in the figure.

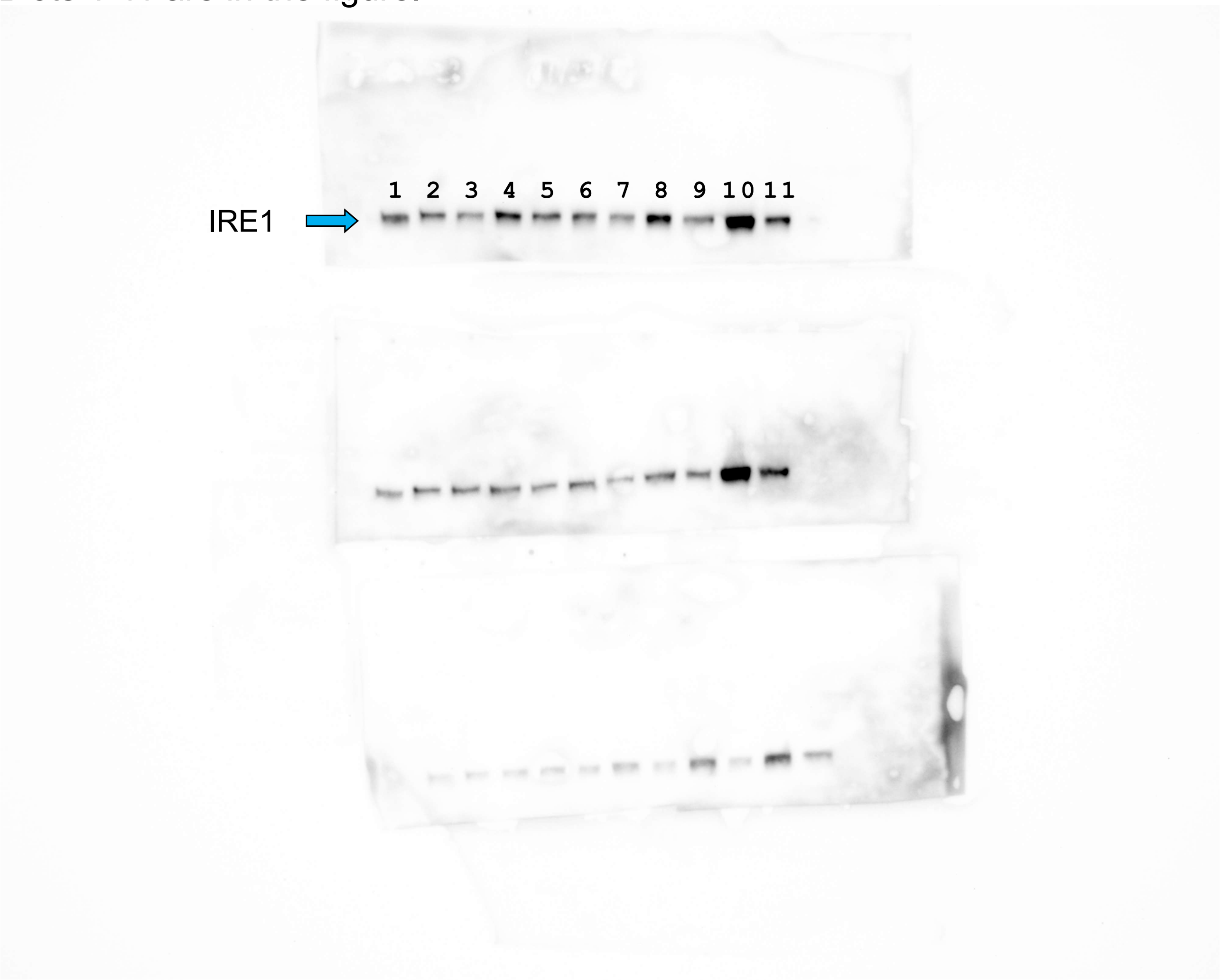

Full and uncropped Western Blot for S.Fig. 1 (XBP1s)
Blots 1-11 are in the figure.

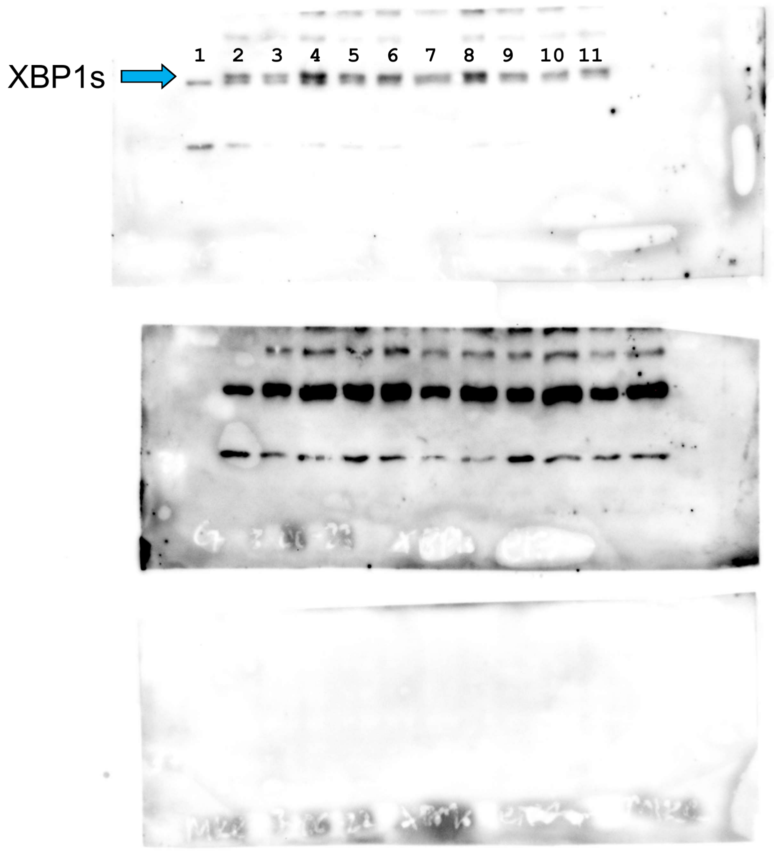

Full and uncropped Western Blot for S.Fig. 1 (Actin)
Blots 1-11 are in the figure.

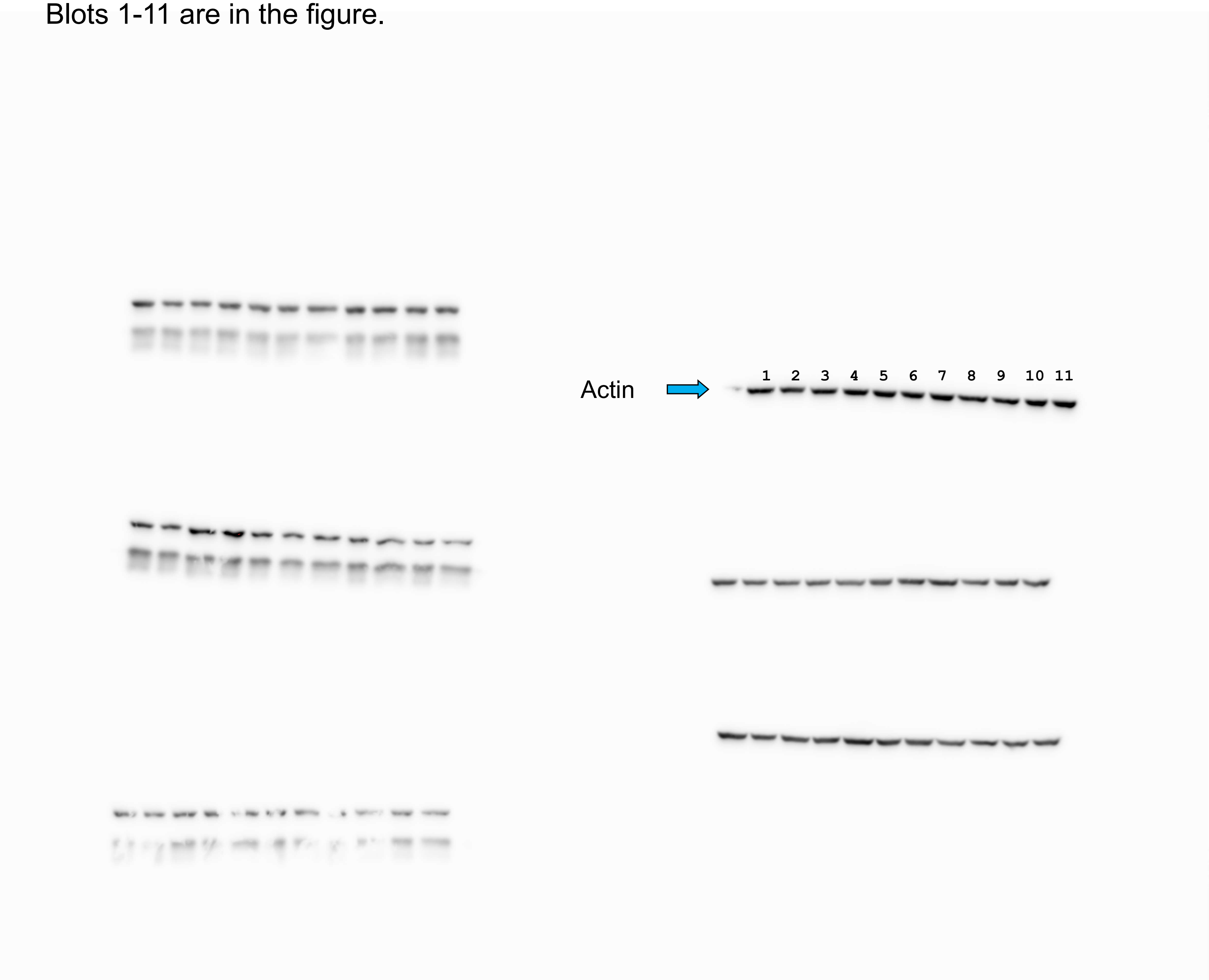

Supplement: Supplementary file 2 — Uncropped blots [file 41419_2024_6663_MOESM2_ESM.pdf]
